# Emergence and Dissemination of NDM+OXA-48-like Co-Producing *Klebsiella pneumoniae* in a Regional Healthcare Network: Seven-Year Surveillance from Latium, Italy

**DOI:** 10.3390/antibiotics15080766

**Published:** 2026-08-10

**Authors:** Carolina Venditti, Claudia Rotondo, Claudia Maestripieri, Claudia Caparrelli, Ornella Butera, Michele Properzi, Carla Nisii, Silvia D’Arezzo, Marina Selleri, Matteo Cervoni, Gilda Tonziello, Paola Scognamiglio, Andrea Siddu, Carla Fontana

**Affiliations:** 1 Microbiology and Biobank Unit, National Institute for Infectious Diseases “Lazzaro Spallanzani”, IRCCS, 00149 Rome, Italy; carolina.venditti@inmi.it (C.V.); claudia.rotondo@inmi.it (C.R.); claudia.maestripieri@inmi.it (C.M.); claudia.caparrelli@inmi.it (C.C.); ornella.butera@inmi.it (O.B.); michele.properzi@inmi.it (M.P.); silvia.darezzo@inmi.it (S.D.); marina.selleri@inmi.it (M.S.); carla.fontana@inmi.it (C.F.); 2 UOS Technical Health Professions, National Institute for Infectious Diseases “Lazzaro Spallanzani”, IRCCS, 00149 Rome, Italy; 3 Regional Service for Surveillance and Control of Infectious Diseases (SERESMI)—Lazio Region, National Institute for Infectious Diseases “Lazzaro Spallanzani”, IRCCS, 00149 Rome, Italy; matteo.cervoni@inmi.it (M.C.); gilda.tonziello@inmi.it (G.T.); paola.scognamiglio@inmi.it (P.S.); 4 Directorate for Health and Social Policy, Lazio Region, 00145 Rome, Italy; asiddu@regione.lazio.it

**Keywords:** carbapenem-resistant *Klebsiella pneumoniae*, *Klebsiella pneumoniae* ST147 NDM and OXA-48-like co-producing, Resistome, virulome, surveillance

## Abstract

**Background/Objectives**: The epidemiology of carbapenem-resistant *Klebsiella pneumoniae* (CR-Kp) in Europe is evolving towards an increasing contribution of metallo-β-lactamases (MBLs). In particular, co-production of New Delhi metallo-β-lactamase (NDM) and OXA-48-like carbapenemases represents a major concern due to limited therapeutic options and epidemic potential. We aimed to describe temporal trends and genomic characteristics of NDM and OXA-48-like co-producing *K. pneumoniae* within a regional surveillance programme targeting ceftazidime-avibactam (CZA)-resistant carbapenem-resistant *Enterobacterales* (CRE) in the Latium Region, Italy. **Methods:** Between January 2019 and December 2025, CZA-resistant CRE isolates were collected through a regional surveillance network. Antimicrobial susceptibility testing and carbapenemase detection were performed, and NDM-producing *K. pneumoniae* (NDM-Kpn) was analysed by whole-genome sequencing (WGS). Genomic analyses included multi-locus sequence typing, assessment of clonal relatedness, and resistome/virulome profiling. **Results**: A total of 2752 non-repetitive CZA-resistant CRE were collected. The analysis of CZA-resistant *K. pneumoniae* isolates submitted to the regional surveillance network showed that the proportion of NDM producers increased markedly from 2023 onwards. In particular, NDM in association with OXA-48-like reached 26.0% in 2024 and 43.6% in 2025, becoming the predominant carbapenemase profile within this selected surveillance population. WGS of 437 NDM-Kpn revealed a structured population dominated by Sequence Type (ST)147 (63.2%), widely disseminated across 24 hospitals and characterised by a predominant NDM-1 variant and OXA-48-like co-producing profile associated with KL10/*wzi*420 capsular type. A subset of isolates, mainly within the ST147-KL64 subgroup, showed higher virulence scores, indicating a possible convergence of resistance and virulence. **Conclusions**: Our findings indicate a rapid shift towards NDM-mediated resistance among CZA-resistant *K. pneumoniae* submitted to the regional surveillance network, with the emergence of NDM and OXA-48-like co-producing isolates associated with a dominant ST147 clone detected across multiple hospitals. These results highlight the urgent need for coordinated genomic surveillance and infection prevention strategies.

## 1. Introduction

Carbapenem-resistant *Enterobacterales* (CRE) represent a major global public health threat, being responsible for healthcare-associated outbreaks with high mortality rates [1]. Among CRE, carbapenem-resistant *Klebsiella pneumoniae* (CR-Kp) is one of the most clinically relevant pathogens, particularly in healthcare settings [2,3].

In Italy, the epidemiology of carbapenem resistance has long been dominated by KPC-producing *K. pneumoniae* isolates [4,5], for which the β-lactam/β-lactamase inhibitor combination ceftazidime–avibactam (CZA) has shown high clinical efficacy [6].

However, the extensive use of CZA has been paralleled by the emergence of resistance-associated mutations in different regions of the *bla*_KPC_ gene, as well as by the increasing clinical relevance of intrinsically resistant isolates producing metallo-β-lactamases (MBLs) [7,8,9].

After the large outbreak of NDM-producing *K. pneumoniae* (NDM-Kpn) reported in Tuscany in 2018–2019 [10], growing attention has been directed towards MBL-producing strains. Since then, several regional outbreaks have been described, documenting the progressive spread of NDM-Kpn across Italy and underscoring the contribution of inter-facility transmission within interconnected healthcare networks [11,12,13,14,15,16,17].

Another important concern is the global dissemination of several *K. pneumoniae* sequence types (STs), including ST147, ST11, and ST383, associated with the emergence and dissemination of NDM-producing strains [16,18,19].

Among these, ST147 and the related clonal group (CG)147 (which includes ST147 together with related sequence types such as ST273 and ST392) are recognised as high-risk international clones that have played a major role in the rapid spread of multidrug-resistant (MDR) *K. pneumoniae* in Europe and Italy [20,21,22].

More recently, some of these successful clones have also been increasingly associated with co-production of OXA-48-like carbapenemases, a combination that further restricts therapeutic options and may enhance epidemic potential [23,24,25].

Despite these observations, long-term datasets integrating temporal trends, clonal structure, resistance determinants, and virulence features of NDM- and OXA-48-like co-producing *K. pneumoniae* at the international, national, and regional levels remain limited.

Within this evolving epidemiological scenario, in 2019 the National Institute for Infectious Diseases “Lazzaro Spallanzani” (INMI) IRCCS established a seven-year regional surveillance programme in the Latium Region, aiming to collect all CZA-resistant CRE isolates from clinical microbiology laboratories across the region. By combining microbiological characterization with whole-genome sequencing (WGS), we sought to define temporal trends, clonal expansion, and the resistance and virulence profiles of NDM- and OXA-48-like co-producing *K. pneumoniae*, thereby providing evidence to strengthen surveillance and support antimicrobial resistance (AMR) control strategies.

## 2. Results

### 2.1. Overview of the Collected CZA-Resistant CRE Isolates

In 2019, following the “European Centre for Disease Prevention and Control” (ECDC) alert [26], in an effort to prevent the spread of CZA-resistant CRE, and in our role as regional reference centre, we began collecting and analysing all CZA-resistant CRE isolates obtained from patients treated at our hospital, as well as strains received from other hospitals of the Latium Region. Twenty-eight clinical microbiology laboratories contributed to the regional surveillance programme, yielding 3093 isolates overall, which corresponded to 2752 non-repetitive, single-patient clinical strains of CZA-resistant CRE.

The collection comprised 18 species, most of which belonged to the *Klebsiella* genus (2354/2752; 85.5%), including *K. pneumoniae* (n = 2324, 84.45%), *K. oxytoca* (n = 13, 0.47%), *K. variicola* (n = 8, 0.29%), *K. aerogenes* (n = 6, 0.22%) and *K. ozaenae* (n = 3, 0.11%); *Escherichia coli* accounted for 265 isolates (9.6%). Other less frequently recovered species included *Enterobacter* spp. (n = 65, 2.6%; 39 *E. cloacae*, 23 *E. hormaechei*, two *E. bugandensis* and one *E. kobei*), *Citrobacter* spp. (n = 30, 1.09%; 27 *C. freundii* and three *C. koseri*), *Proteus mirabilis* (n = 25, 0.91%), *Providencia* spp. (n = 8, 0.29%; four *P. rettgeri* and four *P. stuartii*), *Raoultella* spp. (n = 4, 0.14%; three *R. ornithinolytica* and one *R. planticola*) and *Morganella morganii* (n = 1, 0.04%) (Table S1, Panel A).

Focusing on CR-Kpn, we analysed the distribution of specimen types within this subgroup. Rectal swabs were the most frequent (1277/2324; 55.0%), followed by urine (525/2324; 22.6%) and blood cultures (235/2324; 10.1%). Less frequent sources included bronchoalveolar lavage (58; 2.5%), wound swabs (57; 2.5%), catheter urine (48; 2.1%), catheter-drawn blood cultures (27; 1.2%), bronchial aspirates (26; 1.1%), and sputum (18; 0.8%). All remaining specimen types accounted for less than 1% of isolates.

### 2.2. Resistance Phenotypes

All 2752 isolates included in the surveillance were resistant to CZA. Across the overall collection, the epidemiology was largely driven by the two most-represented species, *K. pneumoniae* (2324/2752; 84.45%) and *Escherichia coli* (265/2752; 9.63%), which showed partially overlapping yet distinct carbapenemase production patterns over time (Table 1, Tables S1 and S2).

**Figure 1:**
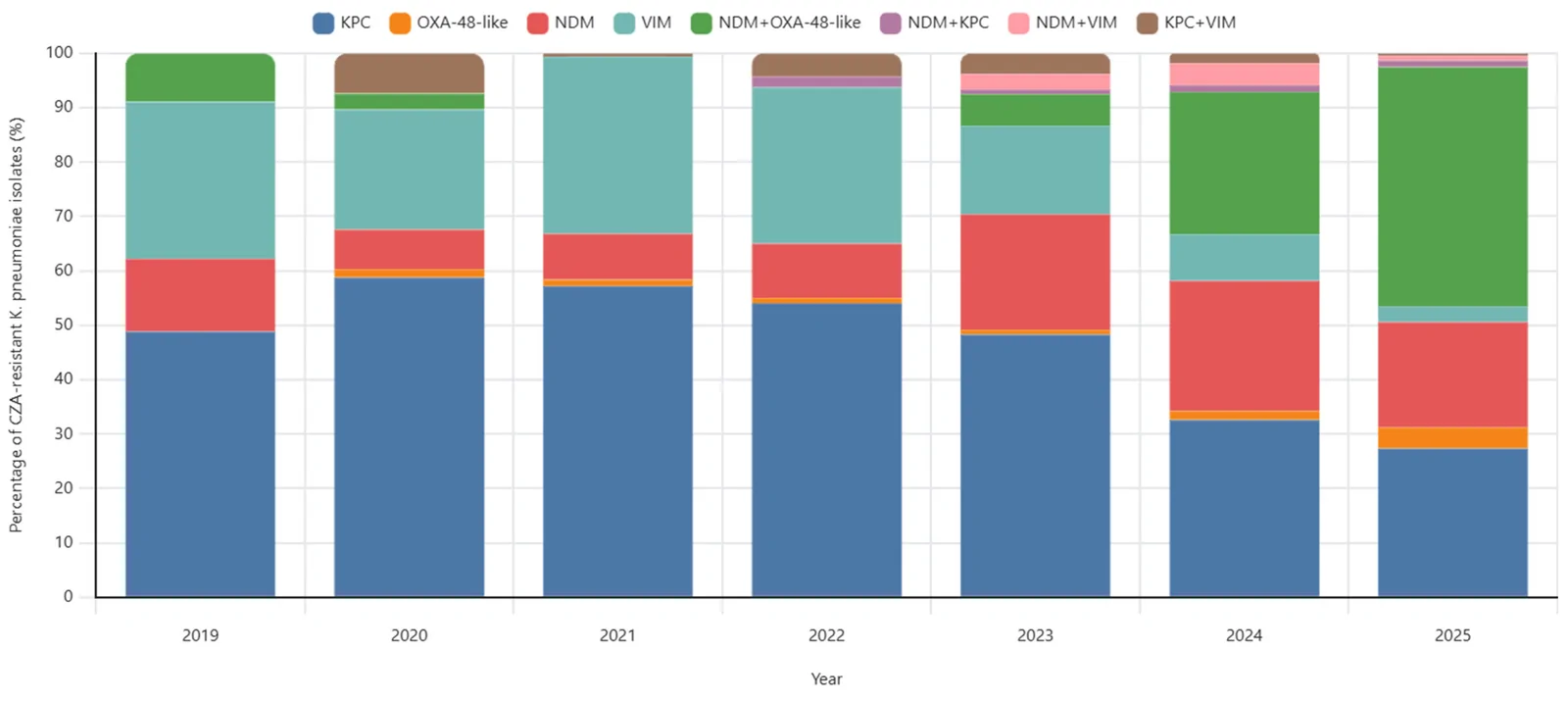
Yearly distribution of carbapenemase profiles among CZA-resistant *Klebsiella pneumoniae* isolates submitted to the regional surveillance network (2019–2025). Yearly distribution of carbapenemase profiles among CZA-resistant *K. pneumoniae* isolates submitted to the Latium regional surveillance network, 2019–2025. Stacked bars show the annual percentage of each carbapenemase profile among non-repetitive CZA-resistant *K. pneumoniae* isolates. Colours indicate the different resistance profiles. A progressive increase in NDM plus OXA-48-like co-producing isolates was observed from 2023 onwards, reaching 43.6% in 2025.

As shown in Table 1 and Table S1, KPC was the predominant carbapenemase among *K. pneumoniae*, accounting for 845/2324 isolates (36.4%) overall. Its proportion increased in the early phase of surveillance, reaching a peak of 58.8% in 2020, and then progressively declined to 27.1% in 2025. Among MBLs, VIM accounted for 254/2324 isolates (10.9%) and NDM for 426/2324 isolates (18.3%). VIM was the predominant MBL during the early years of surveillance, particularly from 2019 to 2022. However, from 2023 onwards, the epidemiological scenario changed markedly, with a progressive rise in NDM-producing isolates, from 10.0% in 2022 to 19.2% in 2025. An even more relevant finding was the sharp increase in *K. pneumoniae* isolates co-producing NDM and OXA-48-like enzymes, which accounted for 628/2324 isolates overall (27.0%). This resistance profile rose from 5.8% in 2023 to 43.6% in 2025, ultimately becoming the most prevalent carbapenemase profile by the end of the study period (Table 1 and Figure 1). Yearly proportions with Wilson 95% confidence intervals (CI) are reported in Table S1 Panel B. The temporal increase in NDM plus OXA-48-like co-producing *K. pneumoniae* was statistically significant by the Cochran-Armitage trend test (*p* < 0.0001).

A partially different pattern was observed in *E. coli*, in which MBLs clearly predominated throughout the study period. In this species, NDM was by far the most frequent determinant overall (205/265; 77.4%) and became the dominant carbapenemase from 2023 onwards, accounting for 82.4% of isolates in 2023 and 84.2% in 2025. VIM was the second most represented resistance mechanism (38/265; 14.3%), whereas KPC was uncommon throughout the study period (5/265; 1.9%) and never emerged as the leading carbapenemase profile in any study year. Co-production of NDM plus OXA-48-like was detected only in a limited number of *E. coli* isolates (9/265; 3.4%) (Table S2).

**Figure 2:**
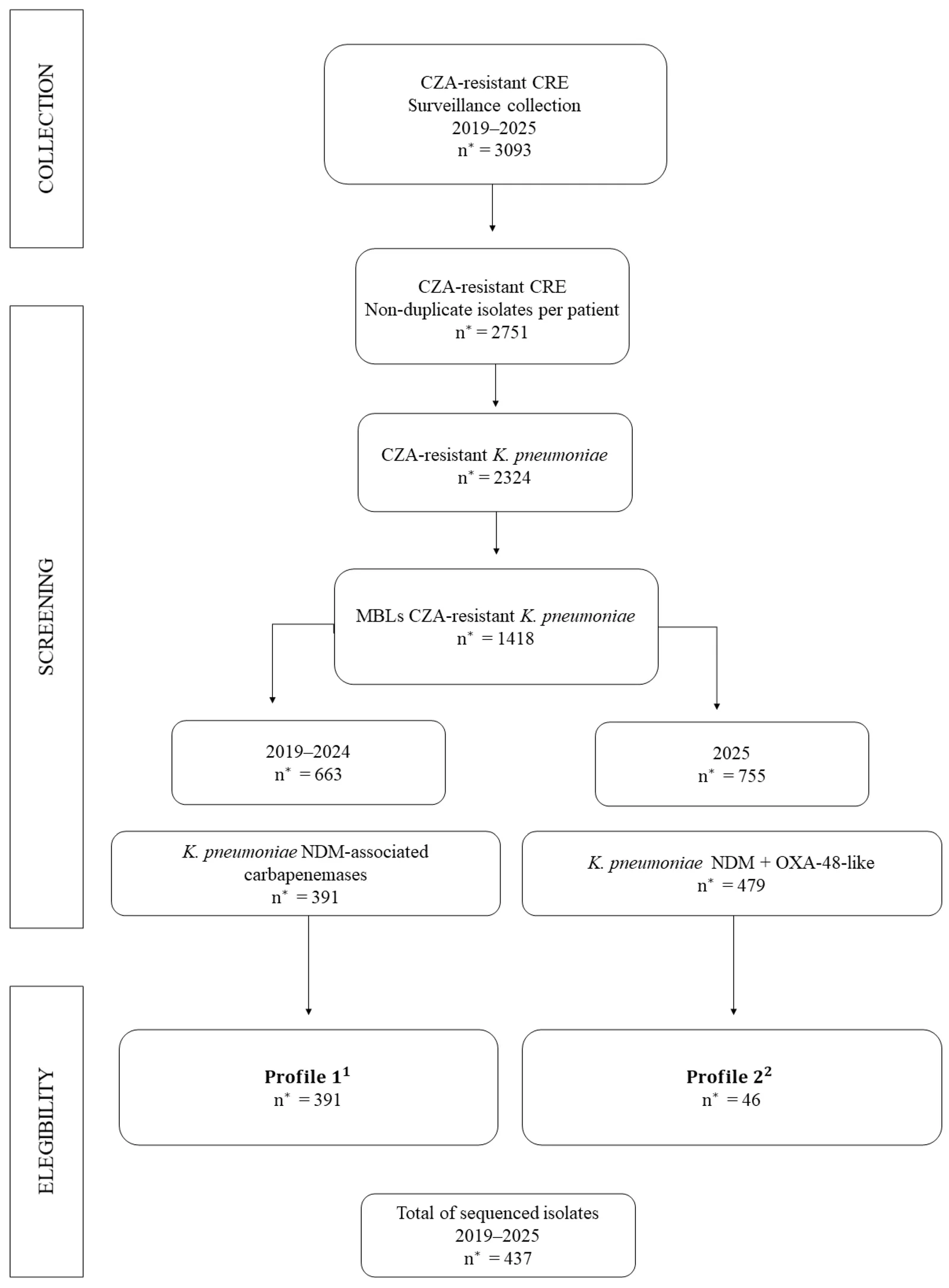
Flow chart of representative whole-genome sequencing (WGS) selection isolates. ^1^ Profile 1: *K. pneumoniae* NDM-associated carbapenemases (first isolate per patient) from 2019 to 2024. ^2^ Profile 2: *K. pneumoniae* NDM+OXA-48-like (prioritized invasive/infection-associated isolates) referred to 2025. n*: number of isolates.

Figure 2 summarises the method used to select isolates for WGS analysis (from the overall CZA-resistant CRE surveillance collection). Among CZA-resistant *K. pneumoniae* collected over the study period, isolates producing NDM alone or in combination with OXA-48-like carbapenemases were considered eligible for genomic analysis. According to the patient-based and clinically oriented sampling strategy described in Section 4, 437 NDM-Kpn isolates were selected for WGS.

Given the marked predominance of NDM-Kpn isolates in 2025, including 211/1099 (19.2%) NDM producers and 479/1099 (43.6%) NDM plus OXA-48-like co-producers (Table 1), subsequent genomic analyses focused on this selected WGS dataset to characterize clonal relationships, resistance determinants, and virulence features.

All isolates displayed an extensively drug-resistant (XDR) phenotype, with resistance to carbapenems (meropenem, ertapenem and imipenem), high rates of resistance to ciprofloxacin (98.8%), piperacillin–tazobactam (99.7%) and trimethoprim–sulfamethoxazole (82.1%), and variable resistance to gentamicin and amikacin (84.2% and 90.4%, respectively).

### 2.3. Molecular Typing of Selected NDM-Producing K. pneumoniae Isolates

All 437 sequenced isolates carried a *bla*_NDM_ gene, predominantly *bla*_NDM-1_ (390/437, 89.2%) and, less frequently, *bla*_NDM-5_ (47/437, 10.8%). Additional β-lactam resistance determinants were widely distributed, including *bla*_CTX-M-15_ (398/437, 91.1%) and *bla*_OXA-48-like_ genes (197/437, 45.1%). A subset of isolates also harboured additional carbapenemases, including *bla*_VIM-1_ (5.9%) and *bla*_KPC-3_ (2.7%) (Table S3).

Beyond β-lactam resistance, WGS analysis revealed a broad repertoire of additional resistance determinants consistent with an XDR phenotype. Genes conferring resistance to aminoglycosides, quinolones, sulfonamides, trimethoprim, macrolides, tetracyclines and fosfomycin were commonly detected, with most isolates carrying multiple resistance determinants across several antimicrobial classes (Table S3).

Molecular typing of the 437 isolates revealed a highly structured population, in which 26 STs appeared distributed across 28 hospitals. ST147 was by far the predominant lineage, accounting for 276/437 isolates (63.2%), and showing the widest distribution across the surveillance network, followed by ST11 (88/437; 20.1%), ST512 (14/437; 3.2%) and ST1805 (12/437; 2.7%). Less frequent lineages included ST395 (9/437; 2.1%), ST383 (7/437; 1.6%), ST15 (5/437; 1.1%), ST6668 (4/437; 0.9%), ST307, ST14, ST20 and ST unknown (two isolates each), while several additional STs (ST16, ST17, ST23, ST25, ST29, ST39, ST200, ST234, ST1117, ST3299, ST4081, ST4853, ST6118, and ST8609) were represented by single isolates. Two isolates could not be assigned to any ST (Table 2).

In addition to defining clonal relationships and resistance determinants, WGS analysis also enabled the characterization of virulence-associated loci and virulence scores across the major circulating clones.

Virulence profiling revealed marked differences among the major epidemic clones. Most ST147 isolates exhibited a low virulence profile, with 219/276 (79.3%) showing a virulence score of 1, predominantly associated with yersiniabactin loci. This pattern was largely driven by the dominant KL10 subgroup, typically characterized by *ybt1*/*ICEKp4* and lacking additional hypervirulence-associated determinants. In contrast, a subset of 45/276 ST147 isolates (16.3%) displayed higher virulence scores (score 4) and were mainly associated with the KL64 subgroup, in which *ybt9*/*ICEKp3* frequently co-occurred with aerobactin (*iuc1*) and *rmp*-associated loci, indicating a convergent resistance–virulence profile.

Compared with ST147, ST11 isolates showed a more homogeneous and overall lower virulence profile, with 85/88 (96.6%) displaying a virulence score of 1, typically associated with *ybt15*/*ICEKp11*. Similarly, ST512 remained largely low-virulence, with *ybt8*/*ICEKp9* and a virulence score of 1 predominating.

By contrast, ST1805, although less prevalent, showed a comparatively higher virulence potential, with 11/12 isolates displaying a virulence score of 3. Among minor STs, ST395 was also notable for its high virulence profile, with the majority of isolates (8/9) exhibiting a virulence score of 4, whereas ST383 combined frequent co-production of NDM and OXA-48-like carbapenemases with additional virulence-associated determinants, including *iuc1* and *rmp1*, consistent with a convergent resistance–virulence phenotype (Table S4).

Core-genome multi-locus sequence typing (cgMLST) analysis further resolved the population into 25 CTs, highlighting a highly structured clonal architecture. ST147 showed the widest dissemination across the surveillance network, being detected in 24 hospitals and mainly distributed across nine defined CTs (CT-1 to CT-9). The population was largely dominated by a major cgMLST cluster, CT-1, comprising 166 isolates overall, including 165 ST147 isolates and one ST8609 isolate, followed by CT-2 (44 isolates), CT-3 (30 isolates) and CT-4 (12 isolates) (Table S5).

CT-1 represented the predominant cluster, comprising 166 isolates overall and spanning 17 hospitals. This cluster showed a homogeneous genomic profile, frequent co-production of NDM and OXA-48-like carbapenemases, and predominantly low virulence scores.

In contrast, other ST147 lineages displayed greater heterogeneity, including clusters associated with higher virulence scores (CT-2), additional carbapenemase determinants such as *bla*_VIM-1_ (CT-3), or alternative resistance profiles driven by *bla*_NDM-5_ (CT-4) (Tables S4 and S5).

Within ST147, co-production of NDM and OXA-48-like carbapenemases represented the main resistance mechanism, accounting for approximately two-thirds of isolates, and was mainly associated with the KL10/*wzi*420 capsular profile, whereas the KL64 subgroup was more frequently associated with higher virulence scores (Table S4).

This distribution is in line with the known phylogenetic structure of CG147, in which ST147 is divided into major KL64 and KL10 clades with distinct evolutionary histories and genomic features.

ST11 represented the second most prevalent clone and was identified in 12 hospitals. In contrast to the broader heterogeneity observed in ST147, ST11 displayed a more compact clonal structure, with most isolates clustering within a single major CT (CT-10, 81/88 isolates) and showing a strong association with the KL24 capsular locus (83/88; 94.3%). Although *bla*_NDM-1_ alone was the dominant resistance determinant, a limited number of isolates carried more complex carbapenemase combinations, including NDM plus OXA-48-like or NDM plus KPC-3 (Figure S1 and Table S4).

ST512 and ST1805 showed more limited dissemination. ST512 comprised 14 isolates distributed across two clusters (CT-12 and CT-13) and was consistently associated with KL107, whereas ST1805 included 12 isolates belonging to a single cluster (CT-11), all associated with KL48 and characterized by intermediate virulence scores (Figure S1 and Table S4).

Additional minor clones also suggested inter-hospital circulation. ST383 was consistently associated with KL30 and frequently displayed co-production of NDM and OXA-48-like carbapenemases, whereas ST395 was mainly linked to KL2 and characterized by a higher virulence profile (Figure S1 and Table S4).

## 3. Discussion

The progressive emergence of NDM-Kpn represents a major public health concern worldwide [23]. In this seven-year regional surveillance study of CZA-resistant CRE, we documented a marked shift in carbapenemase profiles, characterized by the rapid increase in NDM-Kpn and in isolates co-producing NDM and OXA-48-like carbapenemases (Table 1 and Figure 1). By 2025, this combined resistance profile had become the main carbapenemase mechanism observed in the CZA-resistant *K. pneumoniae* isolates included in our surveillance collection.

This transition was clearly reflected in the temporal distribution of resistance mechanisms (Table 1 and Figure 1). While KPC-producing isolates predominated during the early phase of surveillance, consistent with their long-standing endemicity in Italy [5,26], their prevalence progressively declined over time. In parallel, VIM-type MBLs played a major role in the initial years but were subsequently overtaken by NDM-producing isolates. From 2023 onwards, NDM became increasingly dominant, culminating in a sharp rise in NDM and OXA-48-like co-producing isolates, which increased from 5.8% in 2023 to 43.6% in 2025 (Table 1 and Figure 1). This trend mirrors the evolving epidemiological landscape described in Italy and other European countries, where NDM-Kpn has gained increasing prominence over the last decade [10,27,28,29,30,31,32].

Genomic analysis indicates that this epidemiological shift was largely driven by clonal expansion rather than independent horizontal acquisition of resistance determinants. ST147 was the predominant clone, accounting for over 60% of sequenced isolates, and showing extensive dissemination across the regional surveillance network, followed by ST11 and ST1805 (Table 2 and Table S4). These clones are widely recognized as international high-risk lineages shaping the epidemiology of CRE [11,13,14,22,30].

Taken together, these findings support the role of ST147 as the main epidemic lineage driving the regional spread of carbapenem-resistant *K. pneumoniae* within the healthcare network.

Notably, ST147 showed a structured clonal composition, with a predominant cgMLST cluster, CT-1, characterized by distinct resistance or virulence features (Table S5). The detection of CT-1 across 17 hospitals, together with its homogeneous genomic profile and frequent NDM plus OXA-48-like co-production, supports the interpretation of this cluster as an endemic high-risk ST147 clone stably circulating in the Latium regional healthcare setting. In the absence of patient-transfer data and detailed epidemiological links, these findings should not be interpreted as evidence of direct inter-hospital transmission.

In line with previous studies in Italy and Europe, ST147 has emerged as a high-risk clone responsible for global hospital outbreaks, further emphasising the role of healthcare networks in amplifying the regional spread of high-risk clones [32,33,34].

Within ST147, co-production of NDM and OXA-48-like carbapenemases was the predominant resistance mechanism and was strongly associated with specific capsular types, particularly the KL10/*wzi*420 lineage, followed by KL64 and, less frequently, KL51 (Table 2 and Table S4). This distribution aligns with the reported phylogenetic structure of CG147, in which ST147 is divided into two main capsular clades, KL64 and KL10; notably, the latter has been associated with NDM and OXA-48-like carbapenemases, whereas KL64 represents a globally disseminated clone with distinct genomic features [22]. Together, these observations are consistent with previous reports describing ST147 as a successful high-risk lineage able to co-harbour *bla*_NDM-1_ and *bla*_OXA-48-like_ genes, further supporting its role in the international dissemination of convergent resistance profiles [35].

The expansion of the KL10/*wzi*420 lineage further supports the emergence of well-adapted MDR lineages within ST147.

Of note, CT-1 was largely composed of NDM and OXA-48-like co-producing isolates with highly homogeneous genomic profiles (Table S5 and Figure S1), supporting its role as the main epidemic lineage.

Additional lineages were associated with distinct resistance profiles, including *bla*_NDM-5_ and *bla*_VIM-1_ variants, or with increased virulence potential (Table S4 and Figure S1). Furthermore, *bla*_NDM_ was identified across 26 distinct STs and 25 CTs (Table 2 and Table S4), suggesting that the expansion of dominant clones occurred alongside the circulation of diverse NDM-producing lineages, including novel or less common lineages such as ST6668 and ST383, the former having recently been reported as an emerging NDM-producing CC147-related clone in northern Italy [36]. However, because the present analysis was based on short-read WGS and cgMLST, the role of plasmid transmission, resistance-gene context, and horizontal gene transfer could not be assessed. Therefore, horizontal dissemination of resistance determinants can be considered a possible but unconfirmed mechanism in this setting.

Virulence profiling adds a further level of concern. While most isolates, particularly within ST147, displayed a low virulence profile, indicating that epidemic success was primarily driven by antimicrobial resistance and transmission fitness, a subset of isolates showed features of convergent evolution (Table 2 and Table S4). In particular, ST147 isolates belonging to the KL64 subgroup (16.3%) exhibited higher virulence scores and carried additional virulence determinants, including aerobactin (*iuc1*) and *rmp*-associated loci. This pattern is consistent with previous reports describing the convergence of resistance and virulence in *K. pneumoniae* [13,37].

Similar trends were observed in less prevalent STs, such as ST395, which showed a high virulence profile in the majority of isolates, and ST383, which combined frequent NDM and OXA-48-like co-production with additional virulence-associated determinants, including *iuc1* and *rmp1* (Table S4 and Figure S1). This finding of isolates with a convergent resistance-virulence phenotype raises concerns about the potential emergence of clinically impactful clones.

Some limitations of our study should be noted. First, the surveillance programme was restricted to CZA-resistant CRE, potentially introducing a selection bias favouring MBL-producing isolates. Therefore, the temporal trends reported here should be interpreted as reflecting the distribution of carbapenemase profiles among CZA-resistant CRE, and particularly CZA-resistant *K. pneumoniae* submitted to the regional surveillance network, not necessarily depicting the overall regional burden of CRE or CR-Kp.

Second, although the network covered a large regional healthcare system, the findings may not be fully generalisable to other settings. Third, because of the large number of strains collected, WGS was performed on a subset of isolates, possibly underrepresenting minor lineages.

Furthermore, detailed patient-level clinical information, including prior antimicrobial exposure, comorbidities, clinical outcomes, admission dates, epidemiological links, and inter-hospital patient-transfer data, was not systematically available across all participating centres. Consequently, although the distribution of closely related cgMLST clusters across multiple hospitals is consistent with inter-facility dissemination, the clinical impact of specific genomic profiles, direct transmission events, and transmission pathways could not be formally assessed.

Future studies integrating genomic surveillance with detailed epidemiological and clinical data will be important to better understand transmission dynamics and factors associated with the successful dissemination of high-risk clones. Long-read sequencing and dedicated plasmid/gene-context analyses would also be helpful in future studies to clarify the mechanisms underlying the dissemination of *bla*_NDM_ and *bla*_OXA-48-like_ determinants.

However, given the long surveillance period, the large number of facilities involved in the network, and the substantial number of isolates analysed, we believe that our findings provide a comprehensive picture of the evolving epidemiology of CZA-resistant CRE in our region.

## 4. Materials and Methods

### 4.1. Collection of Clinical Isolates

Between January 2019 and December 2025, we carried out a regional surveillance programme aimed at collecting all CZA-resistant CRE clinical strains, including isolates resistant to CZA due to carbapenemase mechanisms not inhibited by avibactam, such as MBL production. Twenty-eight clinical microbiology laboratories, distributed across the Latium Region and covering most Roman hospitals, participated in the survey. All isolates were re-evaluated in our laboratory for species identification and characterization of the carbapenem resistance mechanisms. WGS of a selected subset of 437 CR-Kpn isolates was performed to provide a comprehensive overview of bacterial circulation, virulence patterns, and clonal relatedness.

For WGS analysis, NDM-Kpn isolates were selected according to a patient-based sampling strategy. Specifically, the first NDM-Kpn isolate per patient was included across the surveillance period. In 2025, because of the sharp increase in NDM plus OXA-48-like co-producing *K. pneumoniae*, an additional subset of clinically relevant isolates was included to better capture the clonal and genomic diversity of this emerging resistance profile. These isolates were selected prioritising invasive isolates and isolates recovered from infection-associated clinical specimens, while also ensuring representation of different hospitals and collection periods.

The yearly distribution of eligible and sequenced isolates according to carbapenemase profile is shown in Figure 2.

### 4.2. Characterisation of Bacterial Isolates and of Resistance and Virulence Determinants

Antimicrobial susceptibility testing (AST) and bacterial identification were performed using the Phoenix system (Becton Dickinson Diagnostics, San Jose, CA, USA) and the MALDI TOF Biotyper Sirius system (Bruker Daltonics, Bremen, Germany), respectively. Minimum inhibitory concentrations (MICs) for colistin were determined by broth microdilution (Liofilchem, Roseto degli Abruzzi, Italy). All results were interpreted according to the current EUCAST guidelines [38], applying a CZA MIC breakpoint of 8 mg/L.

Following phenotypic screening, carbapenemase production was assessed with an immunochromatographic test (NG-Test CARBA5, NG Biotech, Paris, France). Only *K. pneumoniae* isolates confirmed to produce NDM alone, or NDM plus OXA-48-like carbapenemases, underwent additional molecular investigations, including WGS and genomic analysis, to determine clonal relationships, resistance determinants, and virulence features.

WGS was performed on the Illumina MiSeq platform (Illumina, San Diego, CA, USA), generating paired-end reads. The WGS workflow included raw-read quality control, adapter and low-quality base trimming, de novo genome assembly, assembly-quality assessment, species confirmation, and contamination checks.

Raw-read quality was assessed using FastQC (v0.11.9) [39], and trimming of adapters and low-quality bases was performed using Trimmomatic (v0.39) [40].

De novo genome assembly was performed using Unicycler (v0.5.1) [41]. Assembly quality was evaluated using QUAST (v5.3.0) [42] and BUSCO (v5.8.2) [43], based on assembly size, number of contigs, N50, genome completeness, and evidence of possible contamination or poor-quality assemblies. Only assemblies showing quality metrics consistent with *K. pneumoniae* genomes were retained for downstream analyses.

AMR determinants were identified using ResFinder (v4.7.2) (http://www.genomicepidemiology.org, accessed on 4 June 2026), applying a minimum identity threshold of 100% and an alignment length greater than 98%.

STs were assigned using Pathogenwatch (v88) (https://pathogen.watch/en, accessed on 4 June 2026), based on the *K. pneumoniae* MLST scheme. Pathogenwatch was also used as a complementary platform for species confirmation and to support the interpretation of virulence-associated loci and capsular antigen predictions.

Kleborate (v3.0) [44,45] (https://github.com/klebgenomics/Kleborate, accessed on 4 June 2026) was used to predict virulence-associated loci and assign virulence scores (0–5), K and O antigen serotypes, and the corresponding *wzi* allele for *Klebsiella* spp. Virulence scores were assigned according to the Kleborate virulence scoring framework, based on the presence or absence of yersiniabactin, colibactin, aerobactin, and *rmp*-associated loci. Loci reported as incomplete, fragmented, or below the tool-specific confidence thresholds were not considered positive for virulence-score assignment unless confirmed by concordant evidence from the complementary analysis.

### 4.3. Analysis of Clonal Relatedness

Genomic relatedness was assessed using the WGS-based cgMLST scheme (v1.0), implemented in Ridom SeqSphere+ (Ridom GmbH, Münster, Germany), with default parameters. Comparative analyses were based on the *K. pneumoniae* sensu lato cgMLST scheme, comprising 2358 target genes [46]. Minimum spanning trees were generated for visualisation, with allele assignments referenced to the genome NC_012731. CTs were defined as groups of isolates differing by ≤15 alleles (https://www.cgmlst.org, accessed on 5 June 2026).

All raw sequencing reads were deposited in the Sequence Read Archive (SRA) under BioProject IDs PRJNA686854, PRJNA1125835, and PRJNA1193862. Specifically, 126 genomes included in the present analysis were retrieved from the previous regional surveillance study and had already been deposited under the BioProject IDs PRJNA686854 and PRJNA1125835, while the remaining genomes were newly generated in this study and deposited under the BioProject ID PRJNA1193862.

### 4.4. Statistical Analysis

Yearly proportions of NDM plus OXA-48-like co-producing *K. pneumoniae* were calculated among CZA-resistant *K. pneumoniae* isolates submitted to the regional surveillance network. Wilson 95% confidence intervals were calculated for yearly proportions. Temporal trends across the study period were assessed using the Cochran-Armitage test for trend, implemented in the DescTools package (v0.99.60) (available at https://CRAN.R-project.org/package=DescTools, accessed on 23 July 2026) [47] in R software (v4.4.2) (available at https://www.r-project.org/, accessed on 23 July 2026) [48]. A two-sided *p*-value < 0.05 was considered statistically significant.

## 5. Conclusions

In conclusion, our findings document a rapid shift towards NDM-mediated resistance among CZA-resistant *K. pneumoniae* isolates submitted to the regional surveillance network, characterized by the expansion of NDM and OXA-48-like co-producing isolates and the predominance of a widely distributed ST147 clone.

The detection of lineages combining an XDR phenotype with increased virulence further highlights an alarming epidemiological scenario.

These results support the implementation of coordinated regional genomic surveillance, timely inter-hospital data sharing, and strengthened infection prevention and control strategies to monitor the dissemination of high-risk clones and guide public health interventions.

## Figures and Tables

**Table 1:** Yearly distribution of carbapenemase profiles among CZA-resistant *Klebsiella pneumoniae* isolates submitted to the Latium regional surveillance network, 2019–2025.

Year	Nr. of Isolates	Serine β-Lactamase Nr. (%)		Metallo β-Lactamase Nr. (%)		B-Lactamase Combination Nr. (%)								
		KPC	OXA-48-like	NDM	VIM	KPC + OXA-48-like	KPC + OXA-48-like + VIM	KPC + VIM	KPC + NDM	KPC + NDM + OXA-48-like	NDM + OXA-48-like	NDM + VIM	NDM + OXA-48-like + VIM	OXA-48-like + VIM
2019	45	22 (48.9)		6 (13.3)	13 (28.9)						4 (8.9)			
2020	68	40 (58.8)	1 (1.5)	5 (7.4)	15 (22.1)			5 (7.4)			2 (2.9)			
2021	166	95 (57.2)	2 (1.2)	14 (8.4)	54 (32.5)			1 (0.6)						
2022	209	113 (54.1)	2 (1)	21 (10)	60 (28.7)			9 (4.3)	4 (1.9)					
2023	240	116 (48.3)	2 (0.8)	51 (21.3)	39 (16.3)			9 (3.8)	2 (0.8)		14 (5.8)	7 (2.9)		
2024	497	161 (32.4)	8 (1.6)	118 (23.7)	42 (8.5)			9 (1.8)	6 (1.2)	2 (0.4)	129 (26)	20 (4)		2 (0.4)
2025	1099	298 (27.1)	42 (3.8)	211 (19.2)	31 (2.8)	4 (0.36)	1 (0.09)	5 (0.46)	13 (1.18)	5 (0.46)	479 (43.6)	9 (0.8)	1 (0.09)	
Nr. (%)	2324	845 (36.4)	57 (2.5)	426 (18.3)	254 (10.9)	4 (0.17)	1 (0.04)	38 (1.7)	25 (1.1)	7 (0.3)	628 (27)	36 (1.6)	1 (0.04)	2 (0.09)

**Table 2:** Overview of genomic characteristics of different Sequence Types NDM-Kpn isolates circulating in the Latium Region.

Sequence Type	Total Nr. (%)	MBL Variants		β-Lactamase Combinations						Typing			Virulence Score ^4^			
		NDM-1	NDM-5	NDM-1+ OXA-48-like	NDM-5+ OXA-48-like	NDM-1+ KPC-3	NDM-5+ KPC-3	NDM-1+ VIM-1	NDM-1+ OXA-48-like+ KPC-3	CT ^1^	KL ^2^	H ^3^	0	1	3	4
**ST147**	**276** **(63.1%)**	59	12	163	16			26		CT-1 (166) CT-2 (44) CT-3 (30) CT-4 (12) CT-5 (5) CT-6 (4) CT-7 (3) CT-8 (2) CT-9 (2)	KL10 (172) KL51 (14) KL64 (90)	24	12	219		45
**ST11**	**88** **(20.1%)**	80	3	1		3			1	CT-10 (81) CT-19 (3) CT-21 (2)	KL105 (3) KL15 (1) KL24 (83) KL47 (1)	12	2	85		1
**ST512**	**14** **(3.2%)**	7	1			4	2			CT-12 (7) CT-13 (7)	KL107 (14)	6	2	12		
**ST1805**	**12** **(2.7%)**	12								CT-11 (12)	KL48 (12)	6	1		11	
**ST395**	**9** **(2.1%)**	9								CT-15 (4) CT-18 (3)	KL2 (8) KL39 (1)	4	1			8
**ST383**	**7** **(1.6%)**		1	4	2					CT-16 (3) CT-20 (2)	KL30 (7)	4	3		3	1
**ST15**	**5** **(1.1%)**	2		3						CT-17 (3) CT-22 (2)	KL112 (5)	2		2		3
**ST6668**	**4** **(0.9%)**	4								CT-14 (4)	KL64 (4)	2		4		
**ST14**	**2** **(0.4%)**		2							CT-23 (2)	KL2 (2)	1	2			
**ST20**	**2** **(0.4%)**	2								CT-24 (2)	KL28 (2)	1	2			
**ST307**	**2** **(0.4%)**	1				1				-	KL102 (1) KL64 (1)	2	1	1		
**ST** **unknown**	**2** **(0.4%)**				2					CT-25 (2)	KL30 (2)	1			2	
**ST8609**	**1** **(0.2%)**			1						CT-1 (1)	KL10 (1)	1		1		

Thirteen additional STs (ST16, ST17, ST23, ST25, ST29, ST39, ST200, ST234, ST1117, ST3299, ST4081, ST4853 and ST6118) were represented by single isolates. ^1^ Cluster Types (CT-1 to CT-25) were identified using cgMLST method; the number in parentheses expresses the number of isolates within the cluster. ^2^ Capsular Types (KL); the number in parentheses expresses the number of isolates. ^3^ Hospital (H): Number of different hospitals where isolates were collected; ^4^ Virulence score range from 0 to 5. The virulence scores were defined based on the presence or absence of these loci, as follows: 0 = no yersiniabactin, colibactin or aerobactin; 1 = yersiniabactin only; 2 = yersiniabactin and colibactin (or colibactin only); 3 = aerobactin without yersiniabactin or colibactin; 4 = aerobactin with yersiniabactin (no colibactin); and 5 = yersiniabactin, colibactin, and aerobactin.

## Data Availability

Raw sequencing reads have been deposited in the Sequence Read Archive (SRA) under BioProject IDs PRJNA686854, PRJNA1125835, and PRJNA1193862. Additional study data are available in an ad hoc Excel database archived at the authors’ institution (INMI “L. Spallanzani” IRCCS, Rome, Italy).

## Supplementary Materials

The following supporting information can be downloaded at: https://www.mdpi.com/article/10.3390/antibiotics15080766/s1, Figure S1: Minor clonal clusters (CT-10 to CT-25) among NDM-Kpn isolates; Table S1: Distribution of CZA-resistant CRE according to species, beta-lactamase profile, yearly proportions of NDM plus OXA-48-like co-producing *K. pneumoniae* and statistical analysis; Table S2: Yearly distribution of carbapenemase profiles among CZA-resistant *E. coli* isolates submitted to the Latium regional surveillance network, 2019–2025. Table S3: Molecular characterization of NDM-Kpn isolates; Table S4: Molecular typing and characterization of virulence genes of NDM-Kpn isolates; and Table S5: Major clonal clusters (CT-1 to CT-9) among NDM-Kpn isolates.

## Abbreviations

The following abbreviations are used in this manuscript:

CR-Kp	Carbapenem-resistant *Klebsiella pneumoniae*
MBLs	Metallo-β-lactamases
NDM	New Delhi metallo-β-lactamase
CZA	Ceftazidime-avibactam
CRE	Carbapenem-resistant *Enterobacterales*
EUCAST	European Committee on Antimicrobial Susceptibility Testing
WGS	Whole-genome sequencing
INMI	National Institute for Infectious Diseases “Lazzaro Spallanzani”
AMR	Antimicrobial resistance
ECDC	European Centre for Disease Prevention and Control
MICs	Minimum inhibitory concentrations
XDR	Extensively drug-resistant
ST	Sequence Type
CT	Cluster Type
cgMLST	Core-genome multi-locus sequence typing
KL	Capsular locus
SRA	Sequence Read Archive
CC	Clonal Complex
MDR	Multidrug-resistant
CI	Confidence Interval
AST	Antimicrobial susceptibility testing
