# Supplementary material for: Emergence and Dissemination of NDM+OXA-48-like Co-Producing Klebsiella pneumoniae in a Regional Healthcare Network: Seven-Year Surveillance from Latium, Italy
Source: Antibiotics (Basel). 2026 Aug 10;15(8):766. doi: 10.3390/antibiotics15080766 (PMC13509432; doi:10.3390/antibiotics15080766)
Supplement: Supplementary file 1 [file antibiotics-15-00766-s001.zip › Figure S1-edited.pdf]

Figure S1. Minor clonal clusters (CT-10 to CT-25) among NDM-Kpn isolates.

CT-10: 81 isolates ST11

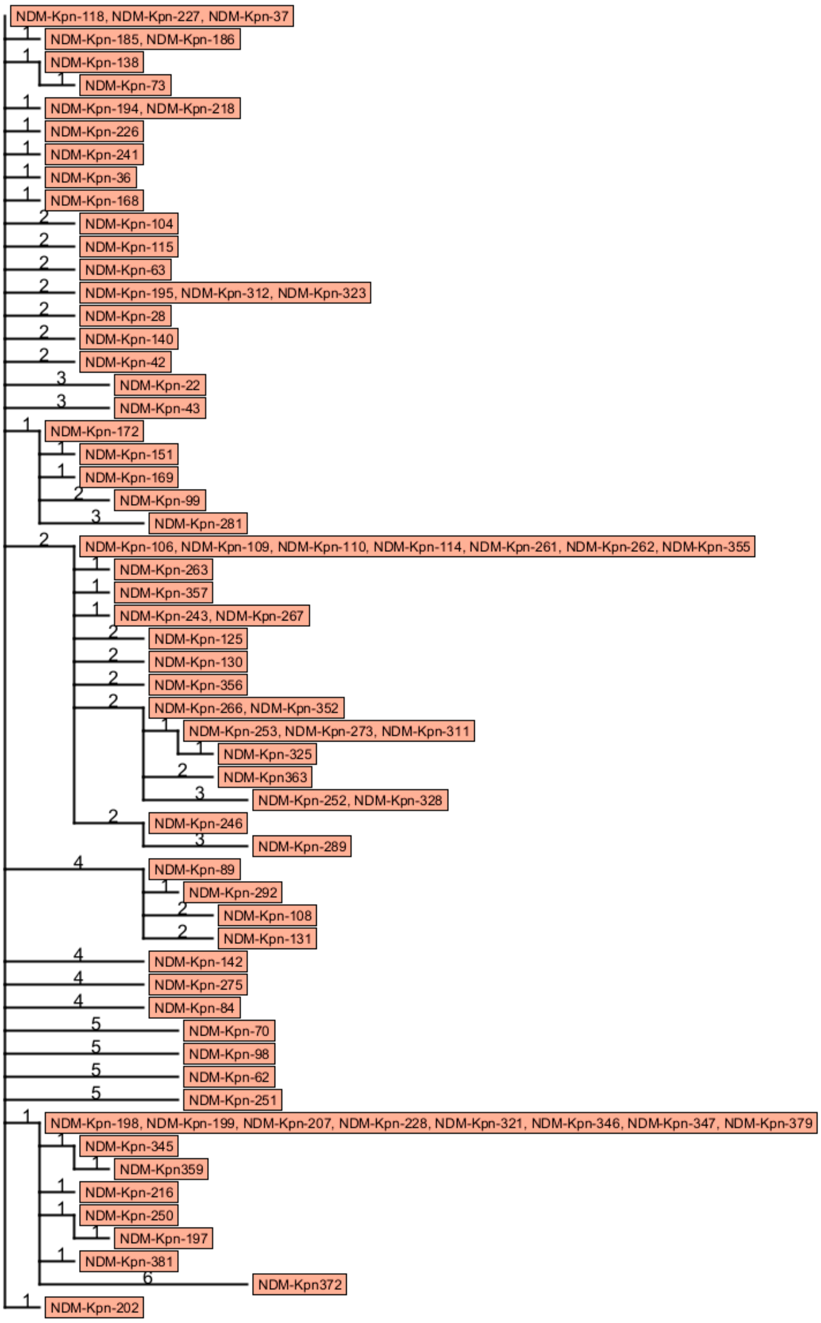

NDM-Kpn-22: *bla*$_{\text{NDM-1}}$, *bla*$_{\text{CTX-M-15}}$; virulence score: 1; H-9; 2021

NDM-Kpn-28: *bla*$_{\text{NDM-1}}$, *bla*$_{\text{CTX-M-15}}$; virulence score: 1; H-3; 2021

NDM-Kpn-36: *bla*$_{\text{NDM-1}}$, *bla*$_{\text{CTX-M-15}}$; virulence score: 1; H-23; 2022

NDM-Kpn-37: *bla*$_{\text{NDM-1}}$, *bla*$_{\text{CTX-M-15}}$; virulence score: 1; H-3; 2022

NDM-Kpn-42: *bla*$_{\text{NDM-1}}$, *bla*$_{\text{CTX-M-15}}$; virulence score: 1; H-3; 2022

NDM-Kpn-43: *bla*$_{\text{NDM-1}}$, *bla*$_{\text{CTX-M-15}}$; virulence score: 1; H-20; 2022

NDM-Kpn-62: *bla*$_{\text{NDM-1}}$, *bla*$_{\text{KPC-3}}$, *bla*$_{\text{CTX-M-15}}$; virulence score: 1; H-18; 2023

NDM-Kpn-63: $bla_{\text{NDM-1}}$, $bla_{\text{CTX-M-15}}$; virulence score: 1; H-3; 2023

NDM-Kpn-70: $bla_{\text{NDM-1}}$, $bla_{\text{CTX-M-15}}$; virulence score: 1; H-17; 2023

NDM-Kpn-73: $bla_{\text{NDM-1}}$, $bla_{\text{CTX-M-15}}$; virulence score: 1; H-3; 2023

NDM-Kpn-84: $bla_{\text{NDM-1}}$, $bla_{\text{CTX-M-15}}$; virulence score: 1; H-2; 2023

NDM-Kpn-89: $bla_{\text{NDM-1}}$; virulence score: 1; H-3; 2023

NDM-Kpn-98: $bla_{\text{NDM-1}}$, $bla_{\text{CTX-M-15}}$; virulence score: 1; H-2; 2023

NDM-Kpn-99: $bla_{\text{NDM-1}}$, $bla_{\text{CTX-M-15}}$; virulence score: 1; H-3; 2023

NDM-Kpn-104: $bla_{\text{NDM-1}}$, $bla_{\text{CTX-M-15}}$; virulence score: 1; H-1; 2023

NDM-Kpn-106: $bla_{\text{NDM-1}}$, $bla_{\text{CTX-M-15}}$; virulence score: 1; H-1; 2023

NDM-Kpn-108: $bla_{\text{NDM-1}}$, $bla_{\text{CTX-M-15}}$; virulence score: 1; H-1; 2023

NDM-Kpn-109: $bla_{\text{NDM-1}}$, $bla_{\text{CTX-M-15}}$; virulence score: 1; H-1; 2023

NDM-Kpn-110: $bla_{\text{NDM-1}}$, $bla_{\text{CTX-M-15}}$; virulence score: 1; H-1; 2023

NDM-Kpn-114: $bla_{\text{NDM-1}}$, $bla_{\text{CTX-M-15}}$; virulence score: 1; H-1; 2023

NDM-Kpn-115: $bla_{\text{NDM-1}}$, $bla_{\text{CTX-M-15}}$; virulence score: 1; H-1; 2023

NDM-Kpn-118: $bla_{\text{NDM-1}}$, $bla_{\text{CTX-M-15}}$; virulence score: 1; H-3; 2023

NDM-Kpn-125: $bla_{\text{NDM-1}}$, $bla_{\text{CTX-M-15}}$; virulence score: 1; H-1; 2023

NDM-Kpn-130: $bla_{\text{NDM-1}}$, $bla_{\text{KPC-3}}$, $bla_{\text{CTX-M-15}}$; virulence score: 1; H-1; 2024

NDM-Kpn-131: $bla_{\text{NDM-1}}$; virulence score: 1; H-1; 2024

NDM-Kpn-138: $bla_{\text{NDM-1}}$, $bla_{\text{CTX-M-15}}$; virulence score: 1; H-3; 2024

NDM-Kpn-140: $bla_{\text{NDM-1}}$, $bla_{\text{CTX-M-15}}$; virulence score: 1; H-1; 2024

NDM-Kpn-142: $bla_{\text{NDM-1}}$, $bla_{\text{CTX-M-15}}$; virulence score: 1; H-3; 2024

NDM-Kpn-151: $bla_{\text{NDM-1}}$, $bla_{\text{CTX-M-15}}$; virulence score: 1; H-3; 2024

NDM-Kpn-168: $bla_{\text{NDM-1}}$, $bla_{\text{CTX-M-15}}$; virulence score: 1; H-2; 2024

NDM-Kpn-169: $bla_{\text{NDM-1}}$, $bla_{\text{CTX-M-15}}$; virulence score: 1; H-2; 2024

NDM-Kpn-172: $bla_{\text{NDM-1}}$, $bla_{\text{CTX-M-15}}$; virulence score: 1; H-2; 2024

NDM-Kpn-185: $bla_{\text{NDM-1}}$, $bla_{\text{CTX-M-15}}$; virulence score: 1; H-9; 2024

NDM-Kpn-186: $bla_{\text{NDM-1}}$, $bla_{\text{CTX-M-15}}$; virulence score: 1; H-9; 2024

NDM-Kpn-194: $bla_{\text{NDM-1}}$, $bla_{\text{CTX-M-15}}$; virulence score: 1; H-3; 2024

NDM-Kpn-195: $bla_{\text{NDM-1}}$, $bla_{\text{CTX-M-15}}$; virulence score: 1; H-14; 2024

NDM-Kpn-197: $bla_{\text{NDM-1}}$, $bla_{\text{CTX-M-15}}$; virulence score: 1; H-2; 2024

NDM-Kpn-198: $bla_{\text{NDM-1}}$, $bla_{\text{CTX-M-15}}$; virulence score: 1; H-2; 2024

NDM-Kpn-199: $bla_{\text{NDM-1}}$, $bla_{\text{CTX-M-15}}$; virulence score: 1; H-2; 2024

NDM-Kpn-202: $bla_{\text{NDM-1}}$, $bla_{\text{CTX-M-15}}$; virulence score: 1; H-9; 2024

NDM-Kpn-207: $bla_{\text{NDM-1}}$, $bla_{\text{CTX-M-15}}$; virulence score: 1; H-2; 2024

NDM-Kpn-216: $bla_{\text{NDM-1}}$, $bla_{\text{CTX-M-15}}$; virulence score: 1; H-2; 2024

NDM-Kpn-218: $bla_{\text{NDM-1}}$, $bla_{\text{OXA-48}}$, $bla_{\text{CTX-M-15}}$; virulence score: 1; H-3; 2024

NDM-Kpn-226: $bla_{\text{NDM-1}}$, $bla_{\text{CTX-M-15}}$; virulence score: 1; H-10; 2024

NDM-Kpn-227: *bla*$_{\text{NDM-1}}$, *bla*$_{\text{CTX-M-15}}$; virulence score: 1; H-10; 2024

NDM-Kpn-228: *bla*$_{\text{NDM-1}}$, *bla*$_{\text{CTX-M-15}}$; virulence score: 1; H-2; 2024

NDM-Kpn-241: *bla*$_{\text{NDM-1}}$, *bla*$_{\text{CTX-M-15}}$; virulence score: 1; H-1; 2024

NDM-Kpn-243: *bla*$_{\text{NDM-1}}$, *bla*$_{\text{CTX-M-15}}$; virulence score: 1; H-1; 2024

NDM-Kpn-246: *bla*$_{\text{NDM-1}}$, *bla*$_{\text{KPC-3}}$, *bla*$_{\text{CTX-M-15}}$; virulence score: 1; H-1; 2024

NDM-Kpn-250: *bla*$_{\text{NDM-1}}$, *bla*$_{\text{CTX-M-15}}$; virulence score: 1; H-1; 2024

NDM-Kpn-251: *bla*$_{\text{NDM-1}}$, *bla*$_{\text{CTX-M-15}}$; virulence score: 1; H-1; 2024

NDM-Kpn-252: *bla*$_{\text{NDM-1}}$, *bla*$_{\text{CTX-M-15}}$; virulence score: 1; H-1; 2024

NDM-Kpn-253: *bla*$_{\text{NDM-1}}$, *bla*$_{\text{CTX-M-15}}$; virulence score: 1; H-1; 2024

NDM-Kpn-261: *bla*$_{\text{NDM-1}}$, *bla*$_{\text{CTX-M-15}}$; virulence score: 1; H-1; 2024

NDM-Kpn-262: *bla*$_{\text{NDM-1}}$, *bla*$_{\text{CTX-M-15}}$; virulence score: 1; H-1; 2024

NDM-Kpn-263: *bla*$_{\text{NDM-1}}$, *bla*$_{\text{CTX-M-15}}$; virulence score: 1; H-1; 2024

NDM-Kpn-266: *bla*$_{\text{NDM-1}}$, *bla*$_{\text{CTX-M-15}}$, *bla*$_{\text{CMY-4}}$; virulence score: 0; H-1; 2024

NDM-Kpn-267: *bla*$_{\text{NDM-1}}$, *bla*$_{\text{CTX-M-15}}$; virulence score: 1; H-1; 2024

NDM-Kpn-273: *bla*$_{\text{NDM-1}}$, *bla*$_{\text{CTX-M-15}}$; virulence score: 1; H-3; 2024

NDM-Kpn-275: *bla*$_{\text{NDM-1}}$, *bla*$_{\text{CTX-M-15}}$; virulence score: 1; H-10; 2024

NDM-Kpn-281: *bla*$_{\text{NDM-1}}$, *bla*$_{\text{CTX-M-15}}$; virulence score: 1; H-14; 2024

NDM-Kpn-289: *bla*$_{\text{NDM-1}}$, *bla*$_{\text{CTX-M-15}}$; virulence score: 1; H-1; 2024

NDM-Kpn-292: *bla*$_{\text{NDM-1}}$, *bla*$_{\text{CTX-M-15}}$; virulence score: 1; H-1; 2024

NDM-Kpn-311: *bla*$_{\text{NDM-1}}$, *bla*$_{\text{CTX-M-15}}$; virulence score: 1; H-3; 2024

NDM-Kpn-312: *bla*$_{\text{NDM-1}}$, *bla*$_{\text{CTX-M-15}}$, *bla*$_{\text{CMY-4}}$; virulence score: 1; H-3; 2024

NDM-Kpn-321: *bla*$_{\text{NDM-1}}$, *bla*$_{\text{CTX-M-15}}$; virulence score: 1; H-2; 2024

NDM-Kpn-323: *bla*$_{\text{NDM-1}}$, *bla*$_{\text{CTX-M-15}}$; virulence score: 1; H-1; 2024

NDM-Kpn-325: *bla*$_{\text{NDM-1}}$, *bla*$_{\text{CTX-M-15}}$; virulence score: 1; H-1; 2024

NDM-Kpn-328: *bla*$_{\text{NDM-1}}$, *bla*$_{\text{CTX-M-15}}$; virulence score: 1; H-1; 2024

NDM-Kpn-345: *bla*$_{\text{NDM-1}}$, *bla*$_{\text{CTX-M-15}}$; virulence score: 1; H-2; 2024

NDM-Kpn-346: *bla*$_{\text{NDM-1}}$, *bla*$_{\text{CTX-M-15}}$; virulence score: 1; H-2; 2024

NDM-Kpn-347: *bla*$_{\text{NDM-1}}$, *bla*$_{\text{CTX-M-15}}$; virulence score: 1; H-2; 2024

NDM-Kpn-352: *bla*$_{\text{NDM-1}}$, *bla*$_{\text{KPC-3}}$, *bla*$_{\text{CTX-M-15}}$; virulence score: 1; H-1; 2024

NDM-Kpn-355: *bla*$_{\text{NDM-1}}$, *bla*$_{\text{CTX-M-15}}$; virulence score: 1; H-1; 2024

NDM-Kpn-356: *bla*$_{\text{NDM-1}}$; virulence score: 1; H-1; 2024

NDM-Kpn-357: *bla*$_{\text{NDM-1}}$, *bla*$_{\text{CTX-M-15}}$; virulence score: 1; H-1; 2024

NDM-Kpn-359: *bla*$_{\text{NDM-1}}$, *bla*$_{\text{CTX-M-15}}$; virulence score: 1; H-2; 2024

NDM-Kpn-363: *bla*$_{\text{NDM-1}}$, *bla*$_{\text{CTX-M-15}}$; virulence score: 0; H-1; 2024

NDM-Kpn-372: *bla*$_{\text{NDM-1}}$, *bla*$_{\text{CTX-M-15}}$; virulence score: 1; H-1; 2024

NDM-Kpn-379: *bla*$_{\text{NDM-1}}$, *bla*$_{\text{CTX-M-15}}$; virulence score: 1; H-2; 2024

NDM-Kpn-381: *bla*$_{\text{NDM-1}}$, *bla*$_{\text{CTX-M-15}}$; virulence score: 1; H-2; 2024

CT-11: 12 isolates ST1805

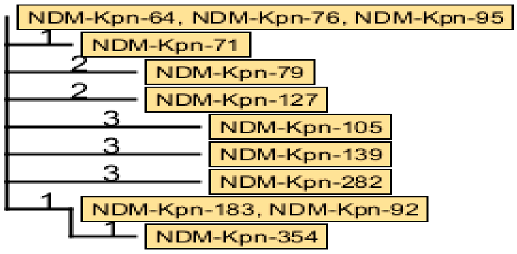

NDM-Kpn-64: $\textit{bla}_{\text{NDM-1}}$; virulence score: 3; H-3; 2023

NDM-Kpn-71: $\textit{bla}_{\text{NDM-1}}$; virulence score: 3; H-27; 2023

NDM-Kpn-76: $\textit{bla}_{\text{NDM-1}}$; virulence score: 3; H-3; 2023

NDM-Kpn-79: $\textit{bla}_{\text{NDM-1}}$; virulence score: 3; H-3; 2023

NDM-Kpn-92: $\textit{bla}_{\text{NDM-1}}$; virulence score: 3; H-8; 2023

NDM-Kpn-95: $\textit{bla}_{\text{NDM-1}}$; virulence score: 3; H-8; 2023

NDM-Kpn-105: $\textit{bla}_{\text{NDM-1}}$; virulence score: 0; H-1; 2023

NDM-Kpn-127: $\textit{bla}_{\text{NDM-1}}$, $\textit{bla}_{\text{CTX-M-15}}$; virulence score: 3; H-20; 2024

NDM-Kpn-139: $\textit{bla}_{\text{NDM-1}}$; virulence score: 3; H-1; 2024

NDM-Kpn-183: $\textit{bla}_{\text{NDM-1}}$; virulence score: 3; H-1; 2024

NDM-Kpn-282: $\textit{bla}_{\text{NDM-1}}$; virulence score: 3; H-5; 2024

NDM-Kpn-354: $\textit{bla}_{\text{NDM-1}}$; virulence score: 3; H-1; 2024

CT-12: 7 isolates ST512

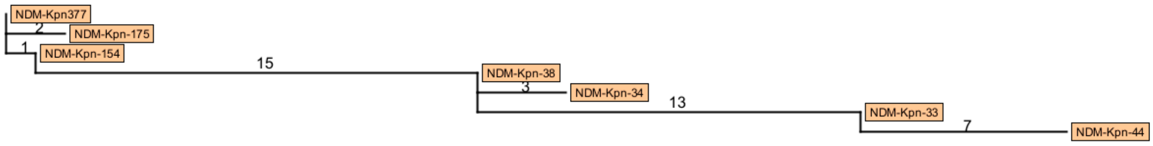

NDM-Kpn-33: $\textit{bla}_{\text{NDM-1}}$, $\textit{bla}_{\text{KPC-3}}$, $\textit{bla}_{\text{CMY-6}}$; virulence score: 0; H-3; 2022

NDM-Kpn-34: $\textit{bla}_{\text{NDM-1}}$, $\textit{bla}_{\text{KPC-3}}$, $\textit{bla}_{\text{CMY-6}}$; virulence score: 1; H-19; 2022

NDM-Kpn-38: $\textit{bla}_{\text{NDM-1}}$, $\textit{bla}_{\text{KPC-3}}$; virulence score: 1; H-19; 2022

NDM-Kpn-44: $\textit{bla}_{\text{NDM-1}}$, $\textit{bla}_{\text{KPC-3}}$, $\textit{bla}_{\text{CMY-6}}$; virulence score: 0; H-19; 2022

NDM-Kpn-154: $\textit{bla}_{\text{NDM-5}}$, $\textit{bla}_{\text{KPC-3}}$; virulence score: 1; H-3; 2024

NDM-Kpn-175: $\textit{bla}_{\text{NDM-5}}$, $\textit{bla}_{\text{KPC-3}}$; virulence score: 1; H-3; 2024

NDM-Kpn-377: $\textit{bla}_{\text{NDM-5}}$; virulence score: 1; H-12; 2024

CT-13: 7 isolates ST512

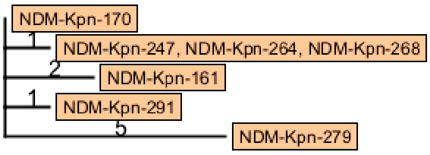

NDM-Kpn-161: *bla*$_{NDM-1}$, *bla*$_{CTX-M-15}$; virulence score: 1; H-26; 2024

NDM-Kpn-170: *bla*$_{NDM-1}$, *bla*$_{CTX-M-15}$; virulence score: 1; H-2; 2024

NDM-Kpn-247: *bla*$_{NDM-1}$, *bla*$_{CTX-M-15}$; virulence score: 1; H-1; 2024

NDM-Kpn-264: *bla*$_{NDM-1}$, *bla*$_{CTX-M-15}$; virulence score: 1; H-1; 2024

NDM-Kpn-268: *bla*$_{NDM-1}$, *bla*$_{CTX-M-15}$; virulence score: 1; H-1; 2024

NDM-Kpn-279: *bla*$_{NDM-1}$, *bla*$_{CTX-M-15}$; virulence score: 1; H-3; 2024

NDM-Kpn-291: *bla*$_{NDM-1}$, *bla*$_{CTX-M-15}$; virulence score: 1; H-1; 2024

CT-14: 4 isolates ST6668

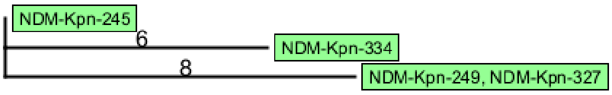

NDM-Kpn-245: *bla*$_{NDM-1}$, *bla*$_{CTX-M-15}$; virulence score: 1; H-1; 2024

NDM-Kpn-249: *bla*$_{NDM-1}$, *bla*$_{CTX-M-15}$; virulence score: 1; H-1; 2024

NDM-Kpn-327: *bla*$_{NDM-1}$, *bla*$_{CTX-M-15}$; virulence score: 1; H-1; 2024

NDM-Kpn-334: *bla*$_{NDM-1}$, *bla*$_{CTX-M-15}$; virulence score: 1; H-10; 2024

CT-15: 4 isolates ST395

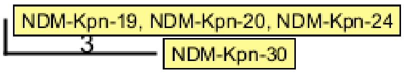

NDM-Kpn-19: *bla*$_{NDM-1}$, *bla*$_{CTX-M-15}$; virulence score: 4; H-2; 2021

NDM-Kpn-20: *bla*$_{NDM-1}$, *bla*$_{CTX-M-15}$; virulence score: 4; H-2; 2021

NDM-Kpn-24: *bla*$_{NDM-1}$, *bla*$_{CTX-M-15}$; virulence score: 4; H-5; 2021

NDM-Kpn-30: *bla*$_{NDM-1}$, *bla*$_{CTX-M-15}$; virulence score: 4; H-2; 2021

CT-16: 3 isolates ST383

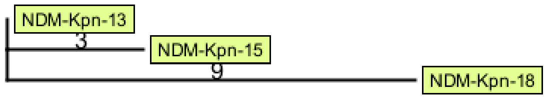

NDM-Kpn-13: *bla*$_{\text{NDM-1}}$, *bla*$_{\text{OXA-48}}$, *bla*$_{\text{CTX-M-14b}}$; virulence score: 0; H-15; 2020

NDM-Kpn-15: *bla*$_{\text{NDM-1}}$, *bla*$_{\text{OXA-48}}$, *bla*$_{\text{CTX-M-14b}}$; virulence score: 0; H-15; 2020

NDM-Kpn-18: *bla*$_{\text{NDM-1}}$, *bla*$_{\text{OXA-48}}$, *bla*$_{\text{CTX-M-15}}$; virulence score: 0; H-5; 2020

CT-17: 3 isolates ST15

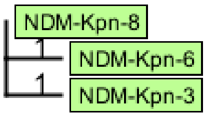

NDM-Kpn-3: *bla*$_{\text{NDM-1}}$, *bla*$_{\text{OXA-48}}$, *bla*$_{\text{CTX-M-15}}$, *bla*$_{\text{CMY-6}}$; virulence score: 4; H-13; 2019

NDM-Kpn-6: *bla*$_{\text{NDM-1}}$, *bla*$_{\text{OXA-48}}$, *bla*$_{\text{CTX-M-15}}$, *bla*$_{\text{CMY-6}}$; virulence score: 4; H-13; 2019

NDM-Kpn-8: *bla*$_{\text{NDM-1}}$, *bla*$_{\text{OXA-48}}$, *bla*$_{\text{CTX-M-15}}$, *bla*$_{\text{CMY-6}}$; virulence score: 4; H-13; 2019

CT-18: 3 isolates ST395

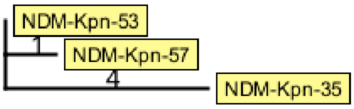

NDM-Kpn-35: *bla*$_{\text{NDM-1}}$, *bla*$_{\text{CTX-M-15}}$; virulence score: 4; H-3; 2022

NDM-Kpn-53: *bla*$_{\text{NDM-1}}$, *bla*$_{\text{CTX-M-15}}$; virulence score: 4; H-3; 2022

NDM-Kpn-57: *bla*$_{\text{NDM-1}}$, *bla*$_{\text{CTX-M-15}}$; virulence score: 4; H-3; 2023

CT-19: 3 isolates ST11

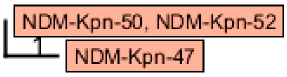

NDM-Kpn-47: *bla*$_{\text{NDM-5}}$, *bla*$_{\text{DHA-1}}$; virulence score: 1; H-2; 2022

NDM-Kpn-50: *bla*$_{\text{NDM-5}}$, *bla*$_{\text{DHA-1}}$; virulence score: 1; H-2; 2022

NDM-Kpn-52: *bla*$_{\text{NDM-5}}$, *bla*$_{\text{DHA-1}}$; virulence score: 1; H-2; 2022

CT-20: 2 isolates ST383

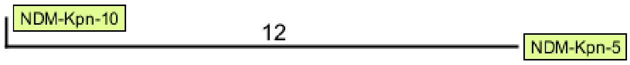

NDM-Kpn-5: *bla*$_{\text{NDM-5}}$, *bla*$_{\text{CTX-M-15}}$; virulence score: 3; H-13; 2019

NDM-Kpn-10: *bla*$_{\text{NDM-1}}$, *bla*$_{\text{OXA-48}}$, *bla*$_{\text{CTX-M-15}}$, *bla*$_{\text{CMY-6}}$; virulence score: 3; H-13; 2019

CT-21: 2 isolates ST11

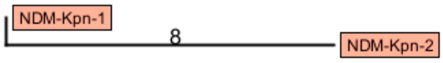

NDM-Kpn-1: *bla*$_{\text{NDM-1}}$; virulence score: 1; H-7; 2019

NDM-Kpn-2: *bla*$_{\text{NDM-1}}$; virulence score: 1; H-3; 2019

CT-22: 2 isolates ST15

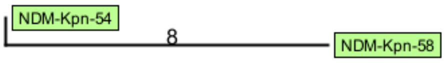

NDM-Kpn-54: *bla*$_{\text{NDM-1}}$, *bla*$_{\text{CTX-M-15}}$, *bla*$_{\text{CMY-6}}$; virulence score: 1; H-3; 2022

NDM-Kpn-58: *bla*$_{\text{NDM-1}}$, *bla*$_{\text{CTX-M-15}}$, *bla*$_{\text{CMY-6}}$; virulence score: 1; H-3; 2023

CT-23: 2 isolates ST14

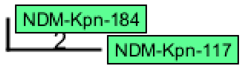

NDM-Kpn-117: *bla*$_{\text{NDM-5}}$; virulence score: 0; H-7; 2023

NDM-Kpn-184: *bla*$_{\text{NDM-5}}$; virulence score: 0; H-7; 2024

CT-24: 2 isolates ST20

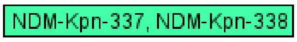

NDM-Kpn-337: *bla*$_{\text{NDM-1}}$; virulence score: 0; H-3; 2024

NDM-Kpn-338: *bla*$_{\text{NDM-1}}$; virulence score: 0; H-3; 2024

CT-25: 2 isolates ST unkown

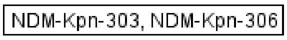

NDM-Kpn-303: *bla*$_{\text{NDM-5}}$, *bla*$_{\text{OXA-48}}$, *bla*$_{\text{CTX-M-15}}$; virulence score: 3; H-8; 2024

NDM-Kpn-306: *bla*$_{\text{NDM-5}}$, *bla*$_{\text{OXA-48}}$, *bla*$_{\text{CTX-M-15}}$; virulence score: 3; H-8; 2024
