# Supplementary material for: Emergence and Dissemination of NDM+OXA-48-like Co-Producing Klebsiella pneumoniae in a Regional Healthcare Network: Seven-Year Surveillance from Latium, Italy
Source: Antibiotics (Basel). 2026 Aug 10;15(8):766. doi: 10.3390/antibiotics15080766 (PMC13509432; doi:10.3390/antibiotics15080766)
Supplement: Supplementary file 1 [file antibiotics-15-00766-s001.zip › Table S1-edited.pdf]

Table S1. Distribution of CZA-resistant CRE according to species, beta-lactamase profile, yearly proportions of NDM plus OXA-48-like co-producing *K. pneumoniae* and statistical analysis.

Panel A. Proportions of CZA-resistant CRE and beta-lactamase genes (years 2019 to 2025).

Microorganism	Serine β-lactamase		Metallo β-lactamase		B-lactamase combination									Nr. (%)
	KPC	OXA-48	NDM	VIM	NDM+ OXA-48	NDM+VIM	NDM+KPC	NDM+ OXA-48+KPC	NDM+ OXA-48+VIM	OXA-48+KPC	KPC+VIM	KPC+ OXA-48+VIM	OXA-48+VIM	
K. pneumoniae	845	57	426	254	628	36	25	7	1	4	38	1	2	2324 (84,45%)
K. oxytoca	1	1		10							1			13 (0,47%)
K. variicola	1			7										8 (0,29%)
K. aerogenes				4	1					1				6 (0,22%)
K. ozaenae	1										2			3 (0,11%)
E. coli	5		205	38	9	2	2				4			265 (9,63%)
E. cloacae	2		4	31			1				1			39 (1,42%)
E. hormaechei			13	10										23 (0,84%)
E. bugandensis				2										2 (0,07%)
E. kobei				1										1 (0,04%)
C. freundii	2	1		20							3	1		27 (0,98%)
C. koseri			1	2										3 (0,11%)
P. mirabilis	1		23	1										25 (0,91%)
P. rettgeri						4								4 (0,15%)
P. stuartii			4											4 (0,15%)
R. ornithinolytica			1	2										3 (0,11%)
R. planticola				1										1 (0,04%)
M. morganii			1											1 (0,04%)
**Nr. (%)**	**858**	**59**	**678**	**383**	**638**	**42**	**28**	**7**	**1**	**5**	**49**	**2**	**2**	**2752 (100)**
	**917 (33,3)**		**1061 (38,5)**		**774 (28,1)**									

Panel B. Yearly proportions and statistical analysis of NDM plus OXA-48-like co-producing *K. pneumoniae* among CZA-resistant *K. pneumoniae* isolates submitted to the regional surveillance network.

Year	CZA-resistant *K. pneumoniae*	*K. pneumoniae* co-producing NDM+OXA-48-like	Proportion, %	Wilson 95% CI
2019	45	4	8.9	3.5–20.7
2020	68	2	2.9	0.8–10.1
2021	166	0	0.0	0.0–2.3
2022	209	0	0.0	0.0–1.8
2023	241	14	5.8	3.5–9.5
2024	496	129	26.0	22.3–30.0
2025	1099	479	43.6	40.7–46.5
**Total of isolates**	2324	628	27.0	25.3–28.9

Note that proportions were calculated using the total number of CZA-resistant *K. pneumoniae* isolates collected each year as the denominator. Wilson 95% confidence intervals (CI) are reported for each yearly proportion. The temporal trend was assessed using the Cochran–Armitage test for trend.
