# Supplementary material for: Emergence and Dissemination of NDM+OXA-48-like Co-Producing Klebsiella pneumoniae in a Regional Healthcare Network: Seven-Year Surveillance from Latium, Italy
Source: Antibiotics (Basel). 2026 Aug 10;15(8):766. doi: 10.3390/antibiotics15080766 (PMC13509432; doi:10.3390/antibiotics15080766)
Supplement: Supplementary file 1 [file antibiotics-15-00766-s001.zip › Table S2-edited.pdf]

Table S2. Yearly distribution of carbapenemase profiles among CZA-resistant *E. coli* isolates submitted to the Latium regional surveillance network, 2019–2025.

Year	Nr. of isolates	Serine β-lactamase Nr. (%)		Metallo β-lactamase Nr. (%)		B-lactamase combination Nr. (%)							
		KPC	OXA-48-like	NDM	VIM	KPC+ OXA-48	KPC+ OXA-48-like + VIM	KPC + VIM	KPC + NDM	KPC + NDM + OXA-48-like	NDM + OXA-48-like	NDM + OXA-48-like + VIM	OXA-48-like + VIM
2019	5			3 (60)							2 (40)		
2020	16			10 (62,5)	3 (18,8)			2 (12,5)			1 (6,3)		
2021	7			5 (71,4)	2 (28,6)								
2022	17			5 (29,4)	10 (58,8)			1 (5,9)			1 (5,9)		
2023	68	3 (4,4)		56 (82,4)	5 (7,4)				2 (2,9)		1 (1,5)	1 (1,5)	
2024	57			46 (80,7)	10 (17,5)			1 (1,8)					
2025	95	2 (2,1)		80 (84,2)	8 (8,4)						4 (4,2)	1 (1,1)	
**Nr. (%)**	**265**	**5 (1,9)**		**205 (77,4)**	**38 (14,3)**			**4 (1,5)**	**2 (0,8)**		**9 (3,4)**	**2 (0,8)**	
