# Supplementary material for: Emergence and Dissemination of NDM+OXA-48-like Co-Producing Klebsiella pneumoniae in a Regional Healthcare Network: Seven-Year Surveillance from Latium, Italy
Source: Antibiotics (Basel). 2026 Aug 10;15(8):766. doi: 10.3390/antibiotics15080766 (PMC13509432; doi:10.3390/antibiotics15080766)
Supplement: Supplementary file 1 [file antibiotics-15-00766-s001.zip › Table S3-edited.pdf]

Table S3. Molecular characterization of NDM-Kpn isolates.

**Strain**	**Resistance determinants**	
	**β-lactamase genes**	**Additional resistance genes**
NDM-Kpn-1	***bla***$_{\textbf{NDM-1}}$, *bla*$_{\text{SHV-107}}$	*aac(6)-Ib, fosA3, oqxA, oqxB, dfrA14*
NDM-Kpn-2	***bla***$_{\textbf{NDM-1}}$, *bla*$_{\text{SHV-11}}$	*fosA3, oqxA, oqxB, dfrA14*
NDM-Kpn-3	***bla***$_{\textbf{NDM-1}}$, ***bla***$_{\textbf{OXA-48}}$, *bla*$_{\text{CTX-M-15}}$, *bla*$_{\text{CMY-6}}$, *bla*$_{\text{SHV-28}}$	*aac(6)-Ib, rmtC, fosA3, oqxA, oqxB, sul1, sul2, dfrA14*
NDM-Kpn-4	***bla***$_{\textbf{NDM-1}}$, *bla*$_{\text{CTX-M-15}}$, *bla*$_{\text{OXA-1}}$, *bla*$_{\text{OXA-9}}$, *bla*$_{\text{SHV-81}}$, *bla*$_{\text{TEM-1}}$	*aph(3')-VI, aac(6')-Ib, aadA1, armA, aac(6')-Ib-cr, fosA3, fosA5, msr(E), mph(A), mph(E), catB3, qnrS1, oqxA, oqxB, ARR-3, sul1, sul2, dfrA5*
NDM-Kpn-5	***bla***$_{\textbf{NDM-5}}$, *bla*$_{\text{CTX-M-15}}$, *bla*$_{\text{OXA-9}}$, *bla*$_{\text{SHV-26}}$, *bla*$_{\text{TEM-1}}$	*aph(3')-VI, aac(6')-Ib, aadA1, aph(3')-Ia, armA, fosA3, fosA5, msr(E), mph(A), mph(E), catA1, qnrS1, oqxA, oqxB, sul1, sul2, tet(A), dfrA5*
NDM-Kpn-6	***bla***$_{\textbf{NDM-1}}$, ***bla***$_{\textbf{OXA-48}}$, *bla*$_{\text{CTX-M-15}}$, *bla*$_{\text{CMY-6}}$, *bla*$_{\text{SHV-55}}$	*aac(6')-Ib, rmtC ,fosA3, oqxA, oqxB, sul1, sul2, dfrA14*
NDM-Kpn-7	***bla***$_{\textbf{NDM-1}}$, *bla*$_{\text{CTX-M-15}}$, *bla*$_{\text{OXA-1}}$, *bla*$_{\text{OXA-10}}$, *bla*$_{\text{SHV-28}}$, *bla*$_{\text{TEM-1}}$	*aph(6)-Id, aac(6')-II, aph(3')-VI, aac(3')-II, aac(6')-Ib-cr, aph(3'')-Ib, fosA3, catB3, qnrB1, qnrS1, oqxA, oqxB, sul1, sul2, tet(A), dfrA14*
NDM-Kpn-8	***bla***$_{\textbf{NDM-1}}$, ***bla***$_{\textbf{OXA-48}}$, *bla*$_{\text{CTX-M-15}}$, *bla*$_{\text{CMY-6}}$, *bla*$_{\text{SHV-106}}$	*aac(6')-Ib, rmtC, fosA3, oqxA, oqxB, sul1, sul2*
NDM-Kpn-9	***bla***$_{\textbf{NDM-1}}$, ***bla***$_{\textbf{OXA-48}}$, *bla*$_{\text{CTX-M-14b}}$, *bla*$_{\text{CMY-6}}$, *bla*$_{\text{SHV-148}}$, *bla*$_{\text{TEM-1}}$	*aph(6)-Id, aac(3')-IIa, aac(6')-Ib, aph(3')-VI, aph(3'')-Ib, rmtC, mph(A), fosA3, catA1, oqxA, oqxB, sul1, tet(A)*
NDM-Kpn-10	***bla***$_{\textbf{NDM-1}}$, ***bla***$_{\textbf{OXA-48}}$, *bla*$_{\text{CMY-6}}$, *bla*$_{\text{CTX-M-15}}$, *bla*$_{\text{CTX-M-14b}}$, *bla*$_{\text{SHV-148}}$, *bla*$_{\text{OXA-9}}$, *bla*$_{\text{TEM-1}}$	*aph(6)-Id, aac(6')-Ib, aph(3')-VI, rmtC, mph(A), aac(6')-Ib-cr, aph(3'')-Ib, fosA3, catA1, qnrS1, oqxA, oqxB, sul1, sul2, tet(A)*
NDM-Kpn-11	***bla***$_{\textbf{NDM-1}}$, *bla*$_{\text{CTX-M-15}}$, *bla*$_{\text{SCO-1}}$, *bla*$_{\text{SHV-155}}$, *bla*$_{\text{OXA-1}}$, *bla*$_{\text{TEM-1}}$	*aph(6)-Id, aac(3')-IIa, addA1, aac(6')-Ib-cr, fosA3, catB3, qnrB1, oqxA, oqxB, sul1, sul2, tet(A), dfrA15*
NDM-Kpn-12	***bla***$_{\textbf{NDM-1}}$, *bla*$_{\text{CTX-M-15}}$, *bla*$_{\text{OXA-1}}$, *bla*$_{\text{OXA-9}}$, *bla*$_{\text{SHV-11}}$, *bla*$_{\text{TEM-1}}$	*aac(6')-Ib, aph(3')-VI, aph(3')-Ia aac(6')-Ib-cr, aadA1, armA, msr(E), mph(A), mph(E), fosA5, catB3, qnrS1, oqxA, oqxB, ARR-3, sul1, sul2, dfrA5*
NDM-Kpn-13	***bla***$_{\textbf{NDM-1}}$, ***bla***$_{\textbf{OXA-48}}$, *bla*$_{\text{CTX-M-14b}}$, *bla*$_{\text{SHV-148}}$	*aph(6)-Id, aph(3'')-Ib, rmtF, mph(A), fosA3, fosA5, catA1, qnrB1, oqxA, oqxB, AAR-2, tet(A)*
NDM-Kpn-14	***bla***$_{\textbf{NDM-1}}$, *bla*$_{\text{CTX-M-15}}$, *bla*$_{\text{OXA-1}}$, *bla*$_{\text{SHV-11}}$, *bla*$_{\text{TEM-1}}$	*aadA1, aac(6')-Ib-cr, fosA3, fosA5, catB3, qnrB1, oqxA, oqxB, sul1, dfrA15*
NDM-Kpn-15	***bla***$_{\textbf{NDM-1}}$, ***bla***$_{\textbf{OXA-48}}$, *bla*$_{\text{CTX-M-14b}}$, *bla*$_{\text{SHV-148}}$	*aph(6)-Id, aph(3'')-Ib, rmtF, mph(A), fosA3, fosA5, catA1, qnrB1, oqxA, oqxB, AAR-2, tet(A)*
NDM-Kpn-16	***bla***$_{\textbf{NDM-1}}$, *bla*$_{\text{CTX-M-15}}$, *bla*$_{\text{OXA-1}}$, *bla*$_{\text{SHV-81}}$, *bla*$_{\text{TEM-1}}$	*aadA1, aac(6')-Ib-cr, fosA3, fosA5, catB3, qnrB1, oqxA, oqxB, sul1, dfrA1*
NDM-Kpn-17	***bla***$_{\textbf{NDM-1}}$, *bla*$_{\text{OXA-10}}$, *bla*$_{\text{SHV-2}}$, *bla*$_{\text{SHV-187}}$, *bla*$_{\text{TEM-1}}$	*aac(6')-II, aph(3')-VI, acc(3)-II, aadA16, aac(6')-Ib-cr, fosA3, floR_2, qnrB6, qnrS1, oqxA, oqxB, ARR-3, sul1, sul2, tet(A), tet(D), dfrA27*
NDM-Kpn-18	***bla***$_{\textbf{NDM-1}}$, ***bla***$_{\textbf{OXA-48}}$, *bla*$_{\text{CTX-M-15}}$, *bla*$_{\text{SHV-148}}$	*aac(6')-Ib, aph(3')-Ib, rmtF, strA, strB, mph(A), oqxA, oqxB, fosA3, fosA5, catA1, qnrB1, AAR-2, tet(A)*
NDM-Kpn-19	***bla***$_{\textbf{NDM-1}}$, *bla*$_{\text{CTX-M-15}}$, *bla*$_{\text{OXA-1}}$, *bla*$_{\text{TEM-1}}$, *bla*$_{\text{SHV-182}}$	*aph(3')-VI, armA, aac(6')-Ib-cr, qacE, oqxA, oqxB, fosA3, msr(E), mph(A), mph(E), catB3, catA1, qnrS1, sul1, sul2, tet(A), dfrA5, dfrA1*
NDM-Kpn-20	***bla***$_{\textbf{NDM-1}}$, *bla*$_{\text{CTX-M-15}}$, *bla*$_{\text{OXA-1}}$, *bla*$_{\text{TEM-1}}$, *bla*$_{\text{SHV-182}}$	*aph(3')-VI, armA, aac(6')-Ib-cr, qacE, oqxA, oqxB, fosA3, msr(E), mph(A), mph(E), catB3, catA1, qnrS1, sul1, sul2, tet(A), dfrA5, dfrA1*
NDM-Kpn-21	***bla***$_{\textbf{NDM-1}}$, *bla*$_{\text{CTX-M-15}}$, *bla*$_{\text{OXA-1}}$, *bla*$_{\text{OXA-9}}$, *bla*$_{\text{TEM-1}}$, *bla*$_{\text{SHV-67}}$	*aph(3')-VI, acc(6')-Ib, ant(3'')-Ia, aph(3')-Ia, armA, aac(6')-Ib-cr, qac(E), oqxA, oqxB, fosA5, msr(E), mph(E), mph(A), catB3, qnrB1, sul1, sul2, ARR-3, dfrA5*
NDM-Kpn-22	***bla***$_{\textbf{NDM-1}}$, *bla*$_{\text{CTX-M-15}}$, *bla*$_{\text{OXA-1}}$, *bla*$_{\text{SHV-182}}$	*acc(6')-Ib, aac(6')-Ib-cr, aac(3)-IIa, oqxA, oqxB, fosA3, catB3, dfrA14*
NDM-Kpn-23	***bla***$_{\textbf{NDM-1}}$, *bla*$_{\text{CTX-M-15}}$, *bla*$_{\text{TEM-1}}$, *bla*$_{\text{SHV-11}}$	*ant(3'')-Ia, armA, rmtB, qacE, oqxA, oqxB, fosA5, erm(B), msr(E), mph(A), mph(E), ere(A), cmlA1, qnrB1, ARR-2, sul1, dfrA12*

NDM-Kpn-24	***bla***$_{NDM-1}$, *bla*$_{CTX-M-15}$, *bla*$_{OXA-1}$, *bla*$_{TEM-1}$, *bla*$_{SHV-182}$	*aph(3')-VI, armA, aac(6')-Ib-cr, qac(E), oqxA, oqxB, fosA3, msr(E), mph(A), mph(E), catB3, catA1, qnrS1, sul1, sul2, tet(A), dfrA5, dfrA1*
NDM-Kpn-25	***bla***$_{NDM-5}$, *bla*$_{CTX-M-15}$, *bla*$_{TEM-1}$, *bla*$_{SHV-11}$	*ant(3'')-Ia, aadA2, armA, rmtB, qacE, oqxA, oqxB, fosA5, erm(B), msr(E), mph(A), mph(E), ere(A), cmlA1, qnrB1, ARR-2, sul1, dfrA12*
NDM-Kpn-26	***bla***$_{NDM-5}$, *bla*$_{CTX-M-15}$, *bla*$_{TEM-1}$, *bla*$_{SHV-11}$	*ant(3'')-Ia, aadA2, armA, rmtB, qacE, oqxA, oqxB, fosA5, erm(B), msr(E), mph(A), mph(E), ere(A), cmlA1, qnrB1, ARR-2, sul1, dfrA12*
NDM-Kpn-27	***bla***$_{NDM-5}$, *bla*$_{CTX-M-15}$, *bla*$_{TEM-1}$, *bla*$_{SHV-11}$	*ant(3'')-Ia, aadA2, armA, rmtB, qacE, oqxA, oqxB, fosA5, erm(B), msr(E), mph(A), mph(E), ere(A), cmlA1, qnrB1, ARR-2, sul1, dfrA12*
NDM-Kpn-28	***bla***$_{NDM-1}$, *bla*$_{CTX-M-15}$, *bla*$_{OXA-1}$, *bla*$_{TEM-1}$, *bla*$_{SHV-182}$	*aac(6')-Ib, aac(6')-Ib-cr, aadA2, aac(3')-IIa, qac(E), oqxA, oqxB, fosA3, mph(A), catB3, sul1, dfrA12, dfrA14*
NDM-Kpn-29	***bla***$_{NDM-5}$, *bla*$_{CTX-M-15}$, *bla*$_{TEM-1}$, *bla*$_{SHV-67}$	*ant(3'')-Ia, aadA2, armA, rmtB, qacE, oqxA, oqxB, fosA5, erm(B), msr(E), mph(A), mph(E), ere(A), cmlA1, qnrB1, ARR-2, sul1, dfrA12*
NDM-Kpn-30	***bla***$_{NDM-1}$, *bla*$_{CTX-M-15}$, *bla*$_{OXA-1}$, *bla*$_{SHV-182}$	*aph(3')-VI, armA, aac(6')-Ib-cr, qac(E), oqxA, oqxB, fosA3, msr(E), mph(A), mph(E), catB3, catA1, qnrS1, sul1, sul2, tet(A), dfrA5, dfrA1*
NDM-Kpn-31	***bla***$_{NDM-1}$, *bla*$_{CTX-M-15}$, *bla*$_{OXA-1}$, *bla*$_{OXA-9}$, *bla*$_{TEM-1}$, *bla*$_{SHV-11}$	*aph(6)-Id, aph(3')-VI, acc(2')-Ib, acc(6')-Ib, aadA1, aph(3')-Ia, armA, aac(6')-Ib-cr, qac(E), oqxA, oqxB, fosA3, fosA5, msr(E), mph(A), mph(E) erm(37), catB3, qnrB1, sul1, sul2, ARR-3, dfrA5*
NDM-Kpn-32	***bla***$_{NDM-1}$, *bla*$_{CTX-M-15}$, *bla*$_{SHV-11}$	*aph(3')-VI, aph(3')-Ia, armA, oqxA, oqxB, fosA3, fosA5, msr(E), mph(A), mph(E), qnrS1, sul2,*
NDM-Kpn-33	***bla***$_{NDM-1}$, ***bla***$_{KPC-3}$, *bla*$_{CMY-6}$, *bla*$_{OXA-9}$, *bla*$_{TEM-1}$, *bla*$_{SHV-182}$	*aph(3')-Ia, acc(6')-Ib, aadA2, rmtC, qac(E), oqxA, oqxB, fosA3, msr(E), mph(A), mph(E), catA1, qnrB1, sul1, dfrA12*
NDM-Kpn-34	***bla***$_{NDM-1}$, ***bla***$_{KPC-3}$, *bla*$_{CMY-6}$, *bla*$_{OXA-9}$, *bla*$_{TEM-1}$, *bla*$_{SHV-182}$	*aph(3')-Ia, acc(6')-Ib3, aadA2, rmtC, qac(E), oqxA, oqxB, fosA3, msr(E), mph(A), catA1, sul1, dfrA12*
NDM-Kpn-35	***bla***$_{NDM-1}$, *bla*$_{CTX-M-15}$, *bla*$_{TEM-1}$, *bla*$_{SHV-1}$	*aph(3')-VI, armA, aac(6')-Ib-cr, qac(E), oqxA, oqxB, fosA3, msr(E), mph(A), mph(E), catB3, catA1, qnrS1, sul1, sul2, dfrA5*
NDM-Kpn-36	***bla***$_{NDM-1}$, *bla*$_{CTX-M-15}$, *bla*$_{OXA-1}$, *bla*$_{SHV-182}$	*aac(6')-Ib, aac(6')-Ib-cr, aac(3')-IIa, qac(E), oqxA, oqxB, fosA3, catB3, dfrA14*
NDM-Kpn-37	***bla***$_{NDM-1}$, *bla*$_{CTX-M-15}$, *bla*$_{OXA-1}$, *bla*$_{SHV-182}$	*aac(6')-Ib, aac(6')-Ib-cr, aac(3')-Ia, qac(E), oqxA, oqxB, fosA3, catB3, dfrA14*
NDM-Kpn-38	***bla***$_{NDM-1}$, ***bla***$_{KPC-3}$, *bla*$_{OXA-9}$, *bla*$_{TEM-1}$, *bla*$_{SHV-182}$	*aph(3')-Ia, acc(6')-Ib3, aadA2, rmtC, qac(E), oqxA, oqxB, fosA3, mph(A), catA1, sul1, dfrA12*
NDM-Kpn-39	***bla***$_{NDM-1}$, *bla*$_{CTX-M-15}$, *bla*$_{OXA-1}$, *bla*$_{SHV-182}$	*aac(3')-IIa, aac(6')-Ib-cr, aadA2, aph(3')-Ia, rmtF, qac(E), oqxA, oqxB, fosA3, mph(A), catB3, qnrB1, ARR-2, sul1, dfrA12*
NDM-Kpn-40	***bla***$_{NDM-1}$, *bla*$_{CTX-M-55}$, *bla*$_{LAP-2}$, *bla*$_{SHV-51}$	*aph(3')-VI, qac(E), oqxA, oqxB, fosA3, mph(A), catA1, sul1, sul2, qnrS1, dfrA5, tet(A)*
NDM-Kpn-41	***bla***$_{NDM-1}$, *bla*$_{CTX-M-15}$, *bla*$_{OXA-1}$, *bla*$_{OXA-9}$, *bla*$_{TEM-1}$, *bla*$_{SHV-67}$	*aadA1, aph(3')-VI, armA, aac(6')-Ib-cr, qac(E), oqxA, oqxB, fosA5, msr(E), mph(A), mph(E), catB3, qnrS1, AAR-3, sul1, sul2, dfrA5*
NDM-Kpn-42	***bla***$_{NDM-1}$, *bla*$_{CTX-M-15}$, *bla*$_{OXA-1}$, *bla*$_{SHV-182}$	*aac(3')-IIa, aac(6')-Ib-cr, aac(6')-Ib, oqxA, oqxB, fosA3, catB3, dfrA14*
NDM-Kpn-43	***bla***$_{NDM-1}$, *bla*$_{CTX-M-15}$, *bla*$_{OXA-1}$, *bla*$_{TEM-1}$, *bla*$_{SHV-12}$	*ant(2'')-Ia, aac(6')-Ib, aac(6')-Ib-cr, aph(3')-Ia, oqxA, oqxB, fosA3, catB3, qnrB19, dfrA14*
NDM-Kpn-44	***bla***$_{NDM-1}$, ***bla***$_{KPC-3}$, *bla*$_{CMY-6}$, *bla*$_{OXA-9}$, *bla*$_{TEM-1}$, *bla*$_{SHV-182}$	*aph(3')-Ia, acc(6')-Ib, aadA2, rmtC, qac(E), oqxA, oqxB, fosA3, mph(A), catA1, sul1, dfrA12*
NDM-Kpn-45	***bla***$_{NDM-1}$, *bla*$_{CTX-M-15}$, *bla*$_{OXA-1}$, *bla*$_{OXA-9}$, *bla*$_{TEM-1}$, *bla*$_{SHV-67}$	*aph(3')-VI, ant(3'')-Ia, aac(6')-Ib-cr, acc(6')-Ib, acc(3)-IIa, qac(E), oqxA, oqxB, fosA5, ere(A), catB3, catA1, cmlA1, qnrS1, AAR-2, sul1, tet(A)*
NDM-Kpn-46	***bla***$_{NDM-1}$, *bla*$_{CTX-M-15}$, *bla*$_{OXA-1}$, *bla*$_{OXA-9}$, *bla*$_{TEM-1}$, *bla*$_{SHV-67}$	*aph(3')-VI, ant(3'')-Ia, aac(6')-Ib-cr, acc(6')-Ib, acc(3)-IIa, qac(E), oqxA, oqxB, fosA5, ere(A), catB3, catA1, cmlA1, qnrS1, AAR-2, sul1, tet(A)*
NDM-Kpn-47	***bla***$_{NDM-5}$, *bla*$_{DHA-1}$, *bla*$_{OXA-1}$, *bla*$_{TEM-1}$, *bla*$_{SHV-182}$	*aadA2, aph(3')-Ia, rmtB, aac(6')-Ib-cr, qacE, oqxA, oqxB, fosA5, mph(A), catB3, catA1, qnrB4, ARR-3, sul1, dfrA12*
NDM-Kpn-48	***bla***$_{NDM-1}$, *bla*$_{CTX-M-15}$, *bla*$_{OXA-1}$, *bla*$_{OXA-9}$, *bla*$_{TEM-1}$, *bla*$_{SHV-6}$	*aph(3')-VI, ant(3'')-Ia, aac(6')-Ib-cr, acc(6')-Ib, acc(3)-IIa, qac(E), oqxA, oqxB, fosA5, ere(A), catB3, catA1, cmlA1, qnrS1, AAR-2, sul1, tet(A)*

NDM-Kpn-49	***bla***$_{\textbf{NDM-1}}$, *bla*$_{\text{CTX-M-15}}$, *bla*$_{\text{OXA-9}}$, *bla*$_{\text{TEM-1}}$, *bla*$_{\text{SHV-11}}$	*aph(3')-VI, aadA1, aac(6')-Ib-cr, acc(3)-IIa, qac(E), oqxA, oqxB, fosA5, qnrS1, AAR-3, sul1*
NDM-Kpn-50	***bla***$_{\textbf{NDM-5}}$, *bla*$_{\text{CTX-M-15}}$, *bla*$_{\text{OXA-1}}$, *bla*$_{\text{TEM-1}}$, *bla*$_{\text{SHV-182}}$	*aadA2, aph(3')-Ia, rmtB, aac(6')-Ib-cr, qacE, oqxA, oqxB, fosA5, mph(A), catB3, catA1, qnrB4, ARR-3, sul1, dfrA12*
NDM-Kpn-51	***bla***$_{\textbf{NDM-1}}$, *bla*$_{\text{CTX-M-15}}$, *bla*$_{\text{OXA-1}}$, *bla*$_{\text{OXA-9}}$, *bla*$_{\text{TEM-1}}$, *bla*$_{\text{SHV-11}}$	*aadA2, aph(3')-I, rmtB, aac(6')-Ib-cr, qacE, oqxA, oqxB, fosA5, mph(A), catB3, catA1, qnrB4, ARR-3, sul1, dfrA12*
NDM-Kpn-52	***bla***$_{\textbf{NDM-5}}$, *bla*$_{\text{DHA-1}}$, *bla*$_{\text{OXA-1}}$, *bla*$_{\text{TEM-1}}$, *bla*$_{\text{SHV-182}}$	*aph(3')-VI, armA, aac(6')-Ib-cr, qac(E), oqxA, oqxB, fosA5, msr(E), mph(A), mph(E), catB3, catA1, qnrS1, sul1, sul2, dfrA5*
NDM-Kpn-53	***bla***$_{\textbf{NDM-1}}$, *bla*$_{\text{CTX-M-15}}$, *bla*$_{\text{OXA-1}}$, *bla*$_{\text{TEM-1}}$, *bla*$_{\text{SHV-1}}$	*aac(6')-Ib3, rmtC, qac(E), oqxA, oqxB, fosA5, sul1*
NDM-Kpn-54	***bla***$_{\textbf{NDM-1}}$, *bla*$_{\text{CMY-6}}$, *bla*$_{\text{TEM-1}}$, *bla*$_{\text{SHV-182}}$	*aadA2, aac(6')-Ib-cr, rmtB, qacE, oqxA, oqxB, fosA5, mph(A), erm(B), catB3, qnrS1, ARR-3, sul1, dfrA12*
NDM-Kpn-55	***bla***$_{\textbf{NDM-5}}$, *bla*$_{\text{CTX-M-15}}$, *bla*$_{\text{OXA-1}}$, *bla*$_{\text{TEM-1}}$, *bla*$_{\text{SHV-182}}$	*aph(3')-VI, aadA2, armA, qac(E), oqxA, oqxB, fosA5, msr(E), mph(A), mph(E), sul1, tet(A), dfrA12*
NDM-Kpn-56	***bla***$_{\textbf{NDM-1}}$, ***bla***$_{\textbf{OXA-48}}$, *bla*$_{\text{CTX-M-15}}$, *bla*$_{\text{SHV-11}}$	*aph(3')-VI, aac(6')-Ib-cr, armA1, qac(E), oqxA1, oqxB1, fosA3, msr(E), mph(A), mph(E), catB3, catA1, qnrS1, sul1, sul2, dfrA5*
NDM-Kpn-57	***bla***$_{\textbf{NDM-1}}$, *bla*$_{\text{CTX-M-15}}$, *bla*$_{\text{OXA-1}}$, *bla*$_{\text{TEM-1}}$, *bla*$_{\text{SHV-1}}$	*aac(6')-Ib-3, rmtC, qac(E), oqxA, oqxB, fosA3, sul1*
NDM-Kpn-58	***bla***$_{\textbf{NDM-1}}$, *bla*$_{\text{CMY-6}}$, *bla*$_{\text{CTX-M-15}}$, *bla*$_{\text{SHV-100}}$	*aph(3')-VI, armA1, qac(E), oqxA1, oqxB1, fosA5, msr(E), mph(A), mph(E), catB3, qnrS1, ARR-3, sul1, sul2, dfrA5*
NDM-Kpn-59	***bla***$_{\textbf{NDM-1}}$, *bla*$_{\text{CTX-M-15}}$, *bla*$_{\text{OXA-1}}$, *bla*$_{\text{OXA-9}}$, *bla*$_{\text{TEM-1}}$, *bla*$_{\text{SHV-11}}$	*aph(3')-VI, aac(6')-Ib, ant(3'')-Ia, armA1, aac(6')-Ib-cr, qac(E), oqxA1, oqxB1, fosA5, msr(E), mph(A), mph(E), catB3, qnrS1, ARR-3, sul1, sul2, dfrA5*
NDM-Kpn-60	***bla***$_{\textbf{NDM-1}}$, *bla*$_{\text{CTX-M-15}}$, *bla*$_{\text{OXA-1}}$, *bla*$_{\text{OXA-9}}$, *bla*$_{\text{TEM-1}}$, *bla*$_{\text{SHV-11}}$	*aph(3')-VI, aac(6')-Ib, ant(3'')-Ia, armA1, aac(6')-Ib-cr, qac(E), oqxA1, oqxB1, fosA3, fosA5, msr(E), mph(A), mph(E), catB3, qnrS1, ARR-3, sul1, sul2, dfrA5*
NDM-Kpn-61	***bla***$_{\textbf{NDM-1}}$, *bla*$_{\text{CTX-M-15}}$, *bla*$_{\text{OXA-1}}$, *bla*$_{\text{OXA-9}}$, *bla*$_{\text{TEM-1}}$, *bla*$_{\text{SHV-67}}$	*aac(6')-Ib-cr, aac(6')-Ib-1, acc(3)-IIa, oqxA1, oqxB1, fosA3, catB3, dfrA14*
NDM-Kpn-62	***bla***$_{\textbf{NDM-1}}$, ***bla***$_{\textbf{KPC-3}}$, *bla*$_{\text{CTX-M-15}}$, *bla*$_{\text{OXA-1}}$, *bla*$_{\text{SHV-182}}$	*aac(6')-Ib-1, aac(6')-Ib-cr, acc(3)-IIa, oqxA1, oqxB1, fosA3, catB3, dfrA14*
NDM-Kpn-63	***bla***$_{\textbf{NDM-1}}$, *bla*$_{\text{CTX-M-15}}$, *bla*$_{\text{OXA-1}}$, *bla*$_{\text{SHV-182}}$	*aph(3')-VI, armA1, qacE, oqxA1, oqxB1, fosA3, msr(E), mph(A), mph(E), qnrS1, sul1, sul2, dfrA5*
NDM-Kpn-64	***bla***$_{\textbf{NDM-1}}$, *bla*$_{\text{SHV-27}}$	*aph(3')-VI, aadA2, armA, qacE, oqxA1, oqxB1, fosA5, msr(E), mph(A), mph(E), sul1, tet(A), dfrA12*
NDM-Kpn-65	***bla***$_{\textbf{NDM-1}}$, ***bla***$_{\textbf{OXA-48}}$, *bla*$_{\text{CTX-M-15}}$, *bla*$_{\text{SHV-67}}$	*aph(3')-VI, aac(6')-Ib-1, ant(3'')-Ia, armA1, aac(6')-Ib-cr, qacE, oqxA1, oqxB1, fosA5, msr(E), mph(A), mph(E), catB3, qnrS1, ARR-3, sul1, sul2, dfrA5*
NDM-Kpn-66	***bla***$_{\textbf{NDM-1}}$, *bla*$_{\text{CTX-M-15}}$, *bla*$_{\text{OXA-1}}$, *bla*$_{\text{OXA-9}}$, *bla*$_{\text{TEM-1}}$, *bla*$_{\text{SHV-11}}$	*aph(3')-VI, aac(6')-Ib-1, aadA1, armA1, aac(6')-Ib-cr, qacE, oqxA1, oqxB1, fosA5, msr(E), mph(A), mph(E), catB3, qnrS1, ARR-3, sul1, sul2, dfrA5*
NDM-Kpn-67	***bla***$_{\textbf{NDM-1}}$, *bla*$_{\text{CTX-M-15}}$, *bla*$_{\text{OXA-1}}$, *bla*$_{\text{OXA-9}}$, *bla*$_{\text{TEM-1}}$, *bla*$_{\text{SHV-11}}$	*aph(3')-VI, aac(6')-Ib-1, ant(3'')-Ia, armA1, aac(6')-Ib-cr, qacE, oqxA1, oqxB1, fosA5, msr(E), mph(A), mph(E), catB3, qnrS1, ARR-3, sul1, sul2, dfrA5*
NDM-Kpn-68	***bla***$_{\textbf{NDM-1}}$, *bla*$_{\text{CTX-M-15}}$, *bla*$_{\text{OXA-1}}$, *bla*$_{\text{OXA-9}}$, *bla*$_{\text{TEM-1}}$, *bla*$_{\text{SHV-11}}$	*aph(3')-VI, aac(6')-Ib-1, ant(3'')-Ia, aac(6')-Ib-cr, qacE, oqxA1, oqxB1, fosA5, msr(E), mph(A), mph(E), catB3, qnrS1, ARR-3, sul1, sul2, dfrA5*
NDM-Kpn-69	***bla***$_{\textbf{NDM-1}}$, *bla*$_{\text{CTX-M-15}}$, *bla*$_{\text{OXA-1}}$, *bla*$_{\text{OXA-9}}$, *bla*$_{\text{TEM-1}}$, *bla*$_{\text{SHV-11}}$	*aac(6')-Ib-1, ant(3'')-Ia, aac(6')-Ib-cr, aac(3')-IIa, oqxA1, oqxB1, fosA5, catB3, dfrA14*
NDM-Kpn-70	***bla***$_{\textbf{NDM-1}}$, *bla*$_{\text{CTX-M-15}}$, *bla*$_{\text{OXA-1}}$, *bla*$_{\text{TEM-1A}}$, *bla*$_{\text{SHV-12}}$	*aadA2, aph(3')-I, rmtB, aac(6')-Ib-cr, qacE, oqxA, oqxB, fosA5, mph(A), catB3, catA1, qnrB4, ARR-3, sul1, dfrA12*
NDM-Kpn-71	***bla***$_{\textbf{NDM-1}}$, *bla*$_{\text{SHV-27}}$, *bla*$_{\text{SHV-1}}$	*aph(3')-VI, armA1, qacE, oqxA1, oqxB1, fosA3, msr(E), mph(A), mph(E), qnrS1, sul1, sul2, tet(D), dfrA5*
NDM-Kpn-72	***bla***$_{\textbf{NDM-5}}$, *bla*$_{\text{CTX-M-15}}$, *bla*$_{\text{OXA-1}}$, *bla*$_{\text{TEM-1}}$, *bla*$_{\text{SHV-11}}$	*aph(6')-Id, aadA2, aac(6')-Ib-cr, rmtB, aph(3')-Ib, qacE, oqxA1, oqxB1, fosA5, mph(A), erm(B), catB3, qnrB1, sul1, sul2, dfrA12*
NDM-Kpn-73	***bla***$_{\textbf{NDM-1}}$, *bla*$_{\text{CTX-M-15}}$, *bla*$_{\text{OXA-1}}$, *bla*$_{\text{SHV-182}}$	*aac(6')-Ib-1, aac(6')-Ib-cr, aac(3')-IIa, oqxA1, oqxB1, fosA5, catB3, dfrA14*

NDM-Kpn-74	***bla***$_{NDM-1}$, ***bla***$_{OXA-48}$, *bla*$_{CTX-M-15}$, *bla*$_{SHV-67}$	*aph(3’)-VI, aadA2, armA1, qacE, oqxA1, oqxB1, fosA5, msr(E), mph(A), mph(E), sul1, tet(A), dfrA12*
NDM-Kpn-75	***bla***$_{NDM-1}$, *bla*$_{CTX-M-15}$, *bla*$_{OXA-1}$, *bla*$_{OXA-9}$, *bla*$_{TEM-1}$, *bla*$_{SHV-11}$	*aph(3’)-VI, aac (6’)-Ib-1, aadA1, aac (6’)-Ib-cr, qacE, oqxA1, oqxB1, fosA5, catB3, qnrS1, ARR-3, sul1*
NDM-Kpn-76	***bla***$_{NDM-1}$, *bla*$_{SHV-27}$, *bla*$_{SHV-1}$	*aph(3’)-VI, armA1, qacE, oqxA1, oqxB1, fosA3, msr(E), mph(A), mph(E), qnrS1, sul1, sul2, tet(D), dfrA5*
NDM-Kpn-77	***bla***$_{NDM-1}$, ***bla***$_{OXA-48}$, *bla*$_{CTX-M-15}$, *bla*$_{SHV-11}$	*aph(3’)-VI, aadA2, armA1, qacE, oqxA1, oqxB1, fosA5, msr(E), mph(A), mph(E), sul1, tet(A), dfrA12*
NDM-Kpn-78	***bla***$_{NDM-5}$, *bla*$_{CTX-M-15}$, *bla*$_{SHV-27}$	*aph(6)-Id, aadA2, aph(3’)-Ib, qacE, oqxA1, oqxB1, fosA3, mph(A), catA2, qnrS1, sul1, sul2, dfrA12*
NDM-Kpn-79	***bla***$_{NDM-1}$, *bla*$_{SHV-1}$	*aph(3’)-VI, armA1, qacE, oqxA1, oqxB1, fosA3, msr(E), mph(A), mph(E), qnrS1, sul1, sul2, tet(D), dfrA5*
NDM-Kpn-80	***bla***$_{NDM-1}$, *bla*$_{OXA-10}$, *bla*$_{SHV-33}$	*ant(2’’)-Ia, aadA1, armA, qacE, oqxA, oqxB, fosA3, fosA6, msr(E), mph(E), cmlA5, ARR-2, sul1, sul3, dfrA12*
NDM-Kpn-81	***bla***$_{NDM-1}$, *bla*$_{CTX-M-15}$, *bla*$_{OXA-1}$, *bla*$_{OXA-9}$, *bla*$_{TEM-1}$, *bla*$_{SHV-11}$	*aph(3’)-VI, aac (6’)-Ib-1, ant(3’’)-Ia, aac(6’)-Ib-cr, aadA2, armA, qacE, oqxA1, oqxB1, fosA5, msr(E), mph(A), mph(E), qnrS1, catB3, sul1, sul2, dfrA5*
NDM-Kpn-82	***bla***$_{NDM-1}$, ***bla***$_{OXA-48}$, *bla*$_{CTX-M-15}$, *bla*$_{SHV-11}$	*aph(3’)-VI, aadA2, armA, qacE, oqxA1, oqxB1, fosA5, msr(E), mph(A), mph(E), sul1, tet(A), dfrA12*
NDM-Kpn-83	***bla***$_{NDM-1}$, ***bla***$_{OXA-48}$, *bla*$_{CTX-M-15}$, *bla*$_{SHV-11}$	*aph(3’)-VI, aadA2, armA, qacE, oqxA1, oqxB1, fosA5, msr(E), mph(A), mph(E), sul1, tet(A), dfrA12*
NDM-Kpn-84	***bla***$_{NDM-1}$, *bla*$_{CTX-M-15}$, *bla*$_{OXA-1}$, *bla*$_{OXA-9}$, *bla*$_{SHV-11}$	*aac(6’)-Ib, aac(6’)-Ib-cr, aac(3’)-IIa, oqxA1, oqxB1, fosA3, catB3, dfrA14*
NDM-Kpn-85	***bla***$_{NDM-1}$, ***bla***$_{OXA-48}$, *bla*$_{CTX-M-15}$, *bla*$_{OXA-9}$, *bla*$_{SHV-11}$	*aph(3’)-VI, aadA2, armA, qacE, oqxA1, oqxB1, fosA5, msr(E), mph(A), mph(E), sul1, tet(A), dfrA12*
NDM-Kpn-86	***bla***$_{NDM-1}$, *bla*$_{CTX-M-15}$, *bla*$_{OXA-1}$, *bla*$_{OXA-9}$, *bla*$_{TEM-1}$, *bla*$_{SHV-11}$	*aph(3’)-VI, aac (6’)-Ib-1, ant(3’’)-Ia, aac(6’)-Ib-cr, armA, qacE, oqxA1, oqxB1, fosA5, msr(E), mph(A), mph(E), qnrS1, catB3, ARR-3, sul1, sul2, dfrA5*
NDM-Kpn-87	***bla***$_{NDM-1}$, ***bla***$_{VIM-1}$, *bla*$_{CTX-M-15}$, *bla*$_{TEM-1A}$, *bla*$_{SHV-11}$	*aph(3’)-VI, aac (6’)-Ib-1, ant(3’’)-Ia, aac(6’)-Ib-cr, qacE, oqxA1, oqxB1, fosA5, mph(A), qnrS1, catB2, ARR-3, sul1, dfrA12*
NDM-Kpn-88	***bla***$_{NDM-1}$, ***bla***$_{VIM-1}$, *bla*$_{CTX-M-15}$, *bla*$_{OXA-9}$, *bla*$_{TEM-1A}$, *bla*$_{SHV-12}$	*aph(3’)-VI, aph(3’)-XV, aac (6’)-Ib-1, ant(3’’)-Ia, aac(6’)-Ib-cr, qacE, oqxA1, oqxB1, fosA5, mph(A), qnrS1, catB2, ARR-3, sul1, dfrA14*
NDM-Kpn-89	***bla***$_{NDM-1}$, *bla*$_{TEM-1A}$, *bla*$_{SHV-11}$	*ant(2’’)-Ia, aac(6’)-Ib, aph(3’)-Ia, oqxA1, oqxB1, fosA3, qnrB19, dfrA14*
NDM-Kpn-90	***bla***$_{NDM-1}$, ***bla***$_{VIM-1}$, *bla*$_{CTX-M-15}$, *bla*$_{OXA-9}$, *bla*$_{TEM-1A}$, *bla*$_{SHV-12}$	*aph(3’)-VI, aph(3’)-XV, aac (6’)-Ib-1, ant(3’’)-Ia, aac(6’)-Ib-cr, qacE, oqxA1, oqxB1, fosA5, mph(A), qnrS1, catB2, ARR-3, sul1, dfrA14*
NDM-Kpn-91	***bla***$_{NDM-1}$, ***bla***$_{OXA-48}$, *bla*$_{CTX-M-15}$, *bla*$_{SHV-67}$	*aph(3’)-VI, aadA2, armA, qacE, oqxA1, oqxB1, fosA5, msr(E), mph(A), mph(E), sul1, tet(A), dfrA12*
NDM-Kpn-92	***bla***$_{NDM-1}$, *bla*$_{SHV-27}$	*aph(3’)-VI, armA, qacE, oqxA1, oqxB1, fosA5, msr(E), mph(A), mph(E), sul1, sul2, tet(D), dfrA5*
NDM-Kpn-93	***bla***$_{NDM-1}$, ***bla***$_{VIM-1}$, *bla*$_{CTX-M-15}$, *bla*$_{OXA-9}$, *bla*$_{TEM-1A}$, *bla*$_{SHV-11}$	*aph(3’)-XV, aac (6’)-Ib-1, ant(3’’)-Ia, qacE, oqxA1, oqxB1, fosA5, mph(A), qnrS1, catB2, sul1, dfrA14*
NDM-Kpn-94	***bla***$_{NDM-1}$, *bla*$_{CTX-M-15}$, *bla*$_{OXA-1}$, *bla*$_{TEM-1}$, *bla*$_{SHV-11}$	*aph(3’)-VI, armA, aac(6’)-Ib-cr , qacE, oqxA1, oqxB1, fosA5, msr(E), mph(A), mph(E), qnrS1, catB3, ARR-3, sul1, sul2, dfrA5*
NDM-Kpn-95	***bla***$_{NDM-1}$, *bla*$_{SHV-27}$	*aph(3’)-VI, armA, qacE, oqxA1, oqxB1, fosA3, msr(E), mph(A), mph(E), sul1, sul2, tet(D), dfrA5*
NDM-Kpn-96	***bla***$_{NDM-1}$, ***bla***$_{OXA-48}$, *bla*$_{CTX-M-15}$, *bla*$_{SHV-11}$	*aph(3’)-VI, aadA2, armA, qacE, oqxA1, oqxB1, fosA5, msr(E), mph(A), mph(E), sul1, tet(A), dfrA12*
NDM-Kpn-97	***bla***$_{NDM-1}$, *bla*$_{CTX-M-15}$, *bla*$_{CMY-6}$, *bla*$_{OXA-181}$, *bla*$_{TEM-1B}$, *bla*$_{SHV-28}$	*aac(6’)-Ib3, ant(3’’)-Ia, aph(3’)-Ia, aph(3’)-Ib, rmtC, qac(E), oqxA, oqxB, fosA5, erm(B), mph(A), catA2, qnrS1, sul1, sul2, tet(A), dfrA12*
NDM-Kpn-98	***bla***$_{NDM-1}$, *bla*$_{CTX-M-15}$, *bla*$_{OXA-1}$, *bla*$_{TEM-1A}$, *bla*$_{SHV-12}$	*ant(2’’)-Ia, aac(6’)-Ib, aph(3’)-Ia, aac(6’)-Ib-cr, oqxA1, oqxB1, fosA5, catB3, qnrB19, dfrA14*

NDM-Kpn-99	***bla***$_{\textbf{NDM-1}}$, *bla*$_{\text{CTX-M-15}}$, *bla*$_{\text{OXA-1}}$, *bla*$_{\text{SHV-182}}$	*aac(6')-Ib, aac(6')-Ib-cr, oqxA1, oqxB1, fosA3, catB3, dfrA14*
NDM-Kpn-100	***bla***$_{\textbf{NDM-5}}$, *bla*$_{\text{SHV-1}}$	*aadA2, qacE, oqxA, oqxB, fosA6, sul1, dfrA12*
NDM-Kpn-101	***bla***$_{\textbf{NDM-1}}$, *bla*$_{\text{CTX-M-15}}$, *bla*$_{\text{OXA-1}}$, *bla*$_{\text{TEM-1B}}$, *bla*$_{\text{SHV-182}}$	*aac(6')-Ib-cr, rmtC, qacE, oqxA1, oqxB1, fosA3, catB3, qnrS1, tet(A), dfrA1*
NDM-Kpn-102	***bla***$_{\textbf{NDM-1}}$, ***bla***$_{\textbf{VIM-1}}$, *bla*$_{\text{CTX-M-15}}$, *bla*$_{\text{OXA-9}}$, *bla*$_{\text{TEM-1A}}$, *bla*$_{\text{SHV-12}}$	*aph(3')-VI, aac (6')-Ib-1, ant(3'')-Ia, aac(6')-Ib-cr, qacE, oqxA1, oqxB1, fosA5, mph(A), qnrS1, catB2, ARR-3, sul1, dfrA14*
NDM-Kpn-103	***bla***$_{\textbf{NDM-1}}$, ***bla***$_{\textbf{OXA-48}}$, *bla*$_{\text{CTX-M-15}}$, *bla*$_{\text{SHV-11}}$	*aph(3')-VI, aadA2, armA, qacE, oqxA1, oqxB1, fosA5, msr(E), mph(A), mph(E), sul1, tet(A), dfrA12*
NDM-Kpn-104	***bla***$_{\textbf{NDM-1}}$, *bla*$_{\text{CTX-M-15}}$, *bla*$_{\text{OXA-1}}$, *bla*$_{\text{SHV-182}}$	*aac(6')-Ib, aac(6')-Ib-cr, oqxA1, oqxB1, fosA3, catB3, dfrA14*
NDM-Kpn-105	***bla***$_{\textbf{NDM-1}}$, *bla*$_{\text{SHV-27}}$	*aph(3')-VI, armA, msr(E), mph(A), mph(E), qnrS1, sul1, sul2, tet(D), dfrA5*
NDM-Kpn-106	***bla***$_{\textbf{NDM-1}}$, *bla*$_{\text{CTX-M-15}}$, *bla*$_{\text{OXA-1}}$, *bla*$_{\text{SHV-1}}$	*aac(6')-Ib, aac(6')-Ib-cr, aac(3')-IIa, oqxA1, oqxB1, fosA3, catB3, dfrA14*
NDM-Kpn-107	***bla***$_{\textbf{NDM-5}}$, ***bla***$_{\textbf{OXA-48}}$, *bla*$_{\text{CTX-M-15}}$, *bla*$_{\text{CTX-M-14b}}$, *bla*$_{\text{OXA-9}}$, *bla*$_{\text{TEM-1C}}$, *bla*$_{\text{SHV-1}}$	*aac(6')-Ib, aph(3')-Ia, aph(3')-Ib, aph(3')-VI, aph(6')-Id, aadA1, oqxA1, oqxB1, fosA3, catB3, mph(A), catA1, qnrS1, sul2, tet(A)*
NDM-Kpn-108	***bla***$_{\textbf{NDM-1}}$, *bla*$_{\text{CTX-M-15}}$, *bla*$_{\text{OXA-1}}$, *bla*$_{\text{TEM-1A}}$, *bla*$_{\text{SHV-12}}$	*ant(2'')-Ia, aac(6')-Ib, aph(3')-Ia, aac(6')-Ib-cr, aac(3')-IIa, oqxA1, oqxB1, fosA3, catB3, qnrB19, dfrA14*
NDM-Kpn-109	***bla***$_{\textbf{NDM-1}}$, *bla*$_{\text{CTX-M-15}}$, *bla*$_{\text{OXA-1}}$, *bla*$_{\text{SHV-11}}$	*aac(6')-Ib, aac(6')-Ib-cr, oqxA1, oqxB1, fosA3, catB3, dfrA14*
NDM-Kpn-110	***bla***$_{\textbf{NDM-1}}$, *bla*$_{\text{CTX-M-15}}$, *bla*$_{\text{OXA-1}}$, *bla*$_{\text{SHV-11}}$	*aac(6')-Ib, aac(6')-Ib-cr, oqxA1, oqxB1, fosA3, catB3, dfrA14*
NDM-Kpn-111	***bla***$_{\textbf{NDM-5}}$, *bla*$_{\text{CTX-M-15}}$, *bla*$_{\text{OXA-1}}$, *bla*$_{\text{OXA-9}}$, *bla*$_{\text{TEM-1A}}$, *bla*$_{\text{SHV-11}}$	*aac(6')-Ib, aph(3')-VI, aadA1, aac(6')-Ib-cr, qacE, oqxA1, oqxB1, fosA5, catB3, qnrS1, ARR-3, sul1*
NDM-Kpn-112	***bla***$_{\textbf{NDM-5}}$, *bla*$_{\text{CTX-M-15}}$, *bla*$_{\text{OXA-1}}$, *bla*$_{\text{OXA-9}}$, *bla*$_{\text{TEM-1A}}$, *bla*$_{\text{SHV-11}}$	*aph(6')-Id, aac(6')-Ib, aac(3')-II, aph(3')-VI, aph(3'')-Ib, aadA1, qacE, oqxA1, oqxB1, fosA5, mph(A), floR, catA1, qnrS1, sul1, sul2, tet(A), dfrA5*
NDM-Kpn-113	***bla***$_{\textbf{NDM-5}}$, ***bla***$_{\textbf{OXA-48}}$, *bla*$_{\text{CTX-M-15}}$, *bla*$_{\text{SHV-11}}$	*aph(3')-VI, aadA1, armA, qacE, oqxA1, fosA5, msrE, mph(A), mph(E), sul1, tet(A), dfrA12*
NDM-Kpn-114	***bla***$_{\textbf{NDM-1}}$, *bla*$_{\text{CTX-M-15}}$, *bla*$_{\text{OXA-1}}$, *bla*$_{\text{SHV-1}}$	*aac(6')-Ib, aac(6')-Ib-cr, oqxA1, oqxB1, fosA3, catB3, dfrA14*
NDM-Kpn-115	***bla***$_{\textbf{NDM-1}}$, *bla*$_{\text{CTX-M-15}}$, *bla*$_{\text{OXA-1}}$, *bla*$_{\text{TEM-1A}}$, *bla*$_{\text{SHV-12}}$	*ant(2'')-Ia, aac(6')-Ib, aph(3')-Ia, aac(6')-Ib-cr, aac(3')-IIa, oqxA1, oqxB1, fosA3, catB3, qnrB19, dfrA14*
NDM-Kpn-116	***bla***$_{\textbf{NDM-1}}$, ***bla***$_{\textbf{VIM-1}}$, *bla*$_{\text{CTX-M-15}}$, *bla*$_{\text{OXA-9}}$, *bla*$_{\text{TEM-1A}}$, *bla*$_{\text{SHV-12}}$	*aph(3')-VI, aac (6')-Ib-1, ant(3'')-Ia, aac(6')-Ib-cr, qacE, oqxA1, oqxB1, fosA5, mph(A), qnrS1, catB2, ARR-3, sul1, dfrA14*
NDM-Kpn-117	***bla***$_{\textbf{NDM-5}}$, *bla*$_{\text{OXA-10}}$, *bla*$_{\text{SHV-1}}$	*aac(6')-II, aph(3')-VI, qacE, oqxA1, oqxB1, fosA3, qnrS1, sul1*
NDM-Kpn-118	***bla***$_{\textbf{NDM-1}}$, *bla*$_{\text{CTX-M-15}}$, *bla*$_{\text{OXA-1}}$, *bla*$_{\text{SHV-182}}$	*aac(6')-Ib, aac(6')-Ib-cr, oqxA1, oqxB1, fosA3, catB3, dfrA14*
NDM-Kpn-119	***bla***$_{\textbf{NDM-1}}$, *bla*$_{\text{CTX-M-15}}$, *bla*$_{\text{OXA-1}}$, *bla*$_{\text{SHV-11}}$	*aph(3')-VI, aph(3)-Ia, armA, aac(6')-Ib-cr, qacE, oqxA1, oqxB1, fosA5, mph(A), mph(E), qnrS1, catB3, ARR-3, sul1, sul2, dfrA14*
NDM-Kpn-120	***bla***$_{\textbf{NDM-1}}$, *bla*$_{\text{CTX-M-15}}$, *bla*$_{\text{OXA-1}}$, *bla*$_{\text{OXA-9}}$, *bla*$_{\text{TEM-1A}}$, *bla*$_{\text{SHV-11}}$	*aph(3')-VI, aac(6')-Ib, ant(3'')-Ia, armA, aac(6')-Ib-cr, qacE, oqxA1, oqxB1, fosA5, msrE, mph(A), mph(E), catB3, qnrS1, ARR-3, sul1, sul2, dfrA5*
NDM-Kpn-121	***bla***$_{\textbf{NDM-1}}$, ***bla***$_{\textbf{OXA-48}}$, *bla*$_{\text{CTX-M-15}}$, *bla*$_{\text{SHV-67}}$	*aph(3')-VI, aadA2, armA, qacE, oqxA1, oqxB1, fosA5, msr(E), mph(A), mph(E), sul1, tet(A), dfrA12*
NDM-Kpn-122	***bla***$_{\textbf{NDM-1}}$, *bla*$_{\text{CTX-M-15}}$, *bla*$_{\text{OXA-1}}$, *bla*$_{\text{TEM-1B}}$, *bla*$_{\text{SHV-182}}$	*aph(6')-Id, aph(3')-VI, aph(3')-VIa, armA, aac(6')-Ib-cr , qacE, oqxA1, oqxB1, fosA3, msr(E), mph(A), mph(E), qnrS1, catB3, catA1, sul1, sul2, tet(A), dfrA5, dfrA1*
NDM-Kpn-123	***bla***$_{\textbf{NDM-1}}$, ***bla***$_{\textbf{VIM-1}}$, *bla*$_{\text{CTX-M-15}}$, *bla*$_{\text{OXA-9}}$, *bla*$_{\text{TEM-1A}}$, *bla*$_{\text{SHV-12}}$	*aph(3')-VI, aac (6')-Ib-1, ant(3'')-Ia, aac(6')-Ib-cr, qacE, oqxA1, oqxB1, fosA5, mph(A), qnrS1, catB2, ARR-3, sul1, dfrA14*

NDM-Kpn-124	***bla***$_{\mathbf{NDM\text{-}5}}$, ***bla***$_{\mathbf{OXA\text{-}48}}$, *bla*$_{CTX\text{-}M\text{-}15}$, *bla*$_{OXA\text{-}9}$, *bla*$_{TEM\text{-}1C}$, *bla*$_{SHV\text{-}1}$	*aac(6’)-Ib, aph(3’)-Ia, aph(3’)-Ib, aph(3’)-VI, aph(6’)-Id, aadA1, armA, qacE, oqxA1, oqxB1, fosA3, catB3, msr(E), mph(A), mph(E), catA1, qnrS1, sul1, sul2, tet(A), dfrA5*
NDM-Kpn-125	***bla***$_{\mathbf{NDM\text{-}1}}$, ***bla***$_{\mathbf{VIM\text{-}1}}$, *bla*$_{CTX\text{-}M\text{-}15}$, *bla*$_{OXA\text{-}9}$, *bla*$_{TEM\text{-}1A}$, *bla*$_{SHV\text{-}11}$	*aac(6’)-Ib, aac(6’)-Ib-cr, oqxA1, oqxB1, fosA3, catB3, dfrA14*
NDM-Kpn-126	***bla***$_{\mathbf{NDM\text{-}5}}$, ***bla***$_{\mathbf{OXA\text{-}48}}$, *bla*$_{CTX\text{-}M\text{-}15}$, *bla*$_{SHV\text{-}11}$	*aph(3’)-VI, aadA2, armA, qacE, oqxA1, fosA5, msr(E), mph(A), mph(E), sul1, tet(A), dfrA12*
NDM-Kpn-127	***bla***$_{\mathbf{NDM\text{-}1}}$, *bla*$_{CTX\text{-}M\text{-}15}$, *bla*$_{OXA\text{-}1}$, *bla*$_{TEM\text{-}1B}$, *bla*$_{SHV\text{-}27}$	*aph(6’)-Id, aac(3)-IIa, aac(6')-Ib-cr, aph(3')-VI, aph(3'')-Ib, armA, qacE, oqxA1, oqxB1, fosA3, mrs(E), mph(A), mph(E) qnrB1, qnrS1, catB3, sul1, sul2, dfrA14, dfrA5*
NDM-Kpn-128	***bla***$_{\mathbf{NDM\text{-}1}}$, *bla*$_{CTX\text{-}M\text{-}15}$, *bla*$_{OXA\text{-}1}$, *bla*$_{OXA\text{-}9}$, *bla*$_{TEM\text{-}1A}$, *bla*$_{SHV\text{-}11}$	*aac(6')-Ib', aph(3')-VI, ant(3'')-Ia, aac(6')-Ib-cr, armA, qacE, oqxA1, oqxB1, fosA5, mrs(E), mph(A), mph(E), qnrS1, catB3, ARR-3, sul1, sul2, dfrA5*
NDM-Kpn-129	***bla***$_{\mathbf{NDM\text{-}1}}$, ***bla***$_{\mathbf{OXA\text{-}48}}$, *bla*$_{CTX\text{-}M\text{-}15}$, *bla*$_{SHV\text{-}11}$	*aadA2, aph(3')-VI, armA, qacE, oqxA1, fosA5, mrs(E), mph(A), mph(E), sul1, tet(A), dfrA12*
NDM-Kpn-130	***bla***$_{\mathbf{NDM\text{-}1}}$, ***bla***$_{\mathbf{KPC\text{-}3}}$, *bla*$_{CTX\text{-}M\text{-}15}$, *bla*$_{OXA\text{-}1}$, *bla*$_{OXA\text{-}9}$, *bla*$_{TEM\text{-}1A}$, *bla*$_{SHV\text{-}182}$	*aac(6')-Ib, aac(6')-Ib-cr, oqxA1, oqxB1, fosA3, catB3, dfrA14*
NDM-Kpn-131	***bla***$_{\mathbf{NDM\text{-}1}}$, *bla*$_{TEM\text{-}1D}$, *bla*$_{SHV\text{-}12}$	*aac(6')-Ib, ant(2'')-Ia, aph(3')-Ia, oqxA1, oqxB1, fosA3, qnrB19, dfrA14*
NDM-Kpn-132	***bla***$_{\mathbf{NDM\text{-}1}}$, *bla*$_{CTX\text{-}M\text{-}15}$, *bla*$_{OXA\text{-}1}$, *bla*$_{OXA\text{-}9}$, *bla*$_{TEM\text{-}1A}$, *bla*$_{SHV\text{-}11}$	*aac(6')-Ib-cr, aadA, aph(3')-VI, armA, aac(6')-Ib, ant(3'')-Ia, qacE, oqxA1, oqxB1, fosA5, mrs(E), mph(A), mph(E), qnrS1, catB3, ARR-3, sul1, sul2, dfrA5*
NDM-Kpn-133	***bla***$_{\mathbf{NDM\text{-}1}}$, ***bla***$_{\mathbf{OXA\text{-}48}}$, *bla*$_{CTX\text{-}M\text{-}15}$, *bla*$_{SHV\text{-}67}$	*aadA2, aph(3')-VI, armA, qacE, oqxA1, oqxB1, fosA5, mrs(E), mph(A), mph(E), sul1, tet(A), dfrA12*
NDM-Kpn-134	***bla***$_{\mathbf{NDM\text{-}1}}$, ***bla***$_{\mathbf{OXA\text{-}48}}$, *bla*$_{CTX\text{-}M\text{-}15}$, *bla*$_{SHV\text{-}11}$	*aadA2, aph(3')-VI, armA, qacE, oqxA1, oqxB1, fosA5, mrs(E), mph(A), mph(E), sul1, tet(A), dfrA12*
NDM-Kpn-135	***bla***$_{\mathbf{NDM\text{-}1}}$, ***bla***$_{\mathbf{VIM\text{-}1}}$, *bla*$_{CTX\text{-}M\text{-}15}$, *bla*$_{OXA\text{-}9}$, *bla*$_{TEM\text{-}1A}$, *bla*$_{SHV\text{-}12}$	*aac(6')-Ib-cr, aph(3')-VI, aac(6')-Ib, ant(3'')-Ia, aph(3')-XV, qacE, oqxA1, oqxB1, fosA5, mph(A), ARR-3, qnrS1, catB2, sul1, dfrA14*
NDM-Kpn-136	***bla***$_{\mathbf{NDM\text{-}5}}$, *bla*$_{CTX\text{-}M\text{-}15}$, *bla*$_{CMY\text{-}2}$, *bla*$_{SHV\text{-}11}$, *bla*$_{OXA\text{-}9}$, *bla*$_{TEM\text{-}1A}$	*aac(3)-IId, aac(6')-Ib, aadA1, aph(3')-VI, aph(6)-Id, aph(3'')-Ib, qacE, oqxA1, oqxB1, fosA5, mph(A), catA1, qnrS1, floR, sul1, sul2, tet(A), dfrA5*
NDM-Kpn-137	***bla***$_{\mathbf{NDM\text{-}1}}$, ***bla***$_{\mathbf{VIM\text{-}1}}$, *bla*$_{CTX\text{-}M\text{-}15}$, *bla*$_{OXA\text{-}9}$, *bla*$_{TEM\text{-}1A}$, *bla*$_{SHV\text{-}12}$	*aph(3')-VI, aac(6')-Ib, ant(3'')-Ia, aph(3')-XV, aac(6')-Ib-cr, qacE, oqxA1, oqxB1, fosA5, mph(A), ARR-3, qnrS1, catB2, sul1, dfrA14*
NDM-Kpn-138	***bla***$_{\mathbf{NDM\text{-}1}}$, *bla*$_{CTX\text{-}M\text{-}15}$, *bla*$_{OXA\text{-}1}$, *bla*$_{SHV\text{-}182}$	*aac(3)-IIa, aac(6')-Ib, aac(6')-Ib-cr, oqxA1, oqxB1, fosA3, catB3, dfrA14*
NDM-Kpn-139	***bla***$_{\mathbf{NDM\text{-}1}}$, *bla*$_{SHV\text{-}27}$, *bla*$_{SHV\text{-}1}$	*aph(3')-VI, armA, qacE, oqxA1, oqxB1, fosA3, mph(A), msr(E), mph(E), qnrS1, sul1, sul2, tet(D), dfrA5*
NDM-Kpn-140	***bla***$_{\mathbf{NDM\text{-}1}}$, *bla*$_{CTX\text{-}M\text{-}15}$, *bla*$_{OXA\text{-}1}$, *bla*$_{SHV\text{-}182}$	*aac(3)-IIa, aac(6')-Ib, aac(6')-Ib-cr, oqxA1, oqxB1, fosA3, catB3, dfrA14*
NDM-Kpn-141	***bla***$_{\mathbf{NDM\text{-}1}}$, ***bla***$_{\mathbf{OXA\text{-}48}}$, *bla*$_{CTX\text{-}M\text{-}15}$, *bla*$_{SHV\text{-}67}$	*aadA2, aph(3')-VI, armA, qacE, oqxA1, fosA5, mph(A), msr(E), mph(E), sul1, tet(A), dfrA12*
NDM-Kpn-142	***bla***$_{\mathbf{NDM\text{-}1}}$, *bla*$_{CTX\text{-}M\text{-}15}$, *bla*$_{OXA\text{-}1}$	*aac(6')-Ib, aac(6')-Ib-cr, oqxA1, oqxB1, fosA3, catB3, dfrA14*
NDM-Kpn-143	***bla***$_{\mathbf{NDM\text{-}1}}$, ***bla***$_{\mathbf{OXA\text{-}48}}$, *bla*$_{CTX\text{-}M\text{-}15}$, *bla*$_{SHV\text{-}11}$	*aadA2, aph(3')-VI, armA, qacE, oqxA1, fosA5, mph(A), msr(E), mph(E), sul1, tet(A), dfrA12*
NDM-Kpn-144	***bla***$_{\mathbf{NDM\text{-}1}}$, ***bla***$_{\mathbf{VIM\text{-}1}}$, *bla*$_{CTX\text{-}M\text{-}15}$, *bla*$_{OXA\text{-}9}$, *bla*$_{TEM\text{-}1A}$, *bla*$_{SHV\text{-}12}$	*aph(3')-VI, aac(6')-Ib, aac(6')-Ib-cr, ant(3'')-Ia, aph(3')-XV, oqxA1, oqxB1, fosA5, mph(A), ARR-3, qnrS1, catB2, sul1, dfrA14*
NDM-Kpn-145	***bla***$_{\mathbf{NDM\text{-}1}}$, *bla*$_{CTX\text{-}M\text{-}15}$, *bla*$_{OXA\text{-}1}$, *bla*$_{OXA\text{-}9}$, *bla*$_{TEM\text{-}1A}$, *bla*$_{SHV\text{-}67}$	*aac(6')-Ib, aph(3')-VI, armA, ant(3'')-Ia, aac(6')-Ib-cr, qacE, oqxA1, oqxB1, fosA5, mrs(E), mph(A), mph(E), ARR-3, qnrS1, catB3, sul1, sul2, dfrA5*
NDM-Kpn-146	***bla***$_{\mathbf{NDM\text{-}1}}$, ***bla***$_{\mathbf{OXA\text{-}48}}$, *bla*$_{CTX\text{-}M\text{-}15}$, *bla*$_{SHV\text{-}11}$	*aadA2, aph(3')-VI, armA, qacE, oqxA1, oqxB1, fosA5, mrs(E), mph(A), mph(E), sul1, tet(A), dfrA12*
NDM-Kpn-147	***bla***$_{\mathbf{NDM\text{-}1}}$, ***bla***$_{\mathbf{VIM\text{-}1}}$, *bla*$_{CTX\text{-}M\text{-}15}$, *bla*$_{OXA\text{-}9}$, *bla*$_{TEM\text{-}1A}$, *bla*$_{SHV\text{-}12}$	*aph(3')-VI, aac(6')-Ib, ant(3'')-Ia, ant(3'')-Ia, aph(3')-XV, aac(6')-Ib-cr, qacE, oqxA1, oqxB1, fosA5, mph(A), qnrS1, catB2, ARR-3, sul1, dfrA14*
NDM-Kpn-148	***bla***$_{\mathbf{NDM\text{-}1}}$, ***bla***$_{\mathbf{OXA\text{-}48}}$, *bla*$_{CTX\text{-}M\text{-}15}$, *bla*$_{SHV\text{-}67}$	*aadA2, aph(3')-VI, armA, qacE, oqxA1, oqxB1, fosA5, mrs(E), mph(A), mph(E), sul1, tet(A), dfrA12*

NDM-Kpn-149	**$bla_{\text{NDM-1}}$, $bla_{\text{OXA-48}}$**, $bla_{\text{CTX-M-15}}$, $bla_{\text{SHV-11}}$	*aadA2, aph(3')-VI, armA, qacE, oqxA1, oqxB1, fosA5, mrs(E), mph(A), mph(E), sul1, tet(A), dfrA12*
NDM-Kpn-150	**$bla_{\text{NDM-1}}$, $bla_{\text{OXA-48}}$**, $bla_{\text{CTX-M-15}}$, $bla_{\text{SHV-11}}$	*aadA2, aph(3')-VI, armA, qacE, oqxA1, oqxB1, fosA5, mrs(E), mph(A), mph(E), sul1, tet(A), dfrA12*
NDM-Kpn-151	**$bla_{\text{NDM-1}}$**, $bla_{\text{CTX-M-15}}$, $bla_{\text{OXA-1}}$, $bla_{\text{SHV-182}}$	*aac(3)-IIa, aac(6')-Ib, aac(6')-Ib-cr, oqxA1, oqxB1, fosA3, catB3, dfrA14*
NDM-Kpn-152	**$bla_{\text{NDM-1}}$, $bla_{\text{VIM-1}}$**, $bla_{\text{CTX-M-15}}$, $bla_{\text{OXA-9}}$, $bla_{\text{TEM-1A}}$, $bla_{\text{SHV-12}}$	*aph(3')-VI, aac(6')-Ib, aac(6')-Ib-cr, ant(3'')-Ia, aph(3')-XV, qacE, oqxA1, oqxB1, fosA5, mph(A), ARR-3, qnrS1, catB2, sul1, dfrA14*
NDM-Kpn-153	**$bla_{\text{NDM-1}}$, $bla_{\text{OXA-48}}$**, $bla_{\text{CTX-M-15}}$, $bla_{\text{SHV-11}}$	*aph(3')-VI, aadA2, armA, qacE, oqxA1, oqxB1, fosA5, mrs(E), mph(A), mph(E), sul1, tet(A), dfrA12*
NDM-Kpn-154	**$bla_{\text{NDM-1}}$, $bla_{\text{KPC-3}}$**, $bla_{\text{OXA-9}}$, $bla_{\text{TEM-1A}}$, $bla_{\text{SHV-182}}$	*aac(6')-Ib', aadA2, aph(3')-Ia, qacE, oqxA1, oqxB1, fosA3, mph(A), catA1, sul1, dfrA12*
NDM-Kpn-155	**$bla_{\text{NDM-1}}$, $bla_{\text{OXA-48}}$**, $bla_{\text{CTX-M-15}}$, $bla_{\text{SHV-11}}$	*aph(3')-VI, aadA2, armA, qacE, oqxA1, oqxB1, fosA5, mrs(E), mph(A), mph(E), sul1, tet(A), dfrA12*
NDM-Kpn-156	**$bla_{\text{NDM-1}}$, $bla_{\text{OXA-48}}$**, $bla_{\text{CTX-M-15}}$, $bla_{\text{SHV-11}}$	*aph(3')-VI, aadA2, armA, qacE, oqxA1, fosA5, mrs(E), mph(A), mph(E), sul1, tet(A), dfrA12*
NDM-Kpn-157	**$bla_{\text{NDM-1}}$, $bla_{\text{OXA-48}}$**, $bla_{\text{CTX-M-15}}$, $bla_{\text{SHV-67}}$	*aph(3')-VI, aadA2, armA, qacE, oqxA1, fosA5, mrs(E), mph(A), mph(E), sul1, tet(A), dfrA12*
NDM-Kpn-158	**$bla_{\text{NDM-1}}$, $bla_{\text{OXA-48}}$**, $bla_{\text{CTX-M-15}}$, $bla_{\text{SHV-11}}$	*aph(3')-VI, aadA2, armA, qacE, oqxA1, oqxB1, fosA5, mrs(E), mph(A), mph(E), sul1, tet(A), dfrA12*
NDM-Kpn-159	**$bla_{\text{NDM-1}}$, $bla_{\text{OXA-48}}$**, $bla_{\text{CTX-M-15}}$, $bla_{\text{SHV-67}}$	*aph(3')-VI, aadA2, armA, qacE, oqxA1, oqxB1, fosA5, mrs(E), mph(A), mph(E), sul1, tet(A), dfrA12*
NDM-Kpn-160	**$bla_{\text{NDM-1}}$, $bla_{\text{OXA-48}}$**, $bla_{\text{CTX-M-15}}$, $bla_{\text{SHV-11}}$	*aph(3')-VI, aadA2, armA, qacE, oqxA1, oqxB1, fosA5, mrs(E), mph(A), mph(E), sul1, tet(A), dfrA12*
NDM-Kpn-161	**$bla_{\text{NDM-1}}$, $bla_{\text{CTX-M-15}}$**, $bla_{\text{OXA-1}}$, $bla_{\text{TEM-1B}}$, $bla_{\text{SHV-182}}$	*aac(3)-IIa, aac(6')-Ib, aadA2, aph(3')-Ia, aph(6)-Id, aac(6')-Ib-cr, aph(3'')-Ib, qacE, oqxA1, oqxB1, fosA5, fosA3, mph(A), catB3, catA1, sul1, sul2, dfrA12, dfrA14*
NDM-Kpn-162	**$bla_{\text{NDM-1}}$, $bla_{\text{VIM-1}}$**, $bla_{\text{CTX-M-15}}$, $bla_{\text{OXA-9}}$, $bla_{\text{TEM-1A}}$, $bla_{\text{SHV-12}}$	*aph(3')-VI, aac(6')-Ib, ant(3'')-Ia, aph(3')-XV, aac(6')-Ib-cr, qacE, oqxA1, oqxB1, fosA5, mph(A), catB2, qnrS1, ARR-3, sul1, dfrA14*
NDM-Kpn-163	**$bla_{\text{NDM-1}}$, $bla_{\text{OXA-48}}$**, $bla_{\text{CTX-M-15}}$, $bla_{\text{SHV-1}}$	*aph(3')-VI, aadA2, armA, qacE, oqxA1, oqxB1, fosA5, mrs(E) mph(A), mph(E), tet(A), sul1, dfrA12*
NDM-Kpn-164	**$bla_{\text{NDM-1}}$, $bla_{\text{VIM-1}}$**, $bla_{\text{CTX-M-15}}$, $bla_{\text{OXA-9}}$, $bla_{\text{TEM-1A}}$, $bla_{\text{SHV-12}}$	*aph(3')-VI, aac(6')-Ib, ant(3'')-Ia, aph(3')-XV, aac(6')-Ib-cr, qacE, oqxA1, oqxB1, fosA5, fosA3, mph(A), catB2, qnrS1, ARR-3, sul1, dfrA14*
NDM-Kpn-165	**$bla_{\text{NDM-1}}$, $bla_{\text{OXA-48}}$**, $bla_{\text{CTX-M-15}}$, $bla_{\text{SHV-11}}$	*aph(3')-VI, aadA2, armA, qacE, oqxA1, oqxB1, fosA5, mrs(E), mph(A), mph(E), sul1, dfrA12, tet(A)*
NDM-Kpn-166	**$bla_{\text{NDM-1}}$**, $bla_{\text{CTX-M-15}}$, $bla_{\text{OXA-1}}$, $bla_{\text{OXA-9}}$, $bla_{\text{TEM-1A}}$, $bla_{\text{SHV-11}}$	*aph(3')-VI, aac(6')-Ib, ant(3'')-Ia, armA, aac(6')-Ib-cr, qacE, oqxA1, oqxB1, fosA5, mrs(E), mph(A), mph(E), catB3, qnrS1, ARR-3, sul1, sul2, dfrA5*
NDM-Kpn-167	**$bla_{\text{NDM-1}}$, $bla_{\text{VIM-1}}$**, $bla_{\text{CTX-M-15}}$, $bla_{\text{OXA-9}}$, $bla_{\text{TEM-1A}}$, $bla_{\text{SHV-12}}$	*aph(3')-VI, aac(6')-Ib, ant(3'')-Ia, aph(3')-XV, aac(6')-Ib-cr, qacE, oqxA1, oqxB1, fosA5, mph(A), catB2, qnrS1, ARR-3, sul1, dfrA14*
NDM-Kpn-168	**$bla_{\text{NDM-1}}$**, $bla_{\text{CTX-M-15}}$, $bla_{\text{OXA-1}}$, $bla_{\text{SHV-182}}$	*aac(6')-Ib, aac(6')-Ib-cr, aac(3)-IIa, oqxA1, oqxB1, fosA3, catB3, dfrA14*
NDM-Kpn-169	**$bla_{\text{NDM-1}}$**, $bla_{\text{CTX-M-15}}$, $bla_{\text{OXA-1}}$, $bla_{\text{SHV-182}}$	*aac(6')-Ib, aac(6')-Ib-cr, aac(3)-IIa, oqxA1, oqxB1, fosA3, catB3, dfrA14*
NDM-Kpn-170	**$bla_{\text{NDM-1}}$**, $bla_{\text{CTX-M-15}}$, $bla_{\text{OXA-1}}$, $bla_{\text{TEM-1B}}$, $bla_{\text{SHV-182}}$	*aph(6)-Id, aac(6')-Ib, aadA2, aph(3')-Ia, aac(6')-Ib-cr, aph(3'')-Ib, aac(3)-IIa, qacE, oqxA1, oqxB1, fosA3, mph(A), catB3, catA1, sul1, sul2, dfrA12, dfrA14*
NDM-Kpn-171	**$bla_{\text{NDM-1}}$, $bla_{\text{OXA-48}}$**, $bla_{\text{CTX-M-15}}$, $bla_{\text{SHV-11}}$	*aph(3')-VI, aadA2, armA, qacE, oqxA1, oqxB1, fosA5, mrs(E), mph(A), mph(E), sul1, dfrA12, tet(A)*
NDM-Kpn-172	**$bla_{\text{NDM-1}}$, $bla_{\text{OXA-48}}$**, $bla_{\text{CTX-M-15}}$, $bla_{\text{SHV-11}}$	*aac(6')-Ib, aac(6')-Ib-cr, aac(3)-IIa, oqxA1, oqxB1, fosA3, catB3, dfrA14*
NDM-Kpn-173	**$bla_{\text{NDM-1}}$, $bla_{\text{VIM-1}}$**, $bla_{\text{CTX-M-15}}$, $bla_{\text{OXA-9}}$, $bla_{\text{TEM-1A}}$, $bla_{\text{SHV-12}}$	*aph(3')-VI, aac(6')-Ib, aac(6')-Ib-cr, ant(3'')-Ia, aph(3')-XV, qacE, oqxA1, oqxB1, fosA5, mph(A), catB2, qnrS1, ARR-3, sul1, dfrA14*

NDM-Kpn-174	***bla***$_{\mathbf{NDM-1}}$, ***bla***$_{\mathbf{OXA-48}}$, *bla*$_{CTX-M-15}$, *bla*$_{SHV-11}$	*aph(3')-VI, aadA2, armA, qacE, oqxA1, oqxB1, fosA5, mrs(E), mph(A), mph(E), sul1, dfrA12, tet(A)*
NDM-Kpn-175	***bla***$_{\mathbf{NDM-5}}$, ***bla***$_{\mathbf{KPC-3}}$, *bla*$_{OXA-9}$, *bla*$_{TEM-1A}$, *bla*$_{SHV-182}$	*aadA2, aph(3')-Ia, qacE, oqxA1, oqxB1, fosA3, mph(A), catA1, sul1, dfrA12*
NDM-Kpn-176	***bla***$_{\mathbf{NDM-1}}$, ***bla***$_{\mathbf{OXA-48}}$, *bla*$_{CTX-M-15}$, *bla*$_{SHV-11}$	*aph(3')-VI, aadA2, armA, qacE, oqxA1, oqxB1, fosA5, mrs(E), mph(A), mph(E), sul1, dfrA12, tet(A)*
NDM-Kpn-177	***bla***$_{\mathbf{NDM-1}}$, ***bla***$_{\mathbf{OXA-48}}$, *bla*$_{CTX-M-15}$, *bla*$_{SHV-67}$	*aph(3')-VI, aadA2, armA, qacE, oqxA1, oqxB1, fosA5, mrs(E), mph(A), mph(E), sul1, dfrA12, tet(A)*
NDM-Kpn-178	***bla***$_{\mathbf{NDM-1}}$, ***bla***$_{\mathbf{OXA-48}}$, *bla*$_{CTX-M-15}$, *bla*$_{SHV-11}$	*aph(3')-VI, aadA2, armA, qacE, oqxA1, oqxB1, fosA5, mrs(E), mph(A), mph(E), sul1, dfrA12, tet(A)*
NDM-Kpn-179	***bla***$_{\mathbf{NDM-1}}$, ***bla***$_{\mathbf{OXA-48}}$, *bla*$_{CTX-M-15}$, *bla*$_{SHV-11}$	*aph(3')-VI, aadA2, armA, qacE, oqxA1, oqxB1, fosA5, mrs(E), mph(A), mph(E), sul1, dfrA12, tet(A)*
NDM-Kpn-180	***bla***$_{\mathbf{NDM-1}}$, ***bla***$_{\mathbf{OXA-48}}$, *bla*$_{CTX-M-15}$, *bla*$_{SHV-67}$	*aph(3')-VI, aadA2, armA, qacE, oqxA1, oqxB1, fosA5, mrs(E), mph(A), mph(E), sul1, dfrA12, tet(A)*
NDM-Kpn-181	***bla***$_{\mathbf{NDM-1}}$, ***bla***$_{\mathbf{VIM-1}}$, *bla*$_{CTX-M-15}$, *bla*$_{OXA-9}$, *bla*$_{TEM-1A}$, *bla*$_{SHV-12}$	*aph(3')-VI, aac(6')-Ib, ant(3'')-Ia, aph(3')-XV, aac(6')-Ib-cr, qacE, oqxA1, oqxB1, fosA5, mph(A), catB2, qnrS1, ARR-3, sul1, dfrA14*
NDM-Kpn-182	***bla***$_{\mathbf{NDM-1}}$, *bla*$_{CTX-M-15}$, *bla*$_{OXA-9}$, *bla*$_{TEM-1A}$, *bla*$_{SHV-11}$	*aph(3')-VI, aac(6')-Ib, aadA1, aac(6')-Ib-cr, qacE, oqxA1, oqxB1, fosA5, qnrS1, ARR-3, sul1*
NDM-Kpn-183	***bla***$_{\mathbf{NDM-1}}$, *bla*$_{SHV-27}$, *bla*$_{SHV-1}$	*aph(3')-VI, armA, qacE, oqxA1, oqxB1, fosA3, mrs(E), mph(A), mph(E), qnrS1, sul1, sul2, tet(D), dfrA5*
NDM-Kpn-184	***bla***$_{\mathbf{NDM-5}}$, *bla*$_{OXA-10}$, *bla*$_{SHV-55}$, *bla*$_{SHV-1}$	*aac(6')-II, aph(3')-VI, qacE, oqxA1, oqxB1, fosA3, qnrS1, sul1*
NDM-Kpn-185	***bla***$_{\mathbf{NDM-1}}$, *bla*$_{CTX-M-15}$, *bla*$_{OXA-1}$, *bla*$_{SHV-182}$	*aac(6')-Ib, aac(6')-Ib-cr, aac(3)-IIa, oqxA1, oqxB1, fosA3, catB3, dfrA14*
NDM-Kpn-186	***bla***$_{\mathbf{NDM-1}}$, *bla*$_{CTX-M-15}$, *bla*$_{OXA-1}$, *bla*$_{SHV-11}$	*aac(6')-Ib, aac(6')-Ib-cr, aac(3)-IIa, oqxA1, oqxB1, fosA3, catB3, dfrA14*
NDM-Kpn-187	***bla***$_{\mathbf{NDM-1}}$, ***bla***$_{\mathbf{VIM-1}}$, *bla*$_{CTX-M-15}$, *bla*$_{OXA-9}$, *bla*$_{TEM-1A}$, *bla*$_{SHV-12}$	*aph(3')-VI, aac(6')-Ib, ant(3'')-Ia, aph(3')-XV, aac(6')-Ib-cr, qacE, oqxA1, oqxB1, fosA5, mph(A), catB2, qnrS1, ARR-3, sul1, dfrA14*
NDM-Kpn-188	***bla***$_{\mathbf{NDM-1}}$, ***bla***$_{\mathbf{VIM-1}}$, *bla*$_{CTX-M-15}$, *bla*$_{OXA-9}$, *bla*$_{TEM-1A}$, *bla*$_{SHV-12}$	*aph(3')-VI, ant(3'')-Ia, aph(3')-XV, aac(6')-Ib-cr, qacE, oqxA1, oqxB1, fosA5, mph(A), catB2, qnrS1, ARR-3, sul1, dfrA14*
NDM-Kpn-189	***bla***$_{\mathbf{NDM-1}}$, ***bla***$_{\mathbf{VIM-1}}$, *bla*$_{CTX-M-15}$, *bla*$_{OXA-9}$, *bla*$_{TEM-1A}$, *bla*$_{SHV-12}$	*aph(3')-VI, aac(6')-Ib, ant(3'')-Ia, aph(3')-XV, aac(6')-Ib-cr, qacE, oqxA1, oqxB1, fosA5, mph(A), catB2, qnrS1, ARR-3, sul1, dfrA14*
NDM-Kpn-190	***bla***$_{\mathbf{NDM-5}}$, *bla*$_{CTX-M-15}$, *bla*$_{OXA-9}$, *bla*$_{OXA-1}$, *bla*$_{TEM-1A}$, *bla*$_{SHV-11}$	*aph(3')-VI, aac(6')-Ib, ant(3'')-Ia, aph(3')-Ia, armA, aac(6')-Ib-cr, qacE, oqxA1, oqxB1, fosA5, mrs(E), mph(A), mph(E), catB3, qnrS1, ARR-3, sul1, sul2, dfrA5*
NDM-Kpn-191	***bla***$_{\mathbf{NDM-1}}$, ***bla***$_{\mathbf{OXA-48}}$, *bla*$_{CTX-M-15}$, *bla*$_{SHV-11}$	*aph(3')-VI, aadA2, armA, qacE, oqxA1, fosA5, mrs(E), mph(A), mph(E), sul1, tet(A), dfrA12*
NDM-Kpn-192	***bla***$_{\mathbf{NDM-1}}$, ***bla***$_{\mathbf{OXA-48}}$, *bla*$_{CTX-M-15}$, *bla*$_{SHV-67}$	*aph(3')-VI, aadA2, armA, qacE, oqxA1, oqxB1, fosA5, mrs(E), mph(A), mph(E), sul1, tet(A), dfrA12*
NDM-Kpn-193	***bla***$_{\mathbf{NDM-1}}$, *bla*$_{CTX-M-15}$, *bla*$_{OXA-9}$, *bla*$_{OXA-1}$, *bla*$_{TEM-1A}$, *bla*$_{SHV-67}$	*aph(3')-VI, aac(6')-Ib, ant(3'')-Ia, armA, aac(6')-Ib-cr, qacE, oqxA1, oqxB1, fosA5, mrs(E), mph(A), mph(E), catB3, qnrS1, ARR-3, sul1, sul2, dfrA5*
NDM-Kpn-194	***bla***$_{\mathbf{NDM-1}}$, *bla*$_{CTX-M-15}$, *bla*$_{OXA-1}$, *bla*$_{SHV-182}$	*aac(6')-Ib, aac(6')-Ib-cr, aac(3)-IIa, oqxA1, oqxB1, fosA3, catB3, dfrA14*
NDM-Kpn-195	***bla***$_{\mathbf{NDM-1}}$, *bla*$_{CTX-M-15}$, *bla*$_{OXA-1}$, *bla*$_{SHV-182}$	*aac(6')-Ib, aac(6')-Ib-cr, aac(3)-IIa, oqxA1, oqxB1, fosA3, catB3, dfrA14*
NDM-Kpn-196	***bla***$_{\mathbf{NDM-1}}$, ***bla***$_{\mathbf{OXA-48}}$, *bla*$_{CTX-M-15}$, *bla*$_{SHV-67}$	*aph(3')-VI, aadA2, armA, qacE, oqxA1, oqxB1, fosA5, mrs(E), mph(A), mph(E), sul1, tet(A), dfrA12*
NDM-Kpn-197	***bla***$_{\mathbf{NDM-1}}$, *bla*$_{CTX-M-15}$, *bla*$_{OXA-1}$, *bla*$_{SHV-182}$	*aac(6')-Ib, aac(6')-Ib-cr, aac(3)-IIa, oqxA1, oqxB1, fosA3, catB3, dfrA14*
NDM-Kpn-198	***bla***$_{\mathbf{NDM-1}}$, *bla*$_{CTX-M-15}$, *bla*$_{OXA-1}$, *bla*$_{SHV-182}$	*aac(6')-Ib, aac(6')-Ib-cr, aac(3)-IIa, oqxA1, oqxB1, fosA3, catB3, dfrA14*

NDM-Kpn-199	***bla***$_{\textbf{NDM-1}}$, *bla*$_{\text{CTX-M-15}}$, *bla*$_{\text{OXA-1}}$, *bla*$_{\text{SHV-182}}$	*aac(6')-Ib, aac(6')-Ib-cr, aac(3)-IIa, oqxA1, oqxB1, fosA3, catB3, dfrA14*
NDM-Kpn-200	***bla***$_{\textbf{NDM-1}}$, ***bla***$_{\textbf{VIM-1}}$, *bla*$_{\text{CTX-M-15}}$, *bla*$_{\text{OXA-9}}$, *bla*$_{\text{TEM-1A}}$, *bla*$_{\text{SHV-12}}$	*aph(3')-VI, aac(6')-Ib, ant(3'')-Ia, aph(3')-XV, aac(6')-Ib-cr, qacE, oqxA1, oqxB1, fosA5, mph(A), catB2, qnrS1, ARR-3, sul1, dfrA14*
NDM-Kpn-201	***bla***$_{\textbf{NDM-1}}$, ***bla***$_{\textbf{OXA-48}}$, *bla*$_{\text{CTX-M-15}}$, *bla*$_{\text{SHV-11}}$	*aph(3')-VI, aadA2, armA, qacE, oqxA1, oqxB1, fosA5, mrs(E), mph(A), mph(E), sul1, tet(A), dfrA12*
NDM-Kpn-202	***bla***$_{\textbf{NDM-1}}$, *bla*$_{\text{CTX-M-15}}$, *bla*$_{\text{OXA-1}}$, *bla*$_{\text{SHV-158}}$	*aac(6')-Ib, aac(6')-Ib-cr, aac(3)-IIa, oqxA1, oqxB1, fosA3, catB3, dfrA14*
NDM-Kpn-203	***bla***$_{\textbf{NDM-1}}$, ***bla***$_{\textbf{OXA-48}}$, *bla*$_{\text{CTX-M-15}}$, *bla*$_{\text{SHV-11}}$	*aph(3')-VI, aadA2, armA, qacE, oqxA1, oqxB1, fosA5, mrs(E), mph(A), mph(E), sul1, tet(A), dfrA12*
NDM-Kpn-204	***bla***$_{\textbf{NDM-1}}$, ***bla***$_{\textbf{OXA-48}}$, *bla*$_{\text{CTX-M-15}}$, *bla*$_{\text{SHV-67}}$	*aph(3')-VI, aadA2, armA, qacE, oqxA1, oqxB1, fosA5, mrs(E), mph(A), mph(E), sul1, tet(A), dfrA12*
NDM-Kpn-205	***bla***$_{\textbf{NDM-1}}$, ***bla***$_{\textbf{OXA-48}}$, *bla*$_{\text{CTX-M-15}}$, *bla*$_{\text{SHV-11}}$	*aph(3')-VI, aadA2, armA, qacE, oqxA1, oqxB1, fosA5, mrs(E), mph(A), mph(E), sul1, tet(A), dfrA12*
NDM-Kpn-206	***bla***$_{\textbf{NDM-1}}$, ***bla***$_{\textbf{OXA-48}}$, *bla*$_{\text{CTX-M-15}}$, *bla*$_{\text{SHV-67}}$	*aph(3')-VI, aadA2, armA, qacE, oqxA1, oqxB1, fosA5, mrs(E), mph(A), mph(E), sul1, tet(A), dfrA12*
NDM-Kpn-207	***bla***$_{\textbf{NDM-1}}$, *bla*$_{\text{CTX-M-15}}$, *bla*$_{\text{OXA-1}}$, *bla*$_{\text{SHV-182}}$	*aac(6')-Ib, aac(6')-Ib-cr, aac(3)-IIa, oqxA1, oqxB1, fosA3, catB3, dfrA14*
NDM-Kpn-208	***bla***$_{\textbf{NDM-1}}$, ***bla***$_{\textbf{VIM-1}}$, *bla*$_{\text{CTX-M-15}}$, *bla*$_{\text{OXA-9}}$, *bla*$_{\text{TEM-1A}}$, *bla*$_{\text{SHV-12}}$	*aph(3')-VI, aac(6')-Ib, ant(3'')-Ia, aph(3')-XV, aac(6')-Ib-cr, qacE, oqxA1, oqxB1, fosA5, mph(A), catB2, qnrS1, ARR-3, sul1, dfrA14*
NDM-Kpn-209	***bla***$_{\textbf{NDM-1}}$, ***bla***$_{\textbf{OXA-48}}$, *bla*$_{\text{CTX-M-15}}$, *bla*$_{\text{SHV-11}}$	*aph(3')-VI, aadA2, armA, qacE, oqxA1, oqxB1, fosA5, mrs(E), mph(A), mph(E), sul1, tet(A), dfrA12*
NDM-Kpn-210	***bla***$_{\textbf{NDM-1}}$, ***bla***$_{\textbf{OXA-48}}$, *bla*$_{\text{CTX-M-15}}$, *bla*$_{\text{SHV-11}}$	*aph(3')-VI, aadA2, armA, qacE, oqxA1, oqxB1, fosA5, mrs(E), mph(A), mph(E), sul1, tet(A), dfrA12*
NDM-Kpn-211	***bla***$_{\textbf{NDM-1}}$, ***bla***$_{\textbf{VIM-1}}$, *bla*$_{\text{CTX-M-15}}$, *bla*$_{\text{OXA-9}}$, *bla*$_{\text{TEM-1A}}$, *bla*$_{\text{SHV-12}}$	*aph(3')-VI, aac(6')-Ib, ant(3'')-Ia, aph(3')-XV, aac(6')-Ib-cr, qacE, oqxA1, oqxB1, fosA5, mph(A), catB2, qnrS1, ARR-3, sul1, dfrA14*
NDM-Kpn-212	***bla***$_{\textbf{NDM-1}}$, ***bla***$_{\textbf{OXA-48}}$, *bla*$_{\text{CTX-M-15}}$, *bla*$_{\text{SHV-11}}$	*aph(3')-VI, aadA2, armA, qacE, oqxA1, oqxB1, fosA5, mrs(E), mph(A), mph(E), sul1, tet(A), dfrA12*
NDM-Kpn-213	***bla***$_{\textbf{NDM-1}}$, ***bla***$_{\textbf{OXA-48}}$, *bla*$_{\text{CTX-M-15}}$, *bla*$_{\text{SHV-11}}$	*aph(3')-VI, aadA2, armA, qacE, oqxA1, oqxB1, fosA5, mrs(E), mph(A), mph(E), sul1, tet(A), dfrA12*
NDM-Kpn-214	***bla***$_{\textbf{NDM-1}}$, ***bla***$_{\textbf{OXA-48}}$, *bla*$_{\text{CTX-M-15}}$, *bla*$_{\text{SHV-11}}$	*aph(3')-VI, aadA2, armA, qacE, oqxA1, oqxB1, fosA5, mrs(E), mph(A), mph(E), sul1, tet(A), dfrA12*
NDM-Kpn-215	***bla***$_{\textbf{NDM-1}}$, ***bla***$_{\textbf{OXA-48}}$, *bla*$_{\text{CTX-M-15}}$, *bla*$_{\text{SHV-11}}$	*aph(3')-VI, aadA2, armA, qacE, oqxA1, oqxB1, fosA5, mrs(E), mph(A), mph(E), sul1, tet(A), dfrA12*
NDM-Kpn-216	***bla***$_{\textbf{NDM-1}}$, ***bla***$_{\textbf{OXA-48}}$, *bla*$_{\text{CTX-M-15}}$, *bla*$_{\text{SHV-182}}$	*aac(6')-Ib, aac(6')-Ib-cr, aac(3)-IIa, oqxA1, oqxB1, fosA3, catB3, dfrA14*
NDM-Kpn-217	***bla***$_{\textbf{NDM-1}}$, ***bla***$_{\textbf{OXA-48}}$, *bla*$_{\text{CTX-M-15}}$, *bla*$_{\text{SHV-11}}$	*aph(3')-VI, aadA2, armA, qacE, oqxA1, oqxB1, fosA5, mrs(E), mph(A), mph(E), sul1, tet(A), dfrA12*
NDM-Kpn-218	***bla***$_{\textbf{NDM-1}}$, ***bla***$_{\textbf{OXA-48}}$, *bla*$_{\text{CTX-M-15}}$, *bla*$_{\text{SHV-182}}$	*aph(3')-VI, aac(6')-Ib, aadA2, armA, aac(6')-Ib-cr, aac(3)-IIa, qacE, oqxA1, oqxB1, fosA3, mrs(E), mph(A), mph(E), catB3, tet(A), dfrA12, dfrA14*
NDM-Kpn-219	***bla***$_{\textbf{NDM-1}}$, ***bla***$_{\textbf{OXA-48}}$, *bla*$_{\text{CTX-M-15}}$, *bla*$_{\text{OXA-395}}$, *bla*$_{\text{PAO}}$, *bla*$_{\text{SHV-11}}$	*aph(3')-VI, aph(3')-IIb, aadA2, armA, qacE, oqxA1, oqxB1, fosA4, fosA5, mrs(E), mph(A), mph(E), catB7, crpP, sul1, tet(A), dfrA12*
NDM-Kpn-220	***bla***$_{\textbf{NDM-1}}$, *bla*$_{\text{CTX-M-15}}$, *bla*$_{\text{OXA-1}}$, *bla*$_{\text{OXA-9}}$, *bla*$_{\text{TEM-1A}}$, *bla*$_{\text{SHV-11}}$	*aph(3')-VI, aac(6')-Ib, ant(3'')-Ia, armA, aac(6')-Ib-cr, qacE, oqxA1, oqxB1, fosA5, mrs(E), mph(A), mph(E), catB3, qnrS1, ARR-3, sul1, sul2 dfrA5*
NDM-Kpn-221	***bla***$_{\textbf{NDM-5}}$, ***bla***$_{\textbf{OXA-48}}$, *bla*$_{\text{CTX-M-14b}}$, *bla*$_{\text{CTX-M-15}}$, *bla*$_{\text{OXA-1}}$, *bla*$_{\text{TEM-1A}}$, *bla*$_{\text{SHV-11}}$	*aph(6)-Id, aph(3')-VI, aph(3')-Ia, aph(3')-VIb, armA, aac(6')-Ib-cr, aph(3'')-Ib, qacE, oqxA1, oqxB1, fosA5, mrs(E), mph(A), mph(E), catB3, qnrS1, ARR-3, sul1, sul2 dfrA5*
NDM-Kpn-222	***bla***$_{\textbf{NDM-1}}$, ***bla***$_{\textbf{KPC-3}}$, *bla*$_{\text{CTX-M-15}}$, *bla*$_{\text{OXA-1}}$, *bla*$_{\text{OXA-9}}$, *bla*$_{\text{TEM-1B}}$, *bla*$_{\text{SHV-28}}$	*aph(6)-Id, aph(3')-VI, aac(6')-Ib-cr, aadA1, aph(3'')-Ib, aac(3)-IIa, oqxA, oqxB, fosA6, catB3, qnrB1, qnrS1, ARR-3, sul1, sul2, dfrA14*
NDM-Kpn-223	***bla***$_{\textbf{NDM-1}}$, *bla*$_{\text{CTX-M-15}}$, *bla*$_{\text{OXA-1}}$, *bla*$_{\text{OXA-9}}$, *bla*$_{\text{TEM-1A}}$, *bla*$_{\text{SHV-67}}$	*aph(6)-Id, aph(3')-VI, aac(6')-Ib,ant(3'')-Ia, armA, aac(6')-Ib-cr, aph(3'')-Ib, qacE, oqxA1, oqxB1, fosA3, fosA5, mrs(E), mph(A), mph(E), catB3, qnrS1, ARR-3, sul1, sul2 dfrA5*

NDM-Kpn-224	***bla***$_{\text{NDM-1}}$**,** ***bla***$_{\text{OXA-48}}$, *bla*$_{\text{CTX-M-15}}$, *bla*$_{\text{SHV-67}}$	*aph(3')-VI, aadA2, armA, qacE, oqxA1, oqxB1, fosA5, mrs(E), mph(A), mph(E), sul1, tet(A), dfrA12*
NDM-Kpn-225	***bla***$_{\text{NDM-1}}$**,** *bla*$_{\text{CTX-M-15}}$, *bla*$_{\text{SHV-11}}$	*aph(3')-VI, aadA2, armA, qacE, oqxA1, fosA5, mrs(E), mph(A), mph(E), sul1, tet(A), dfrA12*
NDM-Kpn-226	***bla***$_{\text{NDM-1}}$**,** *bla*$_{\text{CTX-M-15}}$, *bla*$_{\text{OXA-1}}$, *bla*$_{\text{SHV-182}}$	*aac(6')-Ib, aac(6')-Ib-cr, aac(3)-IIa, oqxA1, oqxB1, fosA3, catB3, dfrA14*
NDM-Kpn-227	***bla***$_{\text{NDM-1}}$**,** *bla*$_{\text{CTX-M-15}}$, *bla*$_{\text{OXA-1}}$, *bla*$_{\text{SHV-182}}$	*aac(6')-Ib, aac(6')-Ib-cr, aac(3)-IIa, oqxA1, oqxB1, fosA3, catB3, dfrA14*
NDM-Kpn-228	***bla***$_{\text{NDM-1}}$**,** *bla*$_{\text{CTX-M-15}}$, *bla*$_{\text{OXA-1}}$, *bla*$_{\text{SHV-182}}$	*aac(6')-Ib, aac(6')-Ib-cr, aac(3)-IIa, oqxA1, oqxB1, fosA3, catB3, dfrA14*
NDM-Kpn-229	***bla***$_{\text{NDM-1}}$, ***bla***$_{\text{VIM-1}}$, *bla*$_{\text{CTX-M-15}}$, *bla*$_{\text{OXA-9}}$, *bla*$_{\text{TEM-1A}}$, *bla*$_{\text{SHV-11}}$	*aph(3')-VI, aac(6')-Ib, ant(3'')-Ia, aph(3')-XV, aac(6')-Ib-cr, qacE, oqxA1, oqxB1, fosA5, mph(A), catB2, qnrS1, ARR-3, sul1, dfrA14*
NDM-Kpn-230	***bla***$_{\text{NDM-1}}$, *bla*$_{\text{CTX-M-15}}$, *bla*$_{\text{OXA-1}}$, *bla*$_{\text{OXA-9,}}$ *bla*$_{\text{TEM-1A,}}$ *bla*$_{\text{SHV-67}}$	*aph(3')-VI, aac(6')-Ib, aadA1, aac(6')-Ib-cr, qacE, oqxA1, oqxB1, fosA5, mph(A), catB3, qnrS1, ARR-3, sul1*
NDM-Kpn-231	***bla***$_{\text{NDM-1,}}$***bla***$_{\text{OXA-48}}$, *bla*$_{\text{CTX-M-15}}$, *bla*$_{\text{SHV-11}}$	*aph(3')-VI, aadA2, armA, qacE, oqxA1, oqxB1, fosA5, mrs(E), mph(A), mph(E), sul1, tet(A), dfrA12*
NDM-Kpn-232	***bla***$_{\text{NDM-1}}$, ***bla***$_{\text{OXA-48}}$, *bla*$_{\text{CTX-M-15}}$, *bla*$_{\text{SHV-11}}$	*aph(3')-VI, aadA2, armA, qacE, oqxA1, oqxB1, fosA5, mrs(E), mph(A), mph(E), sul1, tet(A), dfrA12*
NDM-Kpn-233	***bla***$_{\text{NDM-1}}$**,** ***bla***$_{\text{OXA-48}}$, *bla*$_{\text{CTX-M-15}}$, *bla*$_{\text{SHV-67}}$	*aph(3')-VI, aadA2, armA, qacE, oqxA1, oqxB1, fosA5, mrs(E), mph(A), mph(E), sul1, tet(A), dfrA12*
NDM-Kpn-234	***bla***$_{\text{NDM-1}}$**,** ***bla***$_{\text{OXA-48}}$, *bla*$_{\text{CTX-M-15}}$, *bla*$_{\text{SHV-67}}$	*aph(3')-VI, aadA2, armA, qacE, oqxA1, oqxB1, fosA5, mrs(E), mph(A), mph(E), sul1, tet(A), dfrA12*
NDM-Kpn-235	***bla***$_{\text{NDM-1}}$**,** ***bla***$_{\text{OXA-48}}$, *bla*$_{\text{CTX-M-15}}$, *bla*$_{\text{SHV-11}}$	*aph(3')-VI, aadA2, armA, qacE, oqxA1, oqxB1, fosA5, mrs(E), mph(A), mph(E), sul1, tet(A), dfrA12*
NDM-Kpn-236	***bla***$_{\text{NDM-1}}$**,** ***bla***$_{\text{OXA-48}}$, *bla*$_{\text{CTX-M-15}}$, *bla*$_{\text{SHV-67}}$	*aph(3')-VI, aadA2, armA, qacE, oqxA1, oqxB1, fosA5, mrs(E), mph(A), mph(E), sul1, tet(A), dfrA12*
NDM-Kpn-237	***bla***$_{\text{NDM-1}}$**,** ***bla***$_{\text{OXA-48}}$, *bla*$_{\text{CTX-M-15}}$, *bla*$_{\text{SHV-11}}$	*aph(3')-VI, aadA2, armA, qacE, oqxA1, oqxB1, fosA5, mrs(E), mph(A), mph(E), sul1, tet(A), dfrA12*
NDM-Kpn-238	***bla***$_{\text{NDM-1}}$**,** ***bla***$_{\text{OXA-48}}$, *bla*$_{\text{CTX-M-15}}$, *bla*$_{\text{SHV-11}}$	*aph(3')-VI, aadA2, armA, qacE, oqxA1, oqxB1, fosA5, mrs(E), mph(A), mph(E), sul1, tet(A), dfrA12*
NDM-Kpn-239	***bla***$_{\text{NDM-1}}$**,** ***bla***$_{\text{OXA-48}}$, *bla*$_{\text{CTX-M-15}}$, *bla*$_{\text{SHV-11}}$	*aph(3')-VI, aadA2, armA, qacE, oqxA1, oqxB1, fosA5, mrs(E), mph(A), mph(E), sul1, tet(A), dfrA12*
NDM-Kpn-240	***bla***$_{\text{NDM-1}}$**,** ***bla***$_{\text{OXA-48}}$, *bla*$_{\text{CTX-M-15}}$, *bla*$_{\text{SHV-11}}$	*aph(3')-VI, aadA2, armA, qacE, oqxA1, oqxB1, fosA5, mrs(E), mph(A), mph(E), sul1, tet(A), dfrA12*
NDM-Kpn-241	***bla***$_{\text{NDM-1}}$**,** ***bla***$_{\text{OXA-48}}$, *bla*$_{\text{CTX-M-15}}$, *bla*$_{\text{SHV-182}}$	*aac(6')-Ib, aac(6')-Ib-cr, aac(3)-IIa, oqxA1, oqxB1, fosA_3, catB3, dfrA14*
NDM-Kpn-242	***bla***$_{\text{NDM-1}}$**,** ***bla***$_{\text{OXA-48}}$, *bla*$_{\text{CTX-M-15}}$, *bla*$_{\text{SHV-11}}$	*aph(3')-VI, aadA2, armA, qacE, oqxA1, oqxB1, fosA5, mrs(E), mph(A), mph(E), sul1, tet(A), dfrA12*
NDM-Kpn-243	***bla***$_{\text{NDM-1}}$, *bla*$_{\text{CTX-M-15,}}$ *bla*$_{\text{OXA-1}}$, *bla*$_{\text{SHV-182}}$	*aac(6')-Ib, aac(6')-Ib-cr, oqxA1, oqxB1, fosA3, catB3, dfrA14*
NDM-Kpn-244	***bla***$_{\text{NDM-1}}$**,** ***bla***$_{\text{OXA-48}}$, *bla*$_{\text{CTX-M-15,}}$ *bla*$_{\text{SHV-67}}$	*aph(3')-VI, aadA2, armA, qacE, oqxA1, oqxB1, fosA5, mrs(E), mph(A), mph(E), sul1, tet(A), dfrA12*
NDM-Kpn-245	***bla***$_{\text{NDM-1}}$**,** *bla*$_{\text{CTX-M-15,}}$ *bla*$_{\text{OXA-1}}$, *bla*$_{\text{OXA-9}}$, *bla*$_{\text{TEM-1C}}$, *bla*$_{\text{SHV-11}}$	*aph(6)-Id, aph(3')-VI, aac(6')-Ib, ant(3'')-Ia, aac(6')-Ib-cr, aph(3'')-Ib, aac(3)-IIa, qacE, oqxA1, oqxB1, fosA5, catB3, qnrB1, qnrS1, ARR-3, sul1, sul2, tet(A), dfrA14*
NDM-Kpn-246	***bla***$_{\text{NDM-1}}$**,** ***bla***$_{\text{KPC-3}}$, *bla*$_{\text{CTX-M-15,}}$ *bla*$_{\text{OXA-1}}$, *bla*$_{\text{OXA-9}}$, *bla*$_{\text{TEM-1A,}}$ *bla*$_{\text{SHV-11}}$	*aac(6')-Ib, aac(6')-Ib-cr, oqxA1, oqxB1, fosA3, catB3, dfrA14*
NDM-Kpn-247	***bla***$_{\text{NDM-1}}$**,** *bla*$_{\text{CTX-M-15,}}$ *bla*$_{OXA\text{-}1}$, *bla*$_{\text{TEM-1B}}$, *bla*$_{\text{SHV-182}}$	*aph(6)-Id, aac(6')-Ib, aadA2, aph(3')-Ia, aac(6')-Ib-cr, aph(3'')-Ib, aac(3)-IIa, qacE, oqxA1, oqxB1, fosA3, mph(A), catB3, catA1, sul1, sul2, dfrA12, dfrA14*
NDM-Kpn-248	***bla***$_{\text{NDM-5}}$**,** ***bla***$_{\text{OXA-48}}$**,** *bla*$_{\text{CTX-M-15,}}$ *bla*$_{\text{OXA-1}}$, *bla*$_{\text{SHV-11}}$	*aph(3')-VI, aph(3')-Ia, armA, aac(6')-Ib-cr, qacE, oqxA1, oqxB1, fosA5, mrs(E), mph(A), mph(E), catB3, qnrS1, ARR-3, sul1, sul2, dfrA5*

NDM-Kpn-249	***bla***$_{\text{NDM-1}}$, *bla*$_{\text{CTX-M-15}}$, *bla*$_{\text{OXA-1}}$, *bla*$_{\text{OXA-9}}$, *bla*$_{\text{TEM-1A}}$, *bla*$_{\text{SHV-11}}$	*aph(3')-VI, aac(6')-Ib, ant(3'')-Ia, aac(6')-Ib-cr, qacE, oqxA1, oqxB1, fosA5, mrs(E), mph(A), mph(E), catB3, qnrS1, ARR-3, sul1, sul2, dfrA5*
NDM-Kpn-250	***bla***$_{\text{NDM-1}}$, *bla*$_{\text{CTX-M-15}}$, *bla*$_{\text{OXA-1}}$, *bla*$_{\text{SHV-182}}$	*aac(6')-Ib, aac(6')-Ib-cr, aac(3)-IIa, oqxA1, oqxB1, fosA3, catB3, dfrA14*
NDM-Kpn-251	***bla***$_{\text{NDM-1}}$, *bla*$_{\text{CTX-M-15}}$, *bla*$_{\text{OXA-1}}$, *bla*$_{\text{SHV-182}}$	*aac(6')-Ib, aac(6')-Ib-cr, oqxA1, oqxB1, fosA3, catB3, dfrA14*
NDM-Kpn-252	***bla***$_{\text{NDM-1}}$, *bla*$_{\text{CTX-M-15}}$, *bla*$_{\text{OXA-1}}$, *bla*$_{\text{OXA-9}}$, *bla*$_{\text{TEM-1A}}$, *bla*$_{\text{SHV-182}}$	*aac(6')-Ib, aac(6')-Ib-cr, oqxA1, oqxB1, fosA3, catB3, dfrA14*
NDM-Kpn-253	***bla***$_{\text{NDM-1}}$, *bla*$_{\text{CTX-M-15}}$, *bla*$_{\text{OXA-1}}$, *bla*$_{\text{SHV-182}}$	*aac(6')-Ib, aac(6')-Ib-cr, oqxA1, oqxB1, fosA3, catB3, dfrA14*
NDM-Kpn-254	***bla***$_{\text{NDM-1}}$, ***bla***$_{\text{OXA-48}}$, *bla*$_{\text{CTX-M-15}}$, *bla*$_{\text{SHV-11}}$	*aph(3')-VI, aadA2, armA, qacE, oqxA1, oqxB1, fosA5, mrs(E), mph(A), mph(E), sul1, tet(A), dfrA12*
NDM-Kpn-255	***bla***$_{\text{NDM-1}}$, ***bla***$_{\text{OXA-48}}$, *bla*$_{\text{CTX-M-15}}$, *bla*$_{\text{SHV-11}}$	*aph(3')-VI, aadA2, armA, qacE, oqxA1, oqxB1, fosA5, mrs(E), mph(A), mph(E), sul1, tet(A), dfrA12*
NDM-Kpn-256	***bla***$_{\text{NDM-1}}$, ***bla***$_{\text{OXA-48}}$, *bla*$_{\text{CTX-M-15}}$, *bla*$_{\text{SHV-11}}$	*aph(3')-VI, aadA2, armA, qacE, oqxA1, fosA5, mrs(E), mph(A), mph(E), sul1, tet(A), dfrA12*
NDM-Kpn-257	***bla***$_{\text{NDM-1}}$, ***bla***$_{\text{OXA-48}}$, *bla*$_{\text{CTX-M-15}}$, *bla*$_{\text{SHV-11}}$	*aph(3')-VI, aadA2, armA, qacE, oqxA1, fos5, mrs(E), mph(A), mph(E), sul1, tet(A), dfrA12*
NDM-Kpn-258	***bla***$_{\text{NDM-1}}$, ***bla***$_{\text{OXA-48}}$, *bla*$_{\text{CTX-M-15}}$, *bla*$_{\text{SHV-67}}$	*aph(3')-VI, aadA2, armA, qacE, oqxA1, oqxB1, fosA5, mrs(E), mph(A), mph(E), sul1, tet(A), dfrA12*
NDM-Kpn-259	***bla***$_{\text{NDM-1}}$, ***bla***$_{\text{OXA-48}}$, *bla*$_{\text{CTX-M-15}}$, *bla*$_{\text{SHV-67}}$	*aph(3')-VI, aadA2, armA, qacE, oqxA1, oqxB1, fosA5, mrs(E), mph(A), mph(E), sul1, tet(A), dfrA12*
NDM-Kpn-260	***bla***$_{\text{NDM-1}}$, *bla*$_{\text{CTX-M-15}}$, *bla*$_{\text{OXA-1}}$, *bla*$_{\text{OXA-9}}$, *bla*$_{\text{TEM-1A}}$, *bla*$_{\text{SHV-11}}$	*aph(3')-VI, aac(6')-Ib, aadA1, aac(6')-Ib-cr, qacE, oqxA1, oqxB1, fosA5, catB3, qnrS1, ARR-3, sul1*
NDM-Kpn-261	***bla***$_{\text{NDM-1}}$, *bla*$_{\text{CTX-M-15}}$, *bla*$_{\text{OXA-1}}$, *bla*$_{\text{SHV-1}}$, *bla*$_{\text{SHV-155}}$	*aac(6')-Ib, aac(6')-Ib-cr, oqxA1, oqxB1, fosA3, catB3, dfrA14*
NDM-Kpn-262	***bla***$_{\text{NDM-1}}$, *bla*$_{\text{CTX-M-15}}$, *bla*$_{\text{OXA-1}}$, *bla*$_{\text{SHV-1}}$	*aac(6')-Ib, aac(6')-Ib-cr, oqxA1, oqxB1, fosA3, catB3, dfrA14*
NDM-Kpn-263	***bla***$_{\text{NDM-1}}$, *bla*$_{\text{CTX-M-15}}$, *bla*$_{\text{OXA-1}}$, *bla*$_{\text{SHV-1}}$	*aac(6')-Ib, aac(6')-Ib-cr, oqxA1, oqxB1, fosA3, catB3, dfrA14*
NDM-Kpn-264	***bla***$_{\text{NDM-1}}$, *bla*$_{\text{CTX-M-15}}$, *bla*$_{\text{OXA-1}}$, *bla*$_{\text{TEM-1B}}$, *bla*$_{\text{SHV-182}}$	*aph(6)-Id, aac(6')-Ib, aadA2, aph(3')-Ia, aac(6')-Ib-cr, aph(3'')-Ib, aac(3)-IIa, qacE, oqxA1, oqxB1, fosA3, mph(A), catB3, catA1, sul1, sul2, dfrA12, dfrA14*
NDM-Kpn-265	***bla***$_{\text{NDM-1}}$, ***bla***$_{\text{OXA-48}}$, *bla*$_{\text{CTX-M-15}}$, *bla*$_{\text{SHV-11}}$	*aph(3')-VI, aadA2, armA, qacE, oqxA1, oqxB1, fosA5, mrs(E), mph(A), mph(E), sul1, tet(A), dfrA12*
NDM-Kpn-266	***bla***$_{\text{NDM-1}}$, *bla*$_{\text{CTX-M-15}}$, *bla*$_{\text{CMY-4}}$, *bla*$_{\text{OXA-1}}$, *bla*$_{\text{OXA-9}}$, *bla*$_{\text{OXA-10}}$, *bla*$_{\text{TEM-1B}}$, *bla*$_{\text{TEM-92}}$, *bla*$_{\text{SHV-182}}$	*aph(6)-Id, aph(3')-Ia, aac(2')-Ia, aac(3)-IIa, aac(6')-Ib, ant(3'')-Ia, aph(3')-VIb, aac(6')-Ib-cr, aph(3'')-Ib, qacE, oqxA1, oqxB1, fosA3, catB3, catA3, cmlA1, floR_2, qnrB1, ARR-2, sul1, sul2, tet(A), tet(B), dfrA14*
NDM-Kpn-267	***bla***$_{\text{NDM-1}}$, *bla*$_{\text{CTX-M-15}}$, *bla*$_{\text{OXA-1}}$, *bla*$_{\text{SHV-182}}$	*aac(6')-Ib, aac(6')-Ib-cr, oqxA1, oqxB1, fosA3, catB3, dfrA14*
NDM-Kpn-268	***bla***$_{\text{NDM-1}}$, *bla*$_{\text{CTX-M-15}}$, *bla*$_{\text{OXA-1}}$, *bla*$_{\text{TEM-1B}}$, *bla*$_{\text{SHV-182}}$	*aph(6)-Id, aac(6')-Ib, aadA2, aph(3')-Ia, aac(6')-Ib-cr, aph(3'')-Ib, aac(3)-IIa, qacE, oqxA1, oqxB1, fosA3, mph(A), catB3, catA1, sul1, sul2, dfrA12, dfrA14*
NDM-Kpn-269	***bla***$_{\text{NDM-5}}$, ***bla***$_{\text{OXA-48}}$, *bla*$_{\text{CTX-M-15}}$, *bla*$_{\text{OXA-1}}$, *bla*$_{\text{SHV-11}}$	*aph(3')-VI, aph(3')-Ia, armA, aac(6')-Ib-cr, qacE, oqxA1, oqxB1, fosA5, mrs(E), mph(A), mph(E), catB3, qnrS1, ARR-3, sul1, sul2, dfrA5*
NDM-Kpn-270	***bla***$_{\text{NDM-1}}$, ***bla***$_{\text{OXA-48}}$, *bla*$_{\text{CTX-M-15}}$, *bla*$_{\text{SHV-67}}$	*aph(3')-VI, aadA2, armA, qacE, oqxA1, oqxB1, fosA5, mrs(E), mph(A), mph(E), sul1, tet(A), dfrA12*
NDM-Kpn-271	***bla***$_{\text{NDM-1}}$, *bla*$_{\text{CTX-M-15}}$, *bla*$_{\text{OXA-1}}$, *bla*$_{\text{OXA-9}}$, *bla*$_{\text{TEM-1A}}$, *bla*$_{\text{SHV-11}}$	*aph(3')-VI, aac(6')-Ib, ant(3'')-Ia, armA, aac(6')-Ib-cr, qacE, oqxA1, oqxB1, fosA5, mrs(E), mph(A), mph(E), catB3, qnrS1, ARR-3, sul1, sul2, dfrA5*
NDM-Kpn-272	***bla***$_{\text{NDM-1}}$, *bla*$_{\text{CTX-M-15}}$, *bla*$_{\text{OXA-1}}$, *bla*$_{\text{OXA-9}}$, *bla*$_{\text{TEM-1A}}$, *bla*$_{\text{SHV-11}}$	*aph(3')-VI, aac(6')-Ib, ant(3'')-Ia, armA, aac(6')-Ib-cr, qacE, oqxA1, oqxB1, fosA5, mrs(E), mph(A), mph(E), catB3, qnrS1, ARR-3, sul1, sul2, dfrA5*
NDM-Kpn-273	***bla***$_{\text{NDM-1}}$, *bla*$_{\text{CTX-M-15}}$, *bla*$_{\text{OXA-1}}$, *bla*$_{\text{SHV-182}}$	*aac(6')-Ib, aac(6')-Ib-cr, oqxA1, oqxB1, fosA3, catB3, dfrA14*

NDM-Kpn-274	***bla***$_{\mathbf{NDM-1}}$, ***bla***$_{\mathbf{OXA-48}}$, *bla*$_{CTX-M-15}$, *bla*$_{SHV-11}$	*aph(3')-VI, aadA2, armA, qacE, oqxA1, oqxB1, fosA5, mrs(E), mph(A), mph(E), sul1, tet(A), dfrA12*
NDM-Kpn-275	***bla***$_{\mathbf{NDM-1}}$, *bla*$_{CTX-M-15}$, *bla*$_{OXA-1}$, *bla*$_{SHV-182}$	*aac(6')-Ib, aac(6')-Ib-cr, aac(3)-IIa, oqxA1, oqxB1, fosA3, catB3, dfrA14*
NDM-Kpn-276	***bla***$_{\mathbf{NDM-1}}$, *bla*$_{CTX-M-15}$, *bla*$_{OXA-1}$, *bla*$_{OXA-9}$, *bla*$_{TEM-1A}$, *bla*$_{SHV-11}$	*aph(3')-VI, aac(6')-Ib, ant(3'')-Ia, armA, aac(6')-Ib-cr, qacE, oqxA1, oqxB1, fosA5, mrs(E), mph(A), mph(E), catB3, qnrS1, ARR-3, sul1, sul2, dfrA5*
NDM-Kpn-277	***bla***$_{\mathbf{NDM-1}}$, *bla*$_{CTX-M-15}$, *bla*$_{OXA-1}$, *bla*$_{OXA-9}$, *bla*$_{TEM-1A}$, *bla*$_{SHV-67}$	*aph(3')-VI, aac(6')-Ib, ant(3'')-Ia, aac(6')-Ib-cr, qacE, oqxA1, oqxB1, fosA5, catB3, qnrS1, ARR-3, sul1*
NDM-Kpn-278	***bla***$_{\mathbf{NDM-1}}$, *bla*$_{CTX-M-15}$, *bla*$_{OXA-1}$, *bla*$_{OXA-9}$, *bla*$_{TEM-1A}$, *bla*$_{SHV-11}$	*aph(3')-VI, aac(6')-Ib, ant(3'')-Ia, aac(6')-Ib-cr, qacE, oqxA1, oqxB1, fosA5, catB3, qnrS1, ARR-3, sul1*
NDM-Kpn-279	***bla***$_{\mathbf{NDM-1}}$, *bla*$_{CTX-M-15}$, *bla*$_{OXA-1}$, *bla*$_{TEM-1B}$, *bla*$_{SHV-182}$	*aph(6)-Id, aac(6')-Ib, aadA2, aph(3')-Ia, aac(6')-Ib-cr, aph(3'')-Ib, aac(3)-IIa, qacE, oqxA1, oqxB1, fosA3, mph(A), catB3, catA1, sul1, sul2, dfrA12, dfrA14*
NDM-Kpn-280	***bla***$_{\mathbf{NDM-1}}$, ***bla***$_{\mathbf{OXA-48}}$, *bla*$_{CTX-M-15}$, *bla*$_{SHV-11}$	*aph(3')-VI, aadA2, armA, qacE, oqxA1, oqxB1, fosA5, mrs(E), mph(A), mph(E), sul1, tet(A), dfrA12*
NDM-Kpn-281	***bla***$_{\mathbf{NDM-1}}$, *bla*$_{CTX-M-15}$, *bla*$_{OXA-1}$, *bla*$_{SHV-182}$	*aac(6')-Ib, aac(6')-Ib-cr, aac(3)-IIa, oqxA1, oqxB1, fosA3, catB3, dfrA14*
NDM-Kpn-282	***bla***$_{\mathbf{NDM-1}}$, *bla*$_{SHV-27}$	*aph(3')-VI, armA, qacE, oqxA1, oqxB1, fosA3, mrs(E), mph(A), mph(E), qnrS1, sul1, sul2, dfrA5*
NDM-Kpn-283	***bla***$_{\mathbf{NDM-1}}$, *bla*$_{CTX-M-15}$, *bla*$_{OXA-1}$, *bla*$_{OXA-9}$, *bla*$_{TEM-1A}$, *bla*$_{SHV-11}$	*aph(3')-VI, aac(6')-Ib, ant(3'')-Ia, armA, aac(6')-Ib-cr, qacE, oqxA1, oqxB1, fosA5, mrs(E), mph(A), mph(E), catB3, qnrS1, ARR-3, sul1, sul2, dfrA5*
NDM-Kpn-284	***bla***$_{\mathbf{NDM-1}}$, *bla*$_{CTX-M-15}$, *bla*$_{OXA-1}$, *bla*$_{OXA-9}$, *bla*$_{TEM-1A}$, *bla*$_{SHV-67}$	*aph(3')-VI, aac(6')-Ib, ant(3'')-Ia, armA, aac(6')-Ib-cr, qacE, oqxA1, oqxB1, fosA5, mrs(E), mph(A), mph(E), catB3, qnrS1, ARR-3, sul1, sul2, dfrA5*
NDM-Kpn-285	***bla***$_{\mathbf{NDM-1}}$, *bla*$_{CTX-M-15}$, *bla*$_{OXA-1}$, *bla*$_{OXA-9}$, *bla*$_{TEM-1A}$, *bla*$_{SHV-67}$	*aph(3')-VI, aac(6')-Ib, ant(3'')-Ia, armA, aac(6')-Ib-cr, qacE, oqxA1, oqxB1, fosA5, mrs(E), mph(A), mph(E), catB3, qnrS1, ARR-3, sul1, sul2, dfrA5*
NDM-Kpn-286	***bla***$_{\mathbf{NDM-1}}$, *bla*$_{CTX-M-15}$, *bla*$_{OXA-10}$, *bla*$_{SHV-185}$	*aph(6)-Id, ant(2'')-Ia, ant(3'')-Ia, aadA2, aph(3')-Ia, armA, aph(3'')-Ib, qacE, oqxA1, oqxB1, fosA3, mrs(E), mph(A), mph(E), cmlA1, qnrS1, ARR-2, sul1, sul2, tet(A), dfrA12*
NDM-Kpn-287	***bla***$_{\mathbf{NDM-1}}$, *bla*$_{CTX-M-15}$, *bla*$_{OXA-1}$, *bla*$_{OXA-9}$, *bla*$_{TEM-1A}$, *bla*$_{SHV-11}$	*aph(3')-VI, aac(6')-Ib, ant(3'')-Ia, armA, aac(6')-Ib-cr, qacE, oqxA1, oqxB1, fosA3, fosA5, mrs(E), mph(A), mph(E), catB3, qnrS1, ARR-3, sul1, sul2, dfrA5*
NDM-Kpn-288	***bla***$_{\mathbf{NDM-1}}$, ***bla***$_{\mathbf{OXA-48}}$, *bla*$_{CTX-M-15}$, *bla*$_{SHV-67}$	*aph(3')-VI, aadA2, armA, qacE, oqxA1, oqxB1, fosA5, mrs(E), mph(A), mph(E), sul1, tet(A), dfrA12*
NDM-Kpn-289	***bla***$_{\mathbf{NDM-1}}$, *bla*$_{CTX-M-15}$, *bla*$_{OXA-1}$, *bla*$_{OXA-9}$, *bla*$_{TEM-1A}$	*aac(6')-Ib, aac(6')-Ib-cr, oqxA1, oqxB1, fosA3, catB3, dfrA14*
NDM-Kpn-290	***bla***$_{\mathbf{NDM-5}}$, *bla*$_{CTX-M-15}$, *bla*$_{CMY-2}$, *bla*$_{OXA-9}$, *bla*$_{TEM-1A}$, *bla*$_{SHV-11}$	*aph(6)-Id, aph(3')-VI, aac(3')-IId, aac(6')-Ib, aadA1, aph(3'')-Ib, qacE, oqxA1, oqxB1, fosA5, mph(A), floR_2, catA1, qnrS1, sul1, sul2, tet(A), dfrA5*
NDM-Kpn-291	***bla***$_{\mathbf{NDM-1}}$, *bla*$_{CTX-M-15}$, *bla*$_{OXA-1}$, *bla*$_{TEM-1B}$, *bla*$_{SHV-182}$	*aph(6)-Id, aac(6')-Ib, aadA2, aph(3')-Ia, aac(6')-Ib-cr, aph(3'')-Ib, aac(3)-IIa, qacE, oqxA1, oqxB1, fosA3, mph(A), catB3, catA1, sul1, sul2, dfrA12, dfrA14*
NDM-Kpn-292	***bla***$_{\mathbf{NDM-1}}$, *bla*$_{CTX-M-15}$, *bla*$_{OXA-1}$, *bla*$_{TEM-1A}$, *bla*$_{SHV-12}$	*ant(2'')-Ia, aac(6')-Ib, aph(3')-Ia, aac(6')-Ib-cr, aac(3)-IIa, oqxA1, oqxB1, fosA3, catB3, qnrB19, dfrA14*
NDM-Kpn-293	***bla***$_{\mathbf{NDM-1}}$, ***bla***$_{\mathbf{OXA-48}}$, *bla*$_{CTX-M-15}$, *bla*$_{SHV-67}$	*aph(3')-VI, aadA2, armA, qacE, oqxA1, oqxB1, fosA5, mrs(E), mph(A), mph(E), sul1, tet(A), dfrA12*
NDM-Kpn-294	***bla***$_{\mathbf{NDM-1}}$, ***bla***$_{\mathbf{OXA-48}}$, *bla*$_{CTX-M-15}$, *bla*$_{SHV-11}$	*aph(3')-VI, aadA2, armA, qacE, oqxA1, oqxB1, fosA5, mrs(E), mph(A), mph(E), sul1, tet(A), dfrA12*
NDM-Kpn-295	***bla***$_{\mathbf{NDM-1}}$, ***bla***$_{\mathbf{OXA-48}}$, *bla*$_{CTX-M-15}$, *bla*$_{SHV-67}$	*aph(3')-VI, aadA2, armA, qacE, oqxA1, oqxB1, fosA5, mrs(E), mph(A), mph(E), sul1, tet(A), dfrA12*
NDM-Kpn-296	***bla***$_{\mathbf{NDM-1}}$, ***bla***$_{\mathbf{OXA-48}}$, *bla*$_{CTX-M-15}$, *bla*$_{SHV-11}$	*aph(3')-VI, aadA2, armA, qacE, oqxA1, oqxB1, fosA5, mrs(E), mph(A), mph(E), sul1, tet(A), dfrA12*
NDM-Kpn-297	***bla***$_{\mathbf{NDM-5}}$, *bla*$_{OXA-181}$, *bla*$_{CTX-M-15}$, *bla*$_{OXA-1}$, *bla*$_{TEM-1B}$, *bla*$_{SHV-148}$	*aph(6)-Id, aadA2,aac(6')-Ib-cr, aph(3'')-Ib, aac(3)-IIa, qacE, oqxA1, oqxB1, mph(A), catB3, qnrS1, sul1, sul2, dfrA12, dfrA14*
NDM-Kpn-298	***bla***$_{\mathbf{NDM-1}}$, ***bla***$_{\mathbf{OXA-48}}$, *bla*$_{CTX-M-15}$, *bla*$_{SHV-11}$	*aph(3')-VI, aadA2, armA, qacE, oqxA1, oqxB1, fosA5, mrs(E), mph(A), mph(E), sul1, tet(A), dfrA12*

NDM-Kpn-299	***bla*****NDM-1, *bla*****OXA-48,** *bla*CTX-M-15, *bla*SHV-11	*aph(3')-VI, aadA2, armA, qacE, oqxA1, oqxB1, fosA5, mrs(E), mph(A), mph(E), sul1, tet(A), dfrA12*
NDM-Kpn-300	***bla*****NDM-1, *bla*****OXA-48**, *bla*CTX-M-15, *bla*SHV-11	*aph(3')-VI, aadA2, armA, qacE, oqxA1, oqxB1, fosA5, mrs(E), mph(A), mph(E), sul1, tet(A), dfrA12*
NDM-Kpn-301	***bla*****NDM-1**, *bla*CTX-M-15, *bla*OXA-9, *bla*TEM-1A, *bla*SHV-11	*aph(3')-VI, aac(6')-Ib, aadA1, aac(6')-Ib-cr, qacE, oqxA1, oqxB1, fosA5, qnrS1, ARR-3, sul1*
NDM-Kpn-302	***bla*****NDM-1,** *bla*CTX-M-15, *bla*OXA-1, *bla*OXA-9, *bla*TEM-1A, *bla*SHV-11	*aph(3')-VI, aac(6')-Ib, ant(3'')-Ia, armA, aac(6')-Ib-cr, qacE, oqxA1, oqxB1, fosA5, mrs(E), mph(A), mph(E), catB3, qnrS1, ARR-3, sul1, sul2, dfrA5*
NDM-Kpn-303	***bla*****NDM-5, *bla*****OXA-48**, *bla*CTX-M-15, *bla*OXA-9, *bla*TEM-1C, *bla*SHV-148	*aph(6)-Id, aph(3')-VI, aac(6')-Ib, ant(3'')-Ia, aph(3')-Ia, armA, aph(3'')-Ib, qacE, oqxA1, oqxB1, fosA5, mrs(E), mph (A), mph(E), catA1, qnrS1, sul1, sul2, tet(A), dfrA5*
NDM-Kpn-304	***bla*****NDM-1**, *bla*CTX-M-15, *bla*OXA-1, *bla*OXA-9, *bla*TEM-1A, *bla*SHV-67	*aph(3')-VI, aac(6')-Ib, ant(3'')-Ia, armA, aac(6')-Ib-cr, qacE, oqxA1, oqxB1, fosA5, mrs(E), mph(A), mph(E), catB3, qnrS1, ARR-3, sul1, sul2, dfrA5*
NDM-Kpn-305	***bla*****NDM-1, *bla*****OXA-48,** *bla*CTX-M-15, *bla*SHV-11	*aph(3')-VI, aadA2, armA, qacE, oqxA1, fosA5, mrs(E), mph(A), mph(E), sul1, tet(A), dfrA12*
NDM-Kpn-306	***bla*****NDM-5, *bla*****OXA-48**, *bla*CTX-M-15, *bla*OXA-9, *bla*TEM-1C, *bla*SHV-148	*aph(6)-Id, aph(3')-VI, aac(6')-Ib, ant(3'')-Ia, aph(3')-Ia, armA, aph(3'')-Ib, qacE, oqxA1, oqxB1, fosA5, mrs(E), mph (A), mph(E), catA1, qnrS1, sul1, sul2, tet(A), dfrA5*
NDM-Kpn-307	***bla*****NDM-1**, *bla*CTX-M-15, *bla*OXA-1, *bla*OXA-9, *bla*TEM-1A, *bla*SHV-67	*aph(3')-VI, aac(6')-Ib, ant(3'')-Ia, armA, aac(6')-Ib-cr, qacE, oqxA1, oqxB1, fosA5, mrs(E), mph (A), mph(E), catB3, qnrS1, ARR-3, sul1, sul2, tet(A), dfrA5*
NDM-Kpn-308	***bla*****NDM-1, *bla*****OXA-48**, *bla*KPC-2, *bla*CTX-M-14b, *bla*CTX-M-65, *bla*CTX-M-15, *bla*SHV-182	*aph(6)-Id, aph(3')-VI, aph(3')-Ia, armA, aph(3'')-Ib, qacE, oqxA1, oqxB1, fosA3, mrs(E), mph (A), mph(E), qnrS1, sul1, sul2, dfrA5*
NDM-Kpn-309	***bla*****NDM-1**, *bla*CTX-M-15, *bla*OXA-1, *bla*OXA-9, *bla*TEM-1A, *bla*SHV-67	*aph(3')-VI, aac(6')-Ib, ant(3'')-Ia, armA, aac(6')-Ib-cr, qacE, oqxA1, oqxB1, fosA5, mrs(E), mph(A), mph(E), catB3, qnrS1, ARR-3, sul1, sul2, dfrA5*
NDM-Kpn-310	***bla*****NDM-1, *bla*****OXA-48**, *bla*CTX-M-15, *bla*SHV-11	*aph(3')-VI, aadA2, armA, qacE, oqxA1, oqxB1, fosA5, mrs(E), mph(A), mph(E), sul1, tet(A), dfrA12*
NDM-Kpn-311	***bla*****NDM-1,** *bla*CTX-M-15, *bla*OXA-1, *bla*SHV-182	*aph(3')-VI, aac(6')-Ib, aadA2, armA, aac(6')-Ib-cr, qacE, oqxA1, oqxB1, fosA3, mrs(E), mph(E), catB3, sul1, dfrA12, dfrA14*
NDM-Kpn-312	***bla*****NDM-1**, *bla*CTX-M-15, *bla*CMY-4, *bla*OXA-1, *bla*OXA-10, *bla*SHV-182	*aph(6)-Id, aph(3')-Ia, aac(6')-Ib, ant(3'')-Ia, aph(3')-VIb, aac(6')-Ib-cr, aph(3'')-Ib, aac(3)-IIa, qacE, oqxA1, oqxB1, fosA3, catB3, cmlA1, floR_2, ARR-2, sul1, sul2, tet(A), tet(B), dfrA14*
NDM-Kpn-313	***bla*****NDM-1, *bla*****OXA-48**, *bla*CTX-M-15, *bla*SHV-11	*aph(3')-VI, aadA2, armA, qacE, oqxA1, oqxB1, fosA5, mrs(E), mph(A), mph(E), sul1, tet(A), dfrA12*
NDM-Kpn-314	***bla*****NDM-1, *bla*****VIM-1,** *bla*CTX-M-15, *bla*OXA-9, *bla*TEM-1A, *bla*SHV-11	*aph(3')-VI, aac(6')-Ib, ant(3'')-Ia, aph(3')-XV, aac(6')-Ib-cr, qacE, oqxA1, oqxB1, fosA5, mph(A), catB2, qnrS1, ARR-3, sul1, dfrA14*
NDM-Kpn-315	***bla*****NDM-1, *bla*****OXA-48**, *bla*CTX-M-15, *bla*SHV-11	*aph(3')-VI, aadA2, armA, qacE, oqxA1, oqxB1, fosA5, mrs(E), mph(A), mph(E), sul1, tet(A), dfrA12*
NDM-Kpn-316	***bla*****NDM-1, *bla*****OXA-48**, *bla*CTX-M-15, *bla*SHV-67	*aph(3')-VI, aadA2, armA, qacE, oqxA1, oqxB1, fosA5, mrs(E), mph(A), mph(E), sul1, tet(A), dfrA12*
NDM-Kpn-317	***bla*****NDM-1**, *bla*CTX-M-15, *bla*SHV-11	*aph(3')-VI, aadA2, armA, qacE, oqxA1, oqxB1, fosA5, mrs(E), mph(A), mph(E), sul1, tet(A), dfrA12*
NDM-Kpn-318	***bla*****NDM-1, *bla*****OXA-48**, *bla*CTX-M-15, *bla*SHV-11	*aph(3')-VI, aadA2, armA, qacE, oqxA1, oqxB1, fosA5, mrs(E), mph(A), mph(E), sul1, tet(A), dfrA12*
NDM-Kpn-319	***bla*****NDM-1, *bla*****OXA-48**, *bla*CTX-M-15, *bla*SHV-11	*aph(3')-VI, aadA2, armA, qacE, oqxA1, oqxB1, fosA5, mrs(E), mph(A), mph(E), sul1, tet(A), dfrA12*
NDM-Kpn-320	***bla*****NDM-1, *bla*****OXA-48**, *bla*CTX-M-15, *bla*SHV-11	*aph(3')-VI, aadA2, armA, qacE, oqxA1, oqxB1, fosA5, mrs(E), mph(A), mph(E), sul1, tet(A), dfrA12*
NDM-Kpn-321	***bla*****NDM-1**, *bla*CTX-M-15, *bla*OXA-1, *bla*SHV-158	*aac(6')-Ib, aac(6')-Ib-cr, aac(3)-IIa, oqxA1, oqxB1, fosA3, catB3, dfrA14*
NDM-Kpn-322	***bla*****NDM-1, *bla*****OXA-48**, *bla*CTX-M-15, *bla*SHV-11	*aph(3')-VI, aadA2, armA, qacE, oqxA1, oqxB1, fosA5, mrs(E), mph(A), mph(E), sul1, tet(A), dfrA12*
NDM-Kpn-323	***bla*****NDM-1**, *bla*CTX-M-15, *bla*OXA-1, *bla*SHV-182	*aac(6')-Ib, aac(6')-Ib-cr, aac(3)-IIa, oqxA1, fosA3, catB3, dfrA14*

NDM-Kpn-324	***bla***$_{NDM-1}$, ***bla***$_{OXA-48}$, *bla*$_{CTX-M-15}$, *bla*$_{SHV-67}$	*aph(3')-VI, aadA2, armA, qacE, oqxA1, oqxB1, fosA5, mrs(E), mph(A), mph(E), sul1, tet(A), dfrA12*
NDM-Kpn-325	***bla***$_{NDM-1}$, *bla*$_{CTX-M-15}$, *bla*$_{OXA-1}$, *bla*$_{SHV-182}$	*aac(6')-Ib, aac(6')-Ib-cr, oqxA1, oqxB1, fosA3, catB3, dfrA14*
NDM-Kpn-326	***bla***$_{NDM-1}$, ***bla***$_{OXA-48}$, *bla*$_{CTX-M-15}$, *bla*$_{SHV-11}$	*aph(3')-VI, aadA2, armA, qacE, oqxA1, oqxB1, fosA5, mrs(E), mph(A), mph(E), sul1, tet(A), dfrA12*
NDM-Kpn-327	***bla***$_{NDM-1}$, *bla*$_{CTX-M-15}$, *bla*$_{OXA-1}$, *bla*$_{OXA-9}$, *bla*$_{TEM-1A}$, *bla*$_{SHV-67}$	*aph(3')-VI, aac(6')-Ib, ant(3'')-Ia, aac(6')-Ib-cr, qacE, oqxA1, oqxB1, fosA5, mrs(E), mph(A), mph(E), catB3, qnrS1, ARR-3, sul1, sul2, dfrA5*
NDM-Kpn-328	***bla***$_{NDM-1}$, *bla*$_{CTX-M-15}$, *bla*$_{OXA-1}$, *bla*$_{OXA-9}$, *bla*$_{TEM-1A}$, *bla*$_{SHV-182}$	*aac(6')-Ib, aac(6')-Ib-cr, oqxA1, oqxB1, fosA3, catB3, dfrA14*
NDM-Kpn-329	***bla***$_{NDM-1}$, ***bla***$_{OXA-48}$, *bla*$_{CTX-M-15}$, *bla*$_{SHV-11}$	*aph(3')-VI, aadA2, aph(3')-Ia, armA, qacE, oqxA1, oqxB1, fosA5, mrs(E), mph(A), mph(E), sul1, tet(A), dfrA5, dfrA12*
NDM-Kpn-330	***bla***$_{NDM-1}$, *bla*$_{CTX-M-15}$, *bla*$_{SHV-11}$	*aph(3')-VI, aadA2, armA, qacE, oqxA1, oqxB1, fosA5, mrs(E), mph(A), mph(E), sul1, tet(A), dfrA12*
NDM-Kpn-331	***bla***$_{NDM-1}$, ***bla***$_{OXA-48}$, *bla*$_{CTX-M-15}$, *bla*$_{SHV-11}$	*aph(3')-VI, aadA2, armA, qacE, oqxA1, oqxB1, fosA5, mrs(E), mph(A), mph(E), sul1, tet(A), dfrA12*
NDM-Kpn-332	***bla***$_{NDM-5}$, ***bla***$_{OXA-48}$, *bla*$_{CTX-M-15}$, *bla*$_{OXA-1}$, *bla*$_{SHV-11}$	*aph(3')-VI, aph(3')-Ia, armA, aac(6')-Ib-cr, qacE, oqxA1, oqxB1, fosA5, mrs(E), mph(A), mph(E), catB3, qnrS1, ARR-3, sul1, sul2, dfrA5*
NDM-Kpn-333	***bla***$_{NDM-1}$, *bla*$_{CTX-M-15}$, *bla*$_{OXA-1}$, *bla*$_{OXA-9}$, *bla*$_{TEM-1A}$, *bla*$_{SHV-67}$	*aph(3')-VI, aac(6')-Ib, aadA1, aac(6')-Ib-cr, qacE, oqxA1, oqxB1, fosA5, catB3, qnrS1, ARR-3, sul1*
NDM-Kpn-334	***bla***$_{NDM-1}$, *bla*$_{CTX-M-15}$, *bla*$_{OXA-1}$, *bla*$_{OXA-9}$, *bla*$_{TEM-1C}$, *bla*$_{SHV-11}$	*aph(6)-Id, aph(3')-VI, aac(6')-Ib, ant(3'')-Ia, aac(6')-Ib-cr, aph(3'')-Ib, aac(3)-IIa, qacE, oqxA1, oqxB1, fosA5, catB3, qnrB1, qnrS1, ARR-3, sul1, sul2, tet(A), dfrA14*
NDM-Kpn-335	***bla***$_{NDM-1}$, ***bla***$_{VIM-1}$, *bla*$_{CTX-M-15}$, *bla*$_{SHV-12}$	*aph(3')-VI, ant(3'')-Ia, aph(3')-XV, aac(6')-Ib-cr, qacE, oqxA1, oqxB1, fosA5, mph(A), catB2, qnrS1, ARR-3, sul1, dfrA14*
NDM-Kpn-336	***bla***$_{NDM-1}$, ***bla***$_{OXA-48}$, *bla*$_{CTX-M-15}$, *bla*$_{SHV-67}$	*aph(3')-VI, aadA2, armA, qacE, oqxA1, oqxB1, fosA5, mrs(E), mph(A), mph(E), sul1, tet(A), dfrA12*
NDM-Kpn-337	***bla***$_{NDM-1}$, *bla*$_{SHV-187}$	*aph(3')-VI, aadA2, armA, qacE, fosA3, mrs(E), mph(E), sul1, dfrA12*
NDM-Kpn-338	***bla***$_{NDM-1}$, *bla*$_{SHV-187}$	*aph(3')-VI, aadA2, armA, qacE, fosA3, mrs(E), mph(E), sul1, dfrA12*
NDM-Kpn-339	***bla***$_{NDM-1}$, ***bla***$_{OXA-48}$, *bla*$_{CTX-M-15}$, *bla*$_{SHV-67}$	*aph(3')-VI, aadA2, armA, qacE, oqxA1, oqxB1, fosA5, mrs(E), mph(A), mph(E), sul1, tet(A), dfrA12*
NDM-Kpn-340	***bla***$_{NDM-1}$, ***bla***$_{OXA-48}$, *bla*$_{CTX-M-15}$, *bla*$_{SHV-67}$	*aph(3')-VI, aadA2, armA, qacE, oqxA1, oqxB1, fosA5, mrs(E), mph(A), mph(E), sul1, tet(A), dfrA12*
NDM-Kpn-341	***bla***$_{NDM-1}$, ***bla***$_{OXA-48}$, *bla*$_{CTX-M-15}$, *bla*$_{SHV-11}$	*aph(3')-VI, aadA2, armA, qacE, oqxA1, oqxB1, fosA5, mrs(E), mph(A), mph(E), sul1, tet(A), dfrA12*
NDM-Kpn-342	***bla***$_{NDM-1}$, ***bla***$_{OXA-48}$, *bla*$_{CTX-M-15}$, *bla*$_{SHV-11}$	*aph(3')-VI, aadA2, armA, qacE, oqxA1, oqxB1, fosA5, mrs(E), mph(A), mph(E), sul1, tet(A), dfrA12*
NDM-Kpn-343	***bla***$_{NDM-1}$, ***bla***$_{OXA-48}$, *bla*$_{CTX-M-15}$, *bla*$_{SHV-67}$	*aph(3')-VI, aadA2, armA, qacE, oqxA1, oqxB1, fosA5, mrs(E), mph(A), mph(E), sul1, tet(A), dfrA12*
NDM-Kpn-344	***bla***$_{NDM-1}$, ***bla***$_{OXA-48}$, *bla*$_{CTX-M-15}$, *bla*$_{SHV-67}$	*aph(3')-VI, aadA2, armA, qacE, oqxA1, oqxB1, fosA5, mrs(E), mph(A), mph(E), sul1, tet(A), dfrA12*
NDM-Kpn-345	***bla***$_{NDM-1}$, *bla*$_{CTX-M-15}$, *bla*$_{OXA-1}$, *bla*$_{SHV-182}$	*aac(6')-Ib, aac(3)-IIa, oqxA1, oqxB1, fosA6, catB3, dfrA14*
NDM-Kpn-346	***bla***$_{NDM-1}$, *bla*$_{CTX-M-15}$, *bla*$_{OXA-1}$, *bla*$_{SHV-182}$	*aac(6')-Ib, aac(6')-Ib-cr, aac(3)-IIa, oqxA1, oqxB1, fosA3, catB3, dfrA14*
NDM-Kpn-347	***bla***$_{NDM-1}$, *bla*$_{CTX-M-15}$, *bla*$_{OXA-1}$, *bla*$_{SHV-182}$	*aac(6')-Ib, aac(6')-Ib-cr, aac(3)-IIa, oqxA1, oqxB1, fosA3, catB3, dfrA14*
NDM-Kpn-348	***bla***$_{NDM-5}$, ***bla***$_{OXA-48}$, *bla*$_{CTX-M-14b}$, *bla*$_{CTX-M-15}$, *bla*$_{OXA-1}$, *bla*$_{TEM-1A}$, *bla*$_{SHV-67}$	*aph(6)-Id, aph(3')-VI, aph(3')-Ia, aph(3')-VIb, armA, aac(6')-Ib-cr, aph(3'')-Ib, qacE, oqxA1, oqxB1, fosA5, mrs(E), mph(A), mph(E), catB3, qnrS1, ARR-3, sul1, sul2 dfrA5*

NDM-Kpn-349	$\boldsymbol{bla}_{\textbf{NDM-1}}$, $\boldsymbol{bla}_{\textbf{OXA-48}}$, $bla_{\text{CTX-M-15}}$, $bla_{\text{SHV-67}}$	*aph(3')-VI, aadA2, armA, qacE, oqxA1, oqxB1, fosA5, mrs(E), mph(A), mph(E), sul1, tet(A), dfrA12*
NDM-Kpn-350	$\boldsymbol{bla}_{\textbf{NDM-1}}$, $bla_{\text{CTX-M-15}}$, $bla_{\text{OXA-1}}$, $bla_{\text{OXA-9}}$, $bla_{\text{TEM-1A}}$, $bla_{\text{SHV-11}}$	*aph(3')-VI, aac(6')-Ib, aadA1, aac(6')-Ib-cr, qacE, oqxA1, oqxB1, fosA5, catB3, qnrS1, ARR-3, sul1*
NDM-Kpn-351	$\boldsymbol{bla}_{\textbf{NDM-1}}$, $bla_{\text{CTX-M-15}}$, $bla_{\text{OXA-1}}$, $bla_{\text{OXA-9}}$, $bla_{\text{TEM-1A}}$, $bla_{\text{SHV-11}}$	*aph(3')-VI, aac(6')-Ib, ant(3'')-Ia, armA, aac(6')-Ib-cr, qacE, oqxA1, oqxB1, fosA5, mrs(E), mph(A), mph(E), catB3, qnrS1, ARR-3, sul1, sul2, dfrA5*
NDM-Kpn-352	$\boldsymbol{bla}_{\textbf{NDM-1}}$, $\boldsymbol{bla}_{\textbf{KPC-3}}$, $bla_{\text{CTX-M-15}}$, $bla_{\text{OXA-1}}$, $bla_{\text{OXA-9}}$, $bla_{\text{TEM-57}}$, $bla_{\text{SHV-1}}$, $bla_{\text{SHV-182}}$	*aac(6')-Ib, aac(6')-Ib-cr, oqxA1, oqxB1, fosA3, catB3, dfrA14*
NDM-Kpn-353	$\boldsymbol{bla}_{\textbf{NDM-5}}$, $\boldsymbol{bla}_{\textbf{OXA-48}}$, $bla_{\text{CTX-M-15}}$, $bla_{\text{OXA-1}}$, $bla_{\text{SHV-11}}$	*aph(3')-VI, aph(3')-Ia, armA, aac(6')-Ib-cr, qacE, oqxA1, oqxB1, fosA5, mrs(E), mph(A), mph(E), catB3, qnrS1, ARR-3, sul1, sul2, dfrA5*
NDM-Kpn-354	$\boldsymbol{bla}_{\textbf{NDM-1}}$, $bla_{\text{SHV-27}}$, $bla_{\text{SHV-1}}$	*aph(3')-VI, armA, qacE, oqxA1, oqxB1, fosA3, mrs(E), mph(A), mph(E), qnrS1, sul1, sul2, tet(D), dfrA5*
NDM-Kpn-355	$\boldsymbol{bla}_{\textbf{NDM-1}}$, $bla_{\text{CTX-M-15}}$, $bla_{\text{OXA-1}}$, $bla_{\text{SHV-182}}$	*aac(6')-Ib, aac(6')-Ib-cr, oqxA1, oqxB1, fosA3, catB3, dfrA14*
NDM-Kpn-356	$\boldsymbol{bla}_{\textbf{NDM-1}}$, $bla_{\text{SHV-155}}$, $bla_{\text{SHV-1}}$	*aac(6')-Ib, oqxA1, oqxB1, fosA3, dfrA14*
NDM-Kpn-357	$\boldsymbol{bla}_{\textbf{NDM-1}}$, $bla_{\text{CTX-M-15}}$, $bla_{\text{OXA-1}}$, $bla_{\text{SHV-182}}$	*aac(6')-Ib, aac(6')-Ib-cr, oqxA1, oqxB1, fosA3, catB3, dfrA14*
NDM-Kpn-358	$\boldsymbol{bla}_{\textbf{NDM-5}}$, $\boldsymbol{bla}_{\textbf{OXA-48}}$, $bla_{\text{CTX-M-15}}$, $bla_{\text{SHV-11}}$	*aph(3')-VI, aadA2, armA, qacE, oqxA1, oqxB1, fosA5, mrs(E), mph(A), mph(E), sul1, tet(A), dfrA12*
NDM-Kpn-359	$\boldsymbol{bla}_{\textbf{NDM-1}}$, $bla_{\text{CTX-M-15}}$, $bla_{\text{OXA-1}}$, $bla_{\text{SHV-182}}$	*aac(6')-Ib, aac(3)-IIa, oqxA, oqxB, fosA6, catB3, dfrA14*
NDM-Kpn-360	$\boldsymbol{bla}_{\textbf{NDM-1}}$, $bla_{\text{CTX-M-15}}$, $bla_{\text{OXA-1}}$, $bla_{\text{OXA-9}}$, $bla_{\text{TEM-1A}}$, $bla_{\text{SHV-11}}$	*aph(3')-VI, aac(6')-Ib, ant(3'')-Ia, armA, aac(6')-Ib-cr, qacE, oqxA1, oqxB1, fosA5, mrs(E), mph(A), mph(E), catB3, qnrS1, ARR-3, sul1, sul2, dfrA5*
NDM-Kpn-361	$\boldsymbol{bla}_{\textbf{NDM-1}}$, $\boldsymbol{bla}_{\textbf{OXA-48}}$, $bla_{\text{CTX-M-15}}$, $bla_{\text{SHV-11}}$	*aph(3')-VI, aadA2, armA, qacE, oqxA1, oqxB1, fosA5, mrs(E), mph(A), mph(E), sul1, tet(A), dfrA12*
NDM-Kpn-362	$\boldsymbol{bla}_{\textbf{NDM-5}}$, $\boldsymbol{bla}_{\textbf{OXA-48}}$, $bla_{\text{CTX-M-15}}$, $bla_{\text{OXA-1}}$, $bla_{\text{SHV-11}}$, $bla_{\text{SHV-67}}$	*aph(3')-VI, aph(3')-Ia, armA, aac(6')-Ib-cr, oqxA1, oqxB1, fosA5, mrs(E), mph(A), mph(E), catB3, qnrS1, ARR-3, sul1, sul2, dfrA5*
NDM-Kpn-363	$\boldsymbol{bla}_{\textbf{NDM-1}}$, $bla_{\text{CTX-M-15}}$, $bla_{\text{OXA-1}}$, $bla_{\text{OXA-9}}$, $bla_{\text{TEM-1A}}$, $bla_{\text{SHV-182}}$	*aac(6')-Ib-cr, oqxA1, oqxB1, fosA6, catB3, dfrA14*
NDM-Kpn-364	$\boldsymbol{bla}_{\textbf{NDM-1}}$, $\boldsymbol{bla}_{\textbf{OXA-48}}$, $bla_{\text{CTX-M-15}}$, $bla_{\text{SHV-67}}$	*aph(3')-VI, aadA2, armA, qacE, oqxA1, oqxB1, fosA5, mrs(E), mph(A), mph(E), sul1, tet(A), dfrA12*
NDM-Kpn-365	$\boldsymbol{bla}_{\textbf{NDM-5}}$, $\boldsymbol{bla}_{\textbf{OXA-48}}$, $bla_{\text{CTX-M-15}}$, $bla_{\text{OXA-1}}$, $bla_{\text{SHV-11}}$	*aph(3')-VI, aph(3')-Ia, armA, aac(6')-Ib-cr, qacE, oqxA1, oqxB1, fosA5, mrs(E), mph(A), mph(E), catB3, qnrS1, ARR-3, sul1, sul2, dfrA5*
NDM-Kpn-366	$\boldsymbol{bla}_{\textbf{NDM-1}}$, $\boldsymbol{bla}_{\textbf{OXA-48}}$, $bla_{\text{CTX-M-15}}$, $bla_{\text{SHV-11}}$	*aph(3')-VI, aadA2, armA, qacE, oqxA1, oqxB1, fosA5, mrs(E), mph(A), mph(E), sul1, tet(A), dfrA12*
NDM-Kpn-367	$\boldsymbol{bla}_{\textbf{NDM-1}}$, $\boldsymbol{bla}_{\textbf{OXA-48}}$, $bla_{\text{CTX-M-15}}$, $bla_{\text{SHV-11}}$	*aph(3')-VI, aadA2, armA, qacE, oqxA1, oqxB1, fosA5, mrs(E), mph(A), mph(E), sul1, tet(A), dfrA12*
NDM-Kpn-368	$\boldsymbol{bla}_{\textbf{NDM-1}}$, $\boldsymbol{bla}_{\textbf{OXA-48}}$, $bla_{\text{CTX-M-15}}$, $bla_{\text{SHV-11}}$	*aph(3')-VI, aadA2, armA, qacE, oqxA1, oqxB1, fosA5, mrs(E), mph(A), mph(E), sul1, tet(A), dfrA12*
NDM-Kpn-369	$\boldsymbol{bla}_{\textbf{NDM-5}}$, $\boldsymbol{bla}_{\textbf{OXA-48}}$, $bla_{\text{CTX-M-15}}$, $bla_{\text{OXA-1}}$, $bla_{\text{SHV-81}}$	*aph(3')-VI, aph(3')-Ia, armA, aac(6')-Ib-cr, qacE, oqxA1, oqxB1, fosA5, mrs(E), mph(A), mph(E), catB3, qnrS1, ARR-3, sul1, sul2, dfrA5*
NDM-Kpn-370	$\boldsymbol{bla}_{\textbf{NDM-5}}$, $\boldsymbol{bla}_{\textbf{OXA-48}}$, $bla_{\text{CTX-M-15}}$, $bla_{\text{OXA-1}}$, $bla_{\text{SHV-11}}$	*aph(3')-VI, aph(3')-Ia, armA, aac(6')-Ib-cr, qacE, oqxA1, oqxB1, fosA5, mrs(E), mph(A), mph(E), catB3, qnrS1, ARR-3, sul1, sul2, dfrA5*
NDM-Kpn-371	$\boldsymbol{bla}_{\textbf{NDM-1}}$, $\boldsymbol{bla}_{\textbf{OXA-48}}$, $bla_{\text{CTX-M-15}}$, $bla_{\text{SHV-11}}$	*aph(3')-VI, aadA2, armA, qacE, oqxA1, oqxB1, fosA5, mrs(E), mph(A), mph(E), sul1, tet(A), dfrA12*
NDM-Kpn-372	$\boldsymbol{bla}_{\textbf{NDM-1}}$, $bla_{\text{CTX-M-15}}$, $bla_{\text{OXA-1}}$, $bla_{\text{SHV-182}}$	*aac(6')-Ib, aac(3)-IIa, oqxA1, oqxB1, fosA6, catB3, dfrA14*
NDM-Kpn-373	$\boldsymbol{bla}_{\textbf{NDM-1}}$, $\boldsymbol{bla}_{\textbf{KPC-3}}$, $bla_{\text{OXA-10}}$, $bla_{\text{TEM-1B}}$, $bla_{\text{SHV-89}}$	*aadA1, aadA16, aac(6')-Ib-cr, oqxA, oqxB, fosA, mph(A), qnrS1, catA1, ARR-3, sul1, dfrA27, dfrA12*

NDM-Kpn-374	***bla***$_{\mathbf{NDM\text{-}1}}$**,** ***bla***$_{\mathbf{OXA\text{-}48}}$, *bla*$_{\text{CTX-M-15}}$, *bla*$_{\text{SHV-11}}$	*aph(3')-VI, aadA2, armA, oqxA1, oqxB1, fosA5, mrs(E), mph(A), mph(E), sul1, tet(A), dfrA12*
NDM-Kpn-375	***bla***$_{\mathbf{NDM\text{-}1}}$**,** ***bla***$_{\mathbf{OXA\text{-}48}}$, *bla*$_{\text{CTX-M-15}}$, *bla*$_{\text{SHV-67}}$, *bla*$_{\text{SHV-11}}$	*aadA2, aph(3')-VI, armA, oqxA1, oqxB1, fosA5, mrs(E), mph(A), mph(E), sul1, tet(A), dfrA12*
NDM-Kpn-376	***bla***$_{\mathbf{NDM\text{-}1}}$, *bla*$_{\text{CTX-M-15}}$, *bla*$_{\text{OXA-1}}$, *bla*$_{\text{OXA-9}}$, *bla*$_{\text{TEM-1A}}$, *bla*$_{\text{SHV-11}}$	*aph(3')-VI, aac(6')-Ib, ant(3'')-Ia, armA, aac(6')-Ib-cr, qacE, oqxA1, oqxB1, fosA5, mrs(E), mph(A), mph(E), catB3, qnrS1, ARR-3, sul1, sul2, dfrA5*
NDM-Kpn-377	***bla***$_{\mathbf{NDM\text{-}5}}$, *bla*$_{\text{CTX-M-15}}$, *bla*$_{\text{OXA-1}}$, *bla*$_{\text{OXA-9}}$, *bla*$_{\text{TEM-1A}}$, *bla*$_{\text{SHV-11}}$, *bla*$_{\text{SHV-182}}$	*aph(3')-VI, aadA2, aadA1, aac(6')-Ib3, armA, fosA6, mrs(E), mph(A), mph(E), catA1, catB3, qnrS1, ARR-3, sul1, sul2, dfrA12*
NDM-Kpn-378	***bla***$_{\mathbf{NDM\text{-}1}}$, *bla*$_{\text{CTX-M-15}}$, *bla*$_{\text{OXA-1}}$, *bla*$_{\text{OXA-9}}$, *bla*$_{\text{TEM-1A}}$, *bla*$_{\text{SHV-11}}$, *bla*$_{\text{SHV-67}}$	*aph(3')-VI, aac(6')-Ib, armA, oqxA1, oqxB1, fosA5, mrs(E), mph(A), mph(E), catB3, qnrS1, ARR-3, sul1, sul2, dfrA5*
NDM-Kpn-379	***bla***$_{\mathbf{NDM\text{-}1}}$, *bla*$_{\text{CTX-M-15}}$, *bla*$_{\text{OXA-1}}$, *bla*$_{\text{SHV-182}}$	*aac(6')-Ib, aac(6')-Ib-cr, aac(3)-IIa, oqxA1, oqxB1, fosA3, catB3, dfrA14*
NDM-Kpn-380	***bla***$_{\mathbf{NDM\text{-}5}}$, *bla*$_{\text{SHV-1}}$	*ant(3'')-Ia, qacE, oqxA1, oqxB1, fosA3, sul1, tet(D)*
NDM-Kpn-381	***bla***$_{\mathbf{NDM\text{-}1}}$, *bla*$_{\text{CTX-M-15}}$, *bla*$_{\text{OXA-1}}$, *bla*$_{\text{SHV-182}}$	*aac(6')-Ib, aac(6')-Ib-cr, aac(3)-IIa, oqxA1, oqxB1, fosA3, catB3, dfrA14*
NDM-Kpn-382	***bla***$_{\mathbf{NDM\text{-}1}}$**,** ***bla***$_{\mathbf{OXA\text{-}48}}$, *bla*$_{\text{CTX-M-15}}$, *bla*$_{\text{SHV-11}}$	*aph(3')-VI, aadA2, armA, qacE, oqxA1, oqxB1, fosA5, mrs(E), mph(A), mph(E), sul1, tet(A), dfrA12*
NDM-Kpn-383	***bla***$_{\mathbf{NDM\text{-}1}}$**,** ***bla***$_{\mathbf{OXA\text{-}48}}$, *bla*$_{\text{CTX-M-15}}$, *bla*$_{\text{SHV-67}}$	*aph(3')-VI, aadA2, armA, qacE, oqxA1, oqxB1, fosA5, mrs(E), mph(A), mph(E), sul1, tet(A), dfrA12*
NDM-Kpn-384	***bla***$_{\mathbf{NDM\text{-}1}}$**,** ***bla***$_{\mathbf{OXA\text{-}48}}$, *bla*$_{\text{CTX-M-15}}$, *bla*$_{\text{SHV-11}}$	*aph(3')-VI, aadA2, armA, qacE, oqxA1, oqxB1, fosA5, mrs(E), mph(A), mph(E), sul1, tet(A), dfrA12*
NDM-Kpn-385	***bla***$_{\mathbf{NDM\text{-}1}}$**,** ***bla***$_{\mathbf{OXA\text{-}48}}$, *bla*$_{\text{CTX-M-15}}$, *bla*$_{\text{SHV-11}}$	*aph(3')-VI, aadA2, armA, qacE, oqxA1, oqxB1, fosA5, mrs(E), mph(A), mph(E), sul1, tet(A), dfrA12*
NDM-Kpn-386	***bla***$_{\mathbf{NDM\text{-}1}}$**,** ***bla***$_{\mathbf{OXA\text{-}48}}$, *bla*$_{\text{CTX-M-15}}$, *bla*$_{\text{SHV-11}}$	*aph(3')-VI, aadA2, armA, qacE, oqxA1, oqxB1, fosA5, mrs(E), mph(A), mph(E), sul1, tet(A), dfrA12*
NDM-Kpn-387	***bla***$_{\mathbf{NDM\text{-}1}}$**,** ***bla***$_{\mathbf{OXA\text{-}48}}$, *bla*$_{\text{CTX-M-15}}$, *bla*$_{\text{SHV-11}}$	*aph(3')-VI, aadA2, armA, qacE, oqxA1, oqxB1, fosA5, mrs(E), mph(A), mph(E), sul1, tet(A), dfrA12*
NDM-Kpn-388	***bla***$_{\mathbf{NDM\text{-}1}}$**,** ***bla***$_{\mathbf{OXA\text{-}48}}$, *bla*$_{\text{CTX-M-15}}$, *bla*$_{\text{SHV-11}}$	*aph(3')-VI, aadA2, armA, qacE, oqxA1, oqxB1, fosA5, mrs(E), mph(A), mph(E), sul1, tet(A), dfrA12*
NDM-Kpn-389	***bla***$_{\mathbf{NDM\text{-}1}}$**,** ***bla***$_{\mathbf{OXA\text{-}48}}$, *bla*$_{\text{CTX-M-15}}$, *bla*$_{\text{SHV-11}}$	*aph(3')-VI, aadA2, armA, qacE, oqxA1, oqxB1, fosA5, mrs(E), mph(A), mph(E), sul1, tet(A), dfrA12*
NDM-Kpn-390	***bla***$_{\mathbf{NDM\text{-}1}}$**,** ***bla***$_{\mathbf{OXA\text{-}48}}$, *bla*$_{\text{CTX-M-15}}$, *bla*$_{\text{SHV-11}}$	*aph(3')-VI, aadA2, armA, qacE, oqxA1, oqxB1, fosA5, mrs(E), mph(A), mph(E), sul1, tet(A), dfrA12*
NDM-Kpn-391	***bla***$_{\mathbf{NDM\text{-}1}}$**,** ***bla***$_{\mathbf{OXA\text{-}48}}$, *bla*$_{\text{CTX-M-15}}$, *bla*$_{\text{SHV-11}}$	*aph(3')-VI, aadA2, armA, qacE, oqxA1, oqxB1, fosA5, mrs(E), mph(A), mph(E), sul1, tet(A), dfrA12*
NDM-Kpn-392	***bla***$_{\mathbf{NDM\text{-}1}}$**,** ***bla***$_{\mathbf{OXA\text{-}48}}$, *bla*$_{\text{CTX-M-15}}$, *bla*$_{\text{SHV-11}}$, *bla*$_{\text{SHV-67}}$	*aph(3')-VI, aadA2, armA, oqxA, oqxB, fosA, mrs(E), mph(A), mph(E), sul1, tet(A), dfrA12*
NDM-Kpn-393	***bla***$_{\mathbf{NDM\text{-}1}}$**,** ***bla***$_{\mathbf{OXA\text{-}48}}$, *bla*$_{\text{CTX-M-15}}$, *bla*$_{\text{SHV-11}}$, *bla*$_{\text{SHV-67}}$	*aph(3')-VI, aadA2, armA, oqxA, oqxB, fosA, mrs(E), mph(A), mph(E), sul1, tet(A), dfrA12*
NDM-Kpn-394	***bla***$_{\mathbf{NDM\text{-}1}}$**,** ***bla***$_{\mathbf{OXA\text{-}48}}$, *bla*$_{\text{CTX-M-15}}$, *bla*$_{\text{DHA-1}}$, *bla*$_{\text{SHV-11}}$, *bla*$_{\text{SHV-67}}$	*aac(3)-IId, aph(3')-VI, aadA2, armA, oqxA, oqxB, fosA, mrs(E), mph(A), mph(E), qnrB4, sul1, tet(A), dfrA12*
NDM-Kpn-395	***bla***$_{\mathbf{NDM\text{-}1}}$**,** ***bla***$_{\mathbf{OXA\text{-}48}}$, *bla*$_{\text{CTX-M-15}}$, *bla*$_{\text{SHV-11}}$, *bla*$_{\text{SHV-67}}$	*aph(3')-VI, aadA2, armA, oqxA, oqxB, fosA, mrs(E), mph(A), mph(E), sul1, tet(A), dfrA12*
NDM-Kpn-396	***bla***$_{\mathbf{NDM\text{-}1}}$**,** ***bla***$_{\mathbf{OXA\text{-}48}}$, *bla*$_{\text{CTX-M-15}}$, *bla*$_{\text{SHV-11}}$, *bla*$_{\text{SHV-67}}$	*aph(3')-VI, aadA2, armA, oqxA, oqxB, fosA, mrs(E), mph(A), mph(E), sul1, tet(A), dfrA12*
NDM-Kpn-397	***bla***$_{\mathbf{NDM\text{-}5}}$**,** ***bla***$_{\mathbf{OXA\text{-}48}}$, *bla*$_{\text{CTX-M-15}}$, *bla*$_{\text{OXA-1}}$, *bla*$_{\text{SHV-11}}$, *bla*$_{\text{SHV-67}}$	*aph(3')-VI, aph(3')-Ia, armA, aac(6')-Ib-cr, oqxA, oqxB, fosA, mrs(E), mph(A), mph(E), catB3, qnrS1, ARR-3, sul1, sul2, dfrA5*
NDM-Kpn-398	***bla***$_{\mathbf{NDM\text{-}1}}$**,** ***bla***$_{\mathbf{OXA\text{-}48}}$, *bla*$_{\text{CTX-M-15}}$, *bla*$_{\text{SHV-11}}$, *bla*$_{\text{SHV-67}}$	*aph(3')-VI, aadA2, armA, oqxA, oqxB, fosA, mrs(E), mph(A), mph(E), sul1, tet(A), dfrA12*

NDM-Kpn-399	**_bla_**$_{\textbf{NDM-1}}$, **_bla_**$_{\textbf{OXA-48}}$, _bla_$_{\text{CTX-M-15}}$, _bla_$_{\text{SHV-11}}$, _bla_$_{\text{SHV-67}}$	_aph(3')-VI, aadA2, armA, oqxA, oqxB, fosA, mrs(E), mph(A), mph(E), sul1, tet(A), dfrA12_
NDM-Kpn-400	**_bla_**$_{\textbf{NDM-1}}$, **_bla_**$_{\textbf{OXA-48}}$, _bla_$_{\text{CTX-M-15}}$, _bla_$_{\text{SHV-11}}$, _bla_$_{\text{SHV-67}}$	_aph(3')-VI, aadA2, armA, oqxA, oqxB, fosA, mrs(E), mph(A), mph(E), sul1, tet(A), dfrA12_
NDM-Kpn-401	**_bla_**$_{\textbf{NDM-1}}$, **_bla_**$_{\textbf{OXA-48}}$, _bla_$_{\text{CTX-M-15}}$, _bla_$_{\text{SHV-11}}$, _bla_$_{\text{SHV-67}}$	_aph(3')-VI, aadA2, armA, oqxA, oqxB, fosA, mrs(E), mph(A), mph(E), sul1, tet(A), dfrA12_
NDM-Kpn-402	**_bla_**$_{\textbf{NDM-1}}$, **_bla_**$_{\textbf{OXA-48}}$, _bla_$_{\text{CTX-M-15}}$, _bla_$_{\text{SHV-11}}$, _bla_$_{\text{SHV-67}}$	_aph(3')-VI, aadA2, armA, oqxA, oqxB, fosA, mrs(E), mph(A), mph(E), sul1, tet(A), dfrA12_
NDM-Kpn-403	**_bla_**$_{\textbf{NDM-1}}$, **_bla_**$_{\textbf{OXA-48}}$, _bla_$_{\text{CTX-M-15}}$, _bla_$_{\text{SHV-11}}$, _bla_$_{\text{SHV-67}}$	_aph(3')-VI, aadA2, armA, oqxA, oqxB, fosA, mrs(E), mph(A), mph(E), sul1, tet(A), dfrA12_
NDM-Kpn-404	**_bla_**$_{\textbf{NDM-1}}$, **_bla_**$_{\textbf{OXA-48}}$, _bla_$_{\text{CTX-M-15}}$, _bla_$_{\text{SHV-11}}$, _bla_$_{\text{SHV-67}}$	_aph(3')-VI, aadA2, armA, oqxA, oqxB, fosA, mrs(E), mph(A), mph(E), sul1, tet(A), dfrA12_
NDM-Kpn-405	**_bla_**$_{\textbf{NDM-5}}$, **_bla_**$_{\textbf{OXA-48}}$, _bla_$_{\text{CTX-M-14b}}$, _bla_$_{\text{CTX-M-15}}$, _bla_$_{\text{OXA-9}}$, _bla_$_{\text{TEM-1C}}$, _bla_$_{\text{TEM-1B}}$, _bla_$_{\text{SHV-11}}$, _bla_$_{\text{SHV-67}}$	_aph(6)-Id, aph(3')-VI, aph(3'')-Ib, rmtF, aac(6')-Ib-Hangzhou, aadA1, aph(3')-Ia, oqxA, oqxB, fosA, mph(A), catA1, qnrS1, qnrB1, ARR-2, sul1, dfrA5_
NDM-Kpn-406	**_bla_**$_{\textbf{NDM-1}}$, **_bla_**$_{\textbf{OXA-48}}$, _bla_$_{\text{CTX-M-15}}$, _bla_$_{\text{CTX-M-194}}$, _bla_$_{\text{CTX-M-186}}$, _bla_$_{\text{CTX-M-163}}$, _bla_$_{\text{SHV-11}}$, _bla_$_{\text{SHV-67}}$	_aph(3')-VI, aadA2, armA, oqxA, oqxB, fosA, mrs(E), mph(A), mph(E), sul1, tet(A), dfrA12_
NDM-Kpn-407	**_bla_**$_{\textbf{NDM-1}}$, **_bla_**$_{\textbf{OXA-48}}$, _bla_$_{\text{CTX-M-15}}$, _bla_$_{\text{SHV-11}}$, _bla_$_{\text{SHV-67}}$	_aph(3')-VI, aadA2, armA, oqxA, oqxB, fosA, mrs(E), mph(A), mph(E), sul1, tet(A), dfrA12_
NDM-Kpn-408	**_bla_**$_{\textbf{NDM-1}}$, **_bla_**$_{\textbf{OXA-48}}$, _bla_$_{\text{CTX-M-15}}$, _bla_$_{\text{SHV-11}}$, _bla_$_{\text{SHV-67}}$	_aph(3')-VI, aadA2, armA, oqxA, oqxB, fosA, mrs(E), mph(A), mph(E), sul1, tet(A), dfrA12_
NDM-Kpn-409	**_bla_**$_{\textbf{NDM-1}}$, **_bla_**$_{\textbf{OXA-48}}$, _bla_$_{\text{CTX-M-15}}$, _bla_$_{\text{SHV-11}}$, _bla_$_{\text{SHV-67}}$	_aph(3')-VI, aadA2, armA, oqxA, fosA, mrs(E), mph(A), mph(E), sul1, tet(A), dfrA12_
NDM-Kpn-410	**_bla_**$_{\textbf{NDM-1}}$, **_bla_**$_{\textbf{OXA-48}}$, _bla_$_{\text{CTX-M-15}}$, _bla_$_{\text{SHV-11}}$, _bla_$_{\text{SHV-67}}$	_aph(3')-VI, aadA2, armA, oqxA, oqxB, fosA, mrs(E), mph(A), mph(E), sul1, tet(A), dfrA12_
NDM-Kpn-411	**_bla_**$_{\textbf{NDM-1}}$, **_bla_**$_{\textbf{OXA-48}}$, _bla_$_{\text{CTX-M-15}}$, _bla_$_{\text{SHV-11}}$, _bla_$_{\text{SHV-67}}$	_aph(3')-VI, aadA2, armA, oqxA, oqxB, fosA, mrs(E), mph(A), mph(E), sul1, tet(A), dfrA12_
NDM-Kpn-412	**_bla_**$_{\textbf{NDM-1}}$, **_bla_**$_{\textbf{OXA-48}}$, _bla_$_{\text{CTX-M-15}}$, _bla_$_{\text{SHV-11}}$, _bla_$_{\text{SHV-67}}$	_aph(3')-VI, aadA2, armA, oqxA, oqxB, fosA, mrs(E), mph(A), mph(E), sul1, tet(A), dfrA12_
NDM-Kpn-413	**_bla_**$_{\textbf{NDM-5}}$, **_bla_**$_{\textbf{OXA-48}}$, _bla_$_{\text{CTX-M-15}}$, _bla_$_{\text{OXA-1}}$, _bla_$_{\text{SHV-11}}$, _bla_$_{\text{SHV-67}}$	_aph(3')-Ia, armA, aac(6')-Ib-cr, oqxA, oqxB, fosA, mrs(E), mph(A), mph(E), catB3, qnrS1, ARR-3, sul1, sul2, dfrA5_
NDM-Kpn-414	**_bla_**$_{\textbf{NDM-1}}$, **_bla_**$_{\textbf{OXA-48}}$, _bla_$_{\text{CTX-M-15}}$, _bla_$_{\text{SHV-11}}$, _bla_$_{\text{SHV-67}}$	_aph(3')-VI, aadA2, armA, oqxA, oqxB, fosA, mrs(E), mph(A), mph(E), sul1, tet(A), dfrA12_
NDM-Kpn-415	**_bla_**$_{\textbf{NDM-1}}$, **_bla_**$_{\textbf{OXA-48}}$, _bla_$_{\text{CTX-M-15}}$, _bla_$_{\text{SHV-11}}$, _bla_$_{\text{SHV-67}}$	_aph(3')-VI, aadA2, armA, oqxA, fosA, mrs(E), mph(A), mph(E), sul1, tet(A), dfrA12_
NDM-Kpn-416	**_bla_**$_{\textbf{NDM-1}}$, **_bla_**$_{\textbf{OXA-48}}$, _bla_$_{\text{CTX-M-15}}$, _bla_$_{\text{SHV-11}}$, _bla_$_{\text{SHV-67}}$	_aph(3')-VI, aadA2, armA, oqxA, oqxB, fosA, mrs(E), mph(A), mph(E), sul1, tet(A), dfrA12_
NDM-Kpn-417	**_bla_**$_{\textbf{NDM-1}}$, **_bla_**$_{\textbf{OXA-48}}$, _bla_$_{\text{CTX-M-15}}$, _bla_$_{\text{SHV-11}}$, _bla_$_{\text{SHV-67}}$	_aph(3')-VI, aadA2, armA, oqxA, oqxB, fosA, mrs(E), mph(A), mph(E), sul1, tet(A), dfrA12_
NDM-Kpn-418	**_bla_**$_{\textbf{NDM-1}}$, **_bla_**$_{\textbf{OXA-48}}$, _bla_$_{\text{CTX-M-15}}$, _bla_$_{\text{SHV-11}}$	_aph(3')-VI, aadA2, armA, oqxA, oqxB, fosA, mrs(E), mph(A), mph(E), sul1, tet(A), dfrA12_
NDM-Kpn-419	**_bla_**$_{\textbf{NDM-1}}$, **_bla_**$_{\textbf{OXA-48}}$, _bla_$_{\text{CTX-M-15}}$, _bla_$_{\text{SHV-11}}$, _bla_$_{\text{SHV-67}}$	_aph(3')-VI, aadA2, armA, oqxA, oqxB, fosA, mrs(E), mph(A), mph(E), sul1, tet(A), dfrA12_
NDM-Kpn-420	**_bla_**$_{\textbf{NDM-1}}$, **_bla_**$_{\textbf{OXA-48}}$, _bla_$_{\text{CTX-M-15}}$, _bla_$_{\text{SHV-11}}$, _bla_$_{\text{SHV-67}}$	_aph(3')-VI, aadA2, armA, oqxA, oqxB, fosA, mrs(E), mph(A), mph(E), sul1, tet(A), dfrA12_
NDM-Kpn-421	**_bla_**$_{\textbf{NDM-1}}$, **_bla_**$_{\textbf{OXA-48}}$, _bla_$_{\text{CTX-M-15}}$, _bla_$_{\text{SHV-11}}$, _bla_$_{\text{SHV-67}}$	_aph(3')-VI, aadA2, armA, oqxA, oqxB, fosA, mrs(E), mph(A), mph(E), sul1, tet(A), dfrA12_
NDM-Kpn-422	**_bla_**$_{\textbf{NDM-5}}$, **_bla_**$_{\textbf{OXA-48}}$, _bla_$_{\text{CTX-M-15}}$, _bla_$_{\text{OXA-1}}$, _bla_$_{\text{SHV-11}}$, _bla_$_{\text{SHV-67}}$	_aph(3')-VI, aac(6')-Ib-cr, aph(3')-Ia, armA, oqxA, oqxB, fosA, mrs(E), mph(A), mph(E), catB3, qnrS1, ARR-3, sul1, sul2, dfrA5_
NDM-Kpn-423	**_bla_**$_{\textbf{NDM-1}}$, **_bla_**$_{\textbf{OXA-48}}$, _bla_$_{\text{CTX-M-15}}$, _bla_$_{\text{SHV-11}}$, _bla_$_{\text{SHV-67}}$	_aph(3')-VI, aadA2, armA, oqxA, oqxB, fosA, mrs(E), mph(A), mph(E), sul1, tet(A), dfrA12_

NDM-Kpn-424	**$bla_{\text{NDM-1}}$**, **$bla_{\text{OXA-48}}$**, $bla_{\text{CTX-M-15}}$, $bla_{\text{SHV-11}}$, $bla_{\text{SHV-67}}$	*aph(3')-VI, aadA2, armA, oqxA, oqxB, fosA, mrs(E), mph(A), mph(E), sul1, tet(A), dfrA12*
NDM-Kpn-425	**$bla_{\text{NDM-1}}$**, **$bla_{\text{OXA-48}}$**, $bla_{\text{CTX-M-15}}$, $bla_{\text{SHV-11}}$, $bla_{\text{SHV-67}}$	*aph(3')-VI, aadA2, armA, oqxA, oqxB, fosA, mrs(E), mph(A), mph(E), sul1, tet(A), dfrA12*
NDM-Kpn-426	**$bla_{\text{NDM-5}}$**, **$bla_{\text{OXA-48}}$**, $bla_{\text{CTX-M-14b}}$, $bla_{\text{CTX-M-15}}$, $bla_{\text{OXA-9}}$, $bla_{\text{TEM-1C}}$, $bla_{\text{TEM-1B}}$, $bla_{\text{SHV-199}}$, $bla_{\text{SHV-194}}$, $bla_{\text{SHV-179}}$, $bla_{\text{SHV-145}}$, $bla_{\text{SHV-98}}$, $bla_{\text{SHV-78}}$, $bla_{\text{SHV-26}}$	*aph(6)-Id, aph(3')-VI, aph(3'')-Ib, aac(6')-Ib, aadA1, aph(3')-Ia, aph(3')-VIb, oqxA, oqxB, fosA, mrs(E), mph(A), mph(E), catA1, qnrS1, qnrB1, sul1, sul2, tet(A), dfrA5*
NDM-Kpn-427	**$bla_{\text{NDM-1}}$**, **$bla_{\text{OXA-48}}$**, $bla_{\text{CTX-M-15}}$, $bla_{\text{SHV-11}}$, $bla_{\text{SHV-67}}$	*aph(3')-VI, aadA2, armA, oqxA, oqxB, fosA, mrs(E), mph(A), mph(E), sul1, tet(A), dfrA12*
NDM-Kpn-428	**$bla_{\text{NDM-1}}$**, **$bla_{\text{OXA-48}}$**, $bla_{\text{CTX-M-15}}$, $bla_{\text{SHV-11}}$, $bla_{\text{SHV-67}}$	*aph(3')-VI, aadA2, armA, oqxA, oqxB, fosA, mrs(E), mph(A), mph(E), sul1, tet(A), dfrA12*
NDM-Kpn-429	**$bla_{\text{NDM-1}}$**, **$bla_{\text{OXA-48}}$**, $bla_{\text{CTX-M-15}}$, $bla_{\text{SHV-11}}$, $bla_{\text{SHV-67}}$	*aph(3')-VI, aadA2, armA, oqxA, oqxB, fosA, mrs(E), mph(A), mph(E), sul1, tet(A), dfrA12*
NDM-Kpn-430	**$bla_{\text{NDM-1}}$**, **$bla_{\text{OXA-48}}$**, $bla_{\text{CTX-M-15}}$, $bla_{\text{SHV-11}}$, $bla_{\text{SHV-67}}$	*aph(3')-VI, aadA2, armA, oqxA, oqxB, fosA, mrs(E), mph(A), mph(E), sul1, tet(A), dfrA12*
NDM-Kpn-431	**$bla_{\text{NDM-1}}$**, **$bla_{\text{OXA-48}}$**, $bla_{\text{CTX-M-15}}$, $bla_{\text{SHV-11}}$, $bla_{\text{SHV-67}}$	*aph(3')-VI, aadA2, armA, oqxA, oqxB, fosA, mrs(E), mph(A), mph(E), sul1, tet(A), dfrA12*
NDM-Kpn-432	**$bla_{\text{NDM-1}}$**, **$bla_{\text{OXA-48}}$**, $bla_{\text{CTX-M-15}}$, $bla_{\text{SHV-11}}$, $bla_{\text{SHV-67}}$	*aph(3')-VI, aadA2, armA, oqxA, oqxB, fosA, mrs(E), mph(A), mph(E), sul1, tet(A), dfrA12*
NDM-Kpn-433	**$bla_{\text{NDM-1}}$**, **$bla_{\text{OXA-48}}$**, $bla_{\text{CTX-M-15}}$, $bla_{\text{SHV-11}}$, $bla_{\text{SHV-67}}$	*aph(3')-VI, aadA2, armA, oqxA, oqxB, fosA, mrs(E), mph(A), mph(E), sul1, tet(A), dfrA12*
NDM-Kpn-434	**$bla_{\text{NDM-1}}$**, **$bla_{\text{OXA-48}}$**, $bla_{\text{CTX-M-15}}$, $bla_{\text{SHV-11}}$, $bla_{\text{SHV-67}}$	*aph(3')-VI, aadA2, armA, oqxA, oqxB, fosA, mrs(E), mph(A), mph(E), sul1, tet(A), dfrA12*
NDM-Kpn-435	**$bla_{\text{NDM-1}}$**, **$bla_{\text{OXA-48}}$**, $bla_{\text{CTX-M-15}}$, $bla_{\text{SHV-11}}$, $bla_{\text{SHV-67}}$	*aph(3')-VI, aadA2, armA, oqxA, oqxB, fosA, mrs(E), mph(A), mph(E), sul1, tet(A), dfrA12*
NDM-Kpn-436	**$bla_{\text{NDM-5}}$**, **$bla_{\text{OXA-48}}$**, $bla_{\text{CTX-M-15}}$, $bla_{\text{OXA-1}}$, $bla_{\text{SHV-11}}$	*aph(3')-VI, aph(3')-Ia, armA, aac(6')-Ib-cr, oqxA, oqxB, fosA, mrs(E), mph(A), mph(E), catB3, qnrS1, ARR-3, sul1, sul2, dfrA5*
NDM-Kpn-437	**$bla_{\text{NDM-5}}$**, **$bla_{\text{OXA-48}}$**, $bla_{\text{CTX-M-15}}$, $bla_{\text{OXA-1}}$, $bla_{\text{SHV-11}}$, $bla_{\text{SHV-67}}$	*aph(3')-VI, aph(3')-Ia, armA, aac(6')-Ib-cr, oqxA, oqxB, fosA, mrs(E), mph(A), mph(E), catB3, qnrS1, ARR-3, sul1, sul2, dfrA5*
