# Supplementary material for: Emergence and Dissemination of NDM+OXA-48-like Co-Producing Klebsiella pneumoniae in a Regional Healthcare Network: Seven-Year Surveillance from Latium, Italy
Source: Antibiotics (Basel). 2026 Aug 10;15(8):766. doi: 10.3390/antibiotics15080766 (PMC13509432; doi:10.3390/antibiotics15080766)
Supplement: Supplementary file 1 [file antibiotics-15-00766-s001.zip › Table S4-edited.pdf]

Table S4. Molecular typing and characterization of virulence genes of NDM-Kpn isolates.

Strain	Hospital[1]	Carbapenemase	Typing				Virulence factors[5]	
			ST[2]	CT[3]	*wzi allele*	KL[4]	Virulence genes	Score
NDM-Kpn-1	H-7	**NDM**	11	21	*wzi24*	KL24	*ybt15, ICEKp11*	1
NDM-Kpn-2	H-3	**NDM**	11	21	*wzi24*	KL24	*ybt15, ICEKp11*	1
NDM-Kpn-3	H-13	**NDM + OXA-48-like**	15	17	*wzi93*	KL112	*ybt16, ICEKp12, iuc1, rmp1*	4
NDM-Kpn-4	H-13	**NDM**	147	3	*wzi64*	KL64	*ybt9, ICEKp3, iuc1, rmp1*	4
NDM-Kpn-5	H-13	**NDM**	383	20	-	KL30	*iuc1, rmp1*	3
NDM-Kpn-6	H-13	**NDM + OXA-48-like**	15	17	*wzi93*	KL112	*ybt16, ICEKp12, iuc1, rmp*	4
NDM-Kpn-7	H-5	**NDM**	307	-	*wzi173*	KL102	-	0
NDM-Kpn-8	H-13	**NDM + OXA-48-like**	15	17	*wzi93*	KL112	*ybt16, ICEKp12, iuc1, rmp*	4
NDM-Kpn-9	H-13	**NDM + OXA-48-like**	4853	-	-	KL30	-	0
NDM-Kpn-10	H-13	**NDM + OXA-48-like**	383	20	-	KL30	*iuc1, rmp1*	3
NDM-Kpn-11	H-13	**NDM**	17	-	*wzi19*	KL19	-	0
NDM-Kpn-12	H-18	**NDM**	147	2	*wzi64*	KL64	*ybt9, ICEKp3, iuc1, rmp1*	4
NDM-Kpn-13	H-15	**NDM + OXA-48-like**	383	16	-	KL30	-	0
NDM-Kpn-14	H-5	**NDM**	147	8	*wzi64*	KL64	-	0
NDM-Kpn-15	H-15	**NDM + OXA-48-like**	383	16	-	KL30	-	0
NDM-Kpn-16	H-5	**NDM**	147	8	*wzi64*	KL64	-	0
NDM-Kpn-17	H-9	**NDM**	29	-	*wzi115*	KL54	*ybt10, ICEKp4*	1
NDM-Kpn-18	H-5	**NDM + OXA-48-like**	383	16	-	KL30	-	0
NDM-Kpn-19	H-2	**NDM**	395	15	*wzi2*	KL2	*ybt16, ICEKp12, iuc1, rmp1*	4
NDM-Kpn-20	H-2	**NDM**	395	15	*wzi2*	KL2	*ybt16, ICEKp12, iuc1, rmp1*	4
NDM-Kpn-21	H-5	**NDM**	147	2	*wzi64*	KL64	*ybt9, ICEKp3, iuc1, rmp1*	4
NDM-Kpn-22	H-9	**NDM**	11	10	*wzi24*	KL24	*ybt15, ICEKp11*	1

NDM-Kpn-23	H-7	**NDM**	147	5	*wzi420*	KL10	-	0
NDM-Kpn-24	H-5	**NDM**	395	15	*wzi2*	KL2	*ybt16, ICEKp12, iuc1, rmp1*	4
NDM-Kpn-25	H-7	**NDM**	147	5	*wzi420*	KL10	-	0
NDM-Kpn-26	H-7	**NDM**	147	5	*wzi420*	KL10	-	0
NDM-Kpn-27	H-7	**NDM**	147	5	*wzi420*	KL10	-	0
NDM-Kpn-28	H-3	**NDM**	11	10	*wzi24*	KL24	*ybt15, ICEKp11*	1
NDM-Kpn-29	H-7	**NDM**	147	5	*wzi420*	KL10	-	0
NDM-Kpn-30	H-2	**NDM**	395	15	*wzi2*	KL2	*ybt16, ICEKp12, iuc1, rmp1*	4
NDM-Kpn-31	H-3	**NDM**	147	2	*wzi64*	KL64	*ybt9, ICEKp3, iuc1, rmp1*	4
NDM-Kpn-32	H-3	**NDM**	147	-	*wzi64*	KL64	*ybt9, ICEKp3, iuc1, rmp1*	4
NDM-Kpn-33	H-3	**NDM + KPC**	512	12	*wzi154*	KL107	-	0
NDM-Kpn-34	H-19	**NDM + KPC**	512	12	*wzi154*	KL107	*ybt9, ICEKp3*	1
NDM-Kpn-35	H-3	**NDM**	395	18	*wzi2*	KL2	*ybt16, ICEKp12, iuc1*	4
NDM-Kpn-36	H-23	**NDM**	11	10	*wzi24*	KL24	*ybt15, ICEKp11*	1
NDM-Kpn-37	H-3	**NDM**	11	10	*wzi24*	KL24	*ybt15, ICEKp11*	1
NDM-Kpn-38	H-19	**NDM + KPC**	512	12	*wzi154*	KL107	*ybt9, ICEKp3*	1
NDM-Kpn-39	H-2	**NDM**	11	-	*wzi50*	KL15	*ybt9, ICEKp3*	1
NDM-Kpn-40	H-2	**NDM**	23	-	*wzi77*	KL57	*ybt9, ICEKp3, iuc1, rmp1*	4
NDM-Kpn-41	H-2	**NDM**	147	2	*wzi64*	KL64	*ybt9, ICEKp3, iuc1, rmp1*	4
NDM-Kpn-42	H-3	**NDM**	11	10	*wzi24*	KL24	*ybt15, ICEKp11*	1
NDM-Kpn-43	H-20	**NDM**	11	10	*wzi24*	KL24	*ybt15, ICEKp11*	1
NDM-Kpn-44	H-19	**NDM + KPC**	512	12	*wzi154*	KL107	-	0
NDM-Kpn-45	H-2	**NDM**	147	6	*wzi64*	KL64	*ybt9, ICEKp3*	1
NDM-Kpn-46	H-2	**NDM**	147	6	*wzi64*	KL64	*ybt9, ICEKp3*	1
NDM-Kpn-47	H-2	**NDM**	11	19	*wzi75*	KL105	*ybt9, ICEKp3*	1
NDM-Kpn-48	H-2	**NDM**	147	6	*wzi64*	KL64	*ybt9, ICEKp3*	1

NDM-Kpn-49	H-7	**NDM**	147	3	*wzi64*	KL64	*ybt9, ICEKp3*	1
NDM-Kpn-50	H-2	**NDM**	11	19	*wzi75*	KL105	*ybt9, ICEKp3*	1
NDM-Kpn-51	H-2	**NDM**	147	6	*wzi64*	KL64	*ybt9, ICEKp3*	1
NDM-Kpn-52	H-2	**NDM**	11	19	*wzi75*	KL105	*ybt9, ICEKp3*	1
NDM-Kpn-53	H-3	**NDM**	395	18	*wzi2*	KL2	*ybt16, ICEKp12, iuc1*	4
NDM-Kpn-54	H-3	**NDM**	15	22	*wzi93*	KL112	*ybt16, ICEKp12*	1
NDM-Kpn-55	H-13	**NDM**	147	-	*wzi64*	KL64	*ybt9, ICEKp3*	1
NDM-Kpn-56	H-3	**NDM + OXA-48-like**	147	1	*wzi420*	KL10	*ybt10, ICEKp4*	1
NDM-Kpn-57	H-3	**NDM**	395	18	*wzi2*	KL2	*ybt16, ICEKp12, iuc1*	4
NDM-Kpn-58	H-3	**NDM**	15	22	*wzi93*	KL112	*ybt16, ICEKp12*	1
NDM-Kpn-59	H-28	**NDM**	147	2	*wzi64*	KL64	*ybt9, ICEKp3, iuc1, rmp1*	4
NDM-Kpn-60	H-14	**NDM**	147	2	*wzi64*	KL64	*ybt9, ICEKp3, iuc1, rmp1*	4
NDM-Kpn-61	H-18	**NDM**	147	2	*wzi64*	KL64	*ybt9, ICEKp3, iuc1, rmp1*	4
NDM-Kpn-62	H-18	**NDM**	11	10	*wzi24*	KL24	*ybt15, ICEKp11*	1
NDM-Kpn-63	H-3	**NDM**	11	10	*wzi24*	KL24	*ybt15, ICEKp11*	1
NDM-Kpn-64	H-3	**NDM**	1805	11	-	KL48	*iuc1*	3
NDM-Kpn-65	H-3	**NDM + OXA-48-like**	147	1	*wzi420*	KL10	*ybt10, ICEKp4*	1
NDM-Kpn-66	H-17	**NDM**	147	2	*wzi64*	KL64	*ybt9, ICEKp3, iuc1, rmp1*	4
NDM-Kpn-67	H-17	**NDM**	147	2	*wzi64*	KL64	*ybt9, ICEKp3, iuc1, rmp1*	4
NDM-Kpn-68	H-5	**NDM**	147	2	*wzi64*	KL64	*ybt9, ICEKp3, iuc1, rmp1*	4
NDM-Kpn-69	H-17	**NDM**	147	2	*wzi64*	KL64	*ybt9, ICEKp3, iuc1, rmp1*	4
NDM-Kpn-70	H-17	**NDM**	11	10	*wzi24*	KL24	*ybt15, ICEKp11*	1
NDM-Kpn-71	H-27	**NDM**	1805	11	-	KL48	*iuc1*	3
NDM-Kpn-72	H-2	**NDM**	147	-	*wzi420*	KL10	-	0
NDM-Kpn-73	H-3	**NDM**	11	10	*wzi24*	KL24	*ybt15, ICEKp11*	1
NDM-Kpn-74	H-9	**NDM + OXA-48-like**	147	1	*wzi420*	KL10	*ybt10, ICEKp4*	1

NDM-Kpn-75	H-3	**NDM**	147	2	*wzi64*	KL64	*ybt9, ICEKp3*	1
NDM-Kpn-76	H-3	**NDM**	1805	11	-	KL48	*iuc1*	3
NDM-Kpn-77	H-3	**NDM + OXA-48-like**	147	1	*wzi420*	KL10	*ybt10, ICEKp4*	1
NDM-Kpn-78	H-3	**NDM**	234	-	*wzi272*	KL30	-	0
NDM-Kpn-79	H-3	**NDM**	1805	11	-	KL48	*iuc1*	3
NDM-Kpn-80	H-3	**NDM**	1117	-	*wzi356*	KL117	-	0
NDM-Kpn-81	H-8	**NDM**	147	2	*wzi64*	KL64	*ybt9, ICEKp3, iuc1, rmp1*	4
NDM-Kpn-82	H-5	**NDM + OXA-48-like**	147	1	*wzi420*	KL10	*ybt1, ICEKp4*	1
NDM-Kpn-83	H-9	**NDM + OXA-48-like**	147	1	*wzi420*	KL10	*ybt1, ICEKp4*	1
NDM-Kpn-84	H-2	**NDM**	11	10	*wzi24*	KL24	*ybt15, ICEKp11*	1
NDM-Kpn-85	H-9	**NDM + OXA-48-like**	147	1	*wzi420*	KL10	*ybt1, ICEKp4*	1
NDM-Kpn-86	H-7	**NDM**	147	2	*wzi64*	KL64	*ybt9, ICEKp3, iuc1, rmp1*	4
NDM-Kpn-87	H-2	**NDM + VIM**	147	3	*wzi64*	KL64	*ybt9, ICEKp3*	1
NDM-Kpn-88	H-2	**NDM + VIM**	147	3	*wzi64*	KL64	*ybt9, ICEKp3*	1
NDM-Kpn-89	H-3	**NDM**	11	10	*wzi24*	KL24	*ybt15, ICEKp11*	1
NDM-Kpn-90	H-3	**NDM + VIM**	147	3	*wzi64*	KL64	*ybt9, ICEKp3*	1
NDM-Kpn-91	H-21	**NDM + OXA-48-like**	147	1	*wzi420*	KL10	*ybt1, ICEKp4*	1
NDM-Kpn-92	H-8	**NDM**	1805	11	-	KL48	*iuc1*	3
NDM-Kpn-93	H-3	**NDM + VIM**	147	3	*wzi64*	KL64	*ybt9, ICEKp3*	1
NDM-Kpn-94	H-13	**NDM**	147	-	*wzi64*	KL64	*ybt9, ICEKp3, iuc1, rmp1*	4
NDM-Kpn-95	H-8	**NDM**	1805	11	-	KL48	*iuc1*	3
NDM-Kpn-96	H-21	**NDM + OXA-48-like**	147	1	*wzi420*	KL10	*ybt1, ICEKp4*	1
NDM-Kpn-97	H-4	**NDM + OXA-48-like**	3299	-	*wzi2*	KL2	*ybt16, ICEKp12*	1
NDM-Kpn-98	H-2	**NDM**	11	10	*wzi24*	KL24	*ybt15, ICEKp11*	1
NDM-Kpn-99	H-3	**NDM**	11	10	*wzi24*	KL24	*ybt15, ICEKp11*	1
NDM-Kpn-100	H-2	**NDM**	4081	-	-	KL125	-	0

NDM-Kpn-101	H-2	**NDM**	395	-	*wzi160*	KL39	*ybt16, ICEKp12*	1
NDM-Kpn-102	H-2	**NDM + VIM**	147	3	*wzi64*	KL64	*ybt9, ICEKp3*	1
NDM-Kpn-103	H-3	**NDM + OXA-48-like**	147	1	*wzi420*	KL10	*ybt1, ICEKp4*	1
NDM-Kpn-104	H-1	**NDM**	11	10	*wzi24*	KL24	*ybt15, ICEKp11*	1
NDM-Kpn-105	H-1	**NDM**	1805	11	-	KL48	-	0
NDM-Kpn-106	H-1	**NDM**	11	10	*wzi24*	KL24	*ybt15, ICEKp11*	1
NDM-Kpn-107	H-1	**NDM + OXA-48-like**	383	-	-	KL30	*iuc1*	3
NDM-Kpn-108	H-1	**NDM**	11	10	*wzi24*	KL24	*ybt15, ICEKp11*	1
NDM-Kpn-109	H-1	**NDM**	11	10	*wzi24*	KL24	*ybt15, ICEKp11*	1
NDM-Kpn-110	H-1	**NDM**	11	10	*wzi24*	KL24	*ybt15, ICEKp11*	1
NDM-Kpn-111	H-1	**NDM**	147	-	*wzi64*	KL64	*Ybt9, ICEKp3*	1
NDM-Kpn-112	H-1	**NDM**	147	7	*wzi64*	KL64	*ybt9, ICEKp3, iuc1, rmp1*	4
NDM-Kpn-113	H-1	**NDM + OXA-48-like**	147	1	*wzi420*	KL10	*ybt1, ICEKp4*	1
NDM-Kpn-114	H-1	**NDM**	11	10	*wzi24*	KL24	*ybt15, ICEKp11*	1
NDM-Kpn-115	H-1	**NDM**	11	10	*wzi24*	KL24	*ybt15, ICEKp11*	1
NDM-Kpn-116	H-2	**NDM + VIM**	147	3	*wzi64*	KL64	*ybt9, ICEKp3*	1
NDM-Kpn-117	H-7	**NDM**	14	23	*wzi2*	KL2	-	0
NDM-Kpn-118	H-3	**NDM**	11	10	*wzi24*	KL24	*ybt15, ICEKp11*	1
NDM-Kpn-119	H-22	**NDM**	147	3	*wzi64*	KL64	*ybt9, ICEKp3, iuc1, rmp1*	4
NDM-Kpn-120	H-3	**NDM**	147	2	*wzi64*	KL64	*ybt9, ICEKp3, iuc1, rmp1*	4
NDM-Kpn-121	H-7	**NDM + OXA-48-like**	147	1	*wzi420*	KL10	*ybt1, ICEKp4*	1
NDM-Kpn-122	H-9	**NDM**	395	-	*wzi2*	KL2	*ybt16, ICEKp12, iuc1*	4
NDM-Kpn-123	H-2	**NDM + VIM**	147	3	*wzi64*	KL64	*ybt9, ICEKp3*	1
NDM-Kpn-124	H-1	**NDM + OXA-48-like**	6118	-	-	KL30	*iuc1*	3
NDM-Kpn-125	H-1	**NDM**	11	10	*wzi24*	KL24	*ybt15, ICEKp11*	1
NDM-Kpn-126	H-1	**NDM + OXA-48-like**	147	1	*wzi420*	KL10	*ybt1, ICEKp4*	1

NDM-Kpn-127	H-20	**NDM**	1805	11	-	KL48	*iuc1, rmpA2, rmpADC*	3
NDM-Kpn-128	H-2	**NDM**	147	2	*wzi64*	KL64	*ybt9, ICEKp3, iuc1*	4
NDM-Kpn-129	H-1	**NDM + OXA-48-like**	147	1	*wzi420*	KL10	*ybt1, ICEKp4*	1
NDM-Kpn-130	H-1	**NDM + KPC**	11	10	*wzi24*	KL24	*ybt15, ICEKp11*	1
NDM-Kpn-131	H-1	**NDM**	11	10	*wzi24*	KL24	*ybt15, ICEKp11*	1
NDM-Kpn-132	H-2	**NDM**	147	2	*wzi64*	KL64	*ybt9, ICEKp3, iuc1*	4
NDM-Kpn-133	H-3	**NDM + OXA-48-like**	147	1	*wzi420*	KL10	*ybt1, ICEKp4*	1
NDM-Kpn-134	H-9	**NDM + OXA-48-like**	147	1	*wzi420*	KL10	*ybt1, ICEKp4*	1
NDM-Kpn-135	H-2	**NDM + VIM**	147	3	*wzi64*	KL64	*ybt9, ICEKp3*	1
NDM-Kpn-136	H-2	**NDM**	147	7	*wzi64*	KL64	*ybt9, ICEKp3, iuc1*	4
NDM-Kpn-137	H-2	**NDM + VIM**	147	3	*wzi64*	KL64	*ybt9, ICEKp3*	1
NDM-Kpn-138	H-3	**NDM**	11	10	*wzi24*	KL24	*ybt15, ICEKp11*	1
NDM-Kpn-139	H-1	**NDM**	1805	11	-	KL48	*iuc1*	3
NDM-Kpn-140	H-1	**NDM**	11	10	*wzi24*	KL24	*ybt15, ICEKp11*	1
NDM-Kpn-141	H-1	**NDM + OXA-48-like**	147	1	*wzi420*	KL10	*ybt1, ICEKp4*	1
NDM-Kpn-142	H-3	**NDM**	11	10	*wzi24*	KL24	*ybt15, ICEKp11*	1
NDM-Kpn-143	H-15	**NDM + OXA-48-like**	147	1	*wzi420*	KL10	*ybt1, ICEKp4*	1
NDM-Kpn-144	H-5	**NDM + VIM**	147	3	*wzi64*	KL64	*ybt9, ICEKp3*	1
NDM-Kpn-145	H-12	**NDM**	147	2	*wzi64*	KL64	*ybt9, ICEKp3, iuc1*	4
NDM-Kpn-146	H-3	**NDM + OXA-48-like**	147	1	*wzi420*	KL10	*ybt1, ICEKp4*	1
NDM-Kpn-147	H-2	**NDM + VIM**	147	3	*wzi64*	KL64	*ybt9, ICEKp3*	1
NDM-Kpn-148	H-3	**NDM + OXA-48-like**	147	1	*wzi420*	KL10	*ybt1, ICEKp4*	1
NDM-Kpn-149	H-6	**NDM + OXA-48-like**	147	1	*wzi420*	KL10	*ybt1, ICEKp4*	1
NDM-Kpn-150	H-3	**NDM + OXA-48-like**	147	1	*wzi420*	KL10	*ybt1, ICEKp4*	1
NDM-Kpn-151	H-3	**NDM**	11	10	*wzi24*	KL24	*ybt15, ICEKp11*	1
NDM-Kpn-152	H-2	**NDM + VIM**	147	3	*wzi64*	KL64	*ybt9, ICEKp3*	1

NDM-Kpn-153	H-3	**NDM + OXA-48-like**	147	1	*wzi420*	KL10	*ybt1, ICEKp4*	1
NDM-Kpn-154	H-3	**NDM + KPC**	512	12	*wzi154*	KL107	*ybt9, ICEKp3*	1
NDM-Kpn-155	H-3	**NDM + OXA-48-like**	147	1	*wzi420*	KL10	*ybt1, ICEKp4*	1
NDM-Kpn-156	H-15	**NDM + OXA-48-like**	147	1	*wzi420*	KL10	*ybt1, ICEKp4*	1
NDM-Kpn-157	H-15	**NDM + OXA-48-like**	147	1	*wzi420*	KL10	*ybt1, ICEKp4*	1
NDM-Kpn-158	H-10	**NDM + OXA-48-like**	147	1	*wzi420*	KL10	*ybt1, ICEKp4*	1
NDM-Kpn-159	H-10	**NDM + OXA-48-like**	147	1	*wzi420*	KL10	*ybt1, ICEKp4*	1
NDM-Kpn-160	H-10	**NDM + OXA-48-like**	147	1	*wzi420*	KL10	*ybt1, ICEKp4*	1
NDM-Kpn-161	H-26	**NDM**	512	13	*wzi154*	KL107	*ybt8; ICEKp9*	1
NDM-Kpn-162	H-5	**NDM + VIM**	147	3	*wzi64*	KL64	*ybt9, ICEKp3*	1
NDM-Kpn-163	H-3	**NDM + OXA-48-like**	147	1	*wzi420*	KL10	*ybt1, ICEKp4*	1
NDM-Kpn-164	H-2	**NDM + VIM**	147	3	*wzi64*	KL64	*ybt9, ICEKp3*	1
NDM-Kpn-165	H-5	**NDM + OXA-48-like**	147	1	*wzi420*	KL10	*ybt1, ICEKp4*	1
NDM-Kpn-166	H-2	**NDM**	147	2	*wzi64*	KL64	*ybt9; ICEKp?, iuc1, rmp 1*	4
NDM-Kpn-167	H-2	**NDM + VIM**	147	3	*wzi64*	KL64	*ybt9, ICEKp3*	1
NDM-Kpn-168	H-2	**NDM**	11	10	*wzi24*	KL24	*ybt15, ICEKp11*	1
NDM-Kpn-169	H-2	**NDM**	11	10	*wzi24*	KL24	*ybt15, ICEKp11*	1
NDM-Kpn-170	H-2	**NDM**	512	13	*wzi154*	KL107	*ybt8; ICEKp9*	1
NDM-Kpn-171	H-2	**NDM + OXA-48-like**	147	1	*wzi420*	KL10	*ybt1, ICEKp4*	1
NDM-Kpn-172	H-2	**NDM**	11	10	*wzi24*	KL24	*ybt15, ICEKp11*	1
NDM-Kpn-173	H-2	**NDM + VIM**	147	3	*wzi64*	KL64	*ybt9, ICEKp3*	1
NDM-Kpn-174	H-2	**NDM + OXA-48-like**	147	1	*wzi420*	KL10	*ybt1, ICEKp4*	1
NDM-Kpn-175	H-3	**NDM + KPC**	512	12	*wzi154*	KL107	*ybt9, ICEKp3*	1
NDM-Kpn-176	H-3	**NDM + OXA-48-like**	147	1	*wzi420*	KL10	*ybt1, ICEKp4*	1
NDM-Kpn-177	H-3	**NDM + OXA-48-like**	147	1	*wzi420*	KL10	*ybt1, ICEKp4*	1
NDM-Kpn-178	H-3	**NDM + OXA-48-like**	147	1	*wzi420*	KL10	*ybt1, ICEKp4*	1

NDM-Kpn-179	H-11	**NDM + OXA-48-like**	147	1	*wzi420*	KL10	*ybt1, ICEKp4*	1
NDM-Kpn-180	H-14	**NDM + OXA-48-like**	147	1	*wzi420*	KL10	*ybt1, ICEKp4*	1
NDM-Kpn-181	H-2	**NDM + VIM**	147	3	*wzi64*	KL64	*ybt9, ICEKp3*	1
NDM-Kpn-182	H-3	**NDM**	147	2	*wzi64*	KL64	*ybt9, ICEKp3*	1
NDM-Kpn-183	H-1	**NDM**	1805	11	-	KL48	*rmpA2, iuc1*	3
NDM-Kpn-184	H-7	**NDM**	14	23	*wzi2*	KL2	-	0
NDM-Kpn-185	H-9	**NDM**	11	10	*wzi24*	KL24	*ybt15, ICEKp11*	1
NDM-Kpn-186	H-9	**NDM**	11	10	*wzi24*	KL24	*ybt15, ICEKp11*	1
NDM-Kpn-187	H-2	**NDM + VIM**	147	3	*wzi64*	KL64	*ybt9, ICEKp3*	1
NDM-Kpn-188	H-2	**NDM + VIM**	147	3	*wzi64*	KL64	*ybt9, ICEKp3*	1
NDM-Kpn-189	H-2	**NDM + VIM**	147	3	*wzi64*	KL64	*ybt9, ICEKp3*	1
NDM-Kpn-190	H-2	**NDM**	147	-	*wzi104*	KL51	*ybt9; ICEKp?*	1
NDM-Kpn-191	H-3	**NDM + OXA-48-like**	147	1	*wzi420*	KL10	*ybt1, ICEKp4*	1
NDM-Kpn-192	H-6	**NDM + OXA-48-like**	147	1	*wzi420*	KL10	*ybt1, ICEKp4*	1
NDM-Kpn-193	H-8	**NDM**	147	2	*wzi64*	KL64	*rmp1; ybt9; ICEKp3; iuc1*	4
NDM-Kpn-194	H-3	**NDM**	11	10	*wzi24*	KL24	*ybt15, ICEKp11*	1
NDM-Kpn-195	H-14	**NDM**	11	10	*wzi24*	KL24	*ybt15, ICEKp11*	1
NDM-Kpn-196	H-5	**NDM + OXA-48-like**	147	1	*wzi420*	KL10	*ybt1, ICEKp4*	1
NDM-Kpn-197	H-2	**NDM**	11	10	*wzi24*	KL24	*ybt15, ICEKp11*	1
NDM-Kpn-198	H-2	**NDM**	11	10	*wzi24*	KL24	*ybt15, ICEKp11*	1
NDM-Kpn-199	H-2	**NDM**	11	10	*wzi24*	KL24	*ybt15, ICEKp11*	1
NDM-Kpn-200	H-2	**NDM + VIM**	147	3	*wzi64*	KL64	*ybt9, ICEKp3*	1
NDM-Kpn-201	H-16	**NDM + OXA-48-like**	147	1	*wzi420*	KL10	*ybt1, ICEKp4*	1
NDM-Kpn-202	H-9	**NDM**	11	10	*wzi24*	KL24	*ybt15, ICEKp11*	1
NDM-Kpn-203	H-4	**NDM + OXA-48-like**	147	1	*wzi420*	KL10	*ybt1, ICEKp4*	1
NDM-Kpn-204	H-4	**NDM + OXA-48-like**	147	1	*wzi420*	KL10	*ybt1, ICEKp4*	1

NDM-Kpn-205	H-4	**NDM + OXA-48-like**	147	1	*wzi420*	KL10	*ybt1, ICEKp4*	1
NDM-Kpn-206	H-4	**NDM + OXA-48-like**	147	1	*wzi420*	KL10	*ybt1, ICEKp4*	1
NDM-Kpn-207	H-2	**NDM**	11	10	*wzi24*	KL24	*ybt15, ICEKp11*	1
NDM-Kpn-208	H-2	**NDM + VIM**	147	3	*wzi64*	KL64	*ybt9, ICEKp3*	1
NDM-Kpn-209	H-2	**NDM + OXA-48-like**	147	1	*wzi420*	KL10	*ybt1, ICEKp4*	1
NDM-Kpn-210	H-4	**NDM + OXA-48-like**	147	1	*wzi420*	KL10	*ybt1, ICEKp4*	1
NDM-Kpn-211	H-5	**NDM + VIM**	147	3	*wzi64*	KL64	*ybt9, ICEKp3*	1
NDM-Kpn-212	H-11	**NDM + OXA-48-like**	147	1	*wzi420*	KL10	*ybt1, ICEKp4*	1
NDM-Kpn-213	H-11	**NDM + OXA-48-like**	147	1	*wzi420*	KL10	*ybt1, ICEKp4*	1
NDM-Kpn-214	H-11	**NDM + OXA-48-like**	147	1	*wzi420*	KL10	*ybt1, ICEKp4*	1
NDM-Kpn-215	H-11	**NDM + OXA-48-like**	147	1	*wzi420*	KL10	*ybt1, ICEKp4*	1
NDM-Kpn-216	H-2	**NDM**	11	10	*wzi24*	KL24	*ybt15, ICEKp11*	1
NDM-Kpn-217	H-6	**NDM + OXA-48-like**	147	1	*wzi420*	KL10	*ybt1, ICEKp4*	1
NDM-Kpn-218	H-3	**NDM + OXA-48-like**	11	10	*wzi24*	KL24	*ybt15, ICEKp11*	1
NDM-Kpn-219	H-7	**NDM + OXA-48-like**	147	1	*wzi420*	KL10	*ybt1, ICEKp4*	1
NDM-Kpn-220	H-20	**NDM**	147	2	*wzi64*	KL64	*rmp1; ybt9; ICEKp3, iuc1*	4
NDM-Kpn-221	H-7	**NDM + OXA-48-like**	147	9	-	KL64	*rmp1; ybt9; ICEKp?; iuc1*	4
NDM-Kpn-222	H-3	**NDM + KPC**	307	-	*wzi64*	KL64	*ybt9, ICEKp3*	1
NDM-Kpn-223	H-3	**NDM**	147	2	*wzi64*	KL64	*ybt9; ICEKp3*	1
NDM-Kpn-224	H-7	**NDM + OXA-48-like**	147	1	*wzi420*	KL10	*ybt1, ICEKp4*	1
NDM-Kpn-225	H-10	**NDM**	147	1	*wzi420*	KL10	*ybt1, ICEKp4*	1
NDM-Kpn-226	H-10	**NDM**	11	10	*wzi24*	KL24	*ybt15, ICEKp11*	1
NDM-Kpn-227	H-10	**NDM**	11	10	*wzi24*	KL24	*ybt15, ICEKp11*	1
NDM-Kpn-228	H-2	**NDM**	11	10	*wzi24*	KL24	*ybt15, ICEKp11*	1
NDM-Kpn-229	H-5	**NDM + VIM**	147	3	*wzi64*	KL64	*ybt9, ICEKp3*	1
NDM-Kpn-230	H-14	**NDM**	147	2	*wzi64*	KL64	*ybt9, ICEKp3*	1

NDM-Kpn-231	H-6	**NDM + OXA-48-like**	147	1	*wzi420*	KL10	*ybt1, ICEKp4*	1
NDM-Kpn-232	H-6	**NDM + OXA-48-like**	147	1	*wzi420*	KL10	*ybt1, ICEKp4*	1
NDM-Kpn-233	H-2	**NDM + OXA-48-like**	8609	1	*wzi420*	KL10	*ybt1, ICEKp4*	1
NDM-Kpn-234	H-4	**NDM + OXA-48-like**	147	1	*wzi420*	KL10	*ybt1, ICEKp4*	1
NDM-Kpn-235	H-4	**NDM + OXA-48-like**	147	1	*wzi420*	KL10	*ybt1, ICEKp4*	1
NDM-Kpn-236	H-4	**NDM + OXA-48-like**	147	1	*wzi420*	KL10	*ybt1, ICEKp4*	1
NDM-Kpn-237	H-4	**NDM + OXA-48-like**	147	1	*wzi420*	KL10	*ybt1, ICEKp4*	1
NDM-Kpn-238	H-4	**NDM + OXA-48-like**	147	1	*wzi420*	KL10	*ybt1, ICEKp4*	1
NDM-Kpn-239	H-4	**NDM + OXA-48-like**	147	1	*wzi420*	KL10	*ybt1, ICEKp4*	1
NDM-Kpn-240	H-7	**NDM + OXA-48-like**	147	1	*wzi420*	KL10	*ybt1, ICEKp4*	1
NDM-Kpn-241	H-1	**NDM**	11	10	*wzi24*	KL24	*ybt15, ICEKp11*	1
NDM-Kpn-242	H-1	**NDM + OXA-48-like**	147	1	*wzi420*	KL10	*ybt1, ICEKp4*	1
NDM-Kpn-243	H-1	**NDM**	11	10	*wzi24*	KL24	*ybt15, ICEKp11*	1
NDM-Kpn-244	H-1	**NDM + OXA-48-like**	147	1	*wzi420*	KL10	*ybt1, ICEKp4*	1
NDM-Kpn-245	H-1	**NDM**	6668	14	*wzi64*	KL64	*ybt9; ICEKp?*	1
NDM-Kpn-246	H-1	**NDM + KPC**	11	10	*wzi24*	KL24	*ybt15, ICEKp11*	1
NDM-Kpn-247	H-1	**NDM**	512	13	*wzi154*	KL107	*ybt8; ICEKp9*	1
NDM-Kpn-248	H-1	**NDM + OXA-48-like**	147	4	*wzi104*	KL51	*ybt9; ICEKp?*	1
NDM-Kpn-249	H-1	**NDM**	6668	14	*wzi64*	KL64	*ybt9; ICEKp?*	1
NDM-Kpn-250	H-1	**NDM**	11	10	*wzi24*	KL24	*ybt15, ICEKp11*	1
NDM-Kpn-251	H-1	**NDM**	11	10	*wzi24*	KL24	*ybt15, ICEKp11*	1
NDM-Kpn-252	H-1	**NDM**	11	10	*wzi24*	KL24	*ybt15, ICEKp11*	1
NDM-Kpn-253	H-1	**NDM**	11	10	*wzi24*	KL24	*ybt15, ICEKp11*	1
NDM-Kpn-254	H-5	**NDM + OXA-48-like**	147	1	*wzi420*	KL10	*ybt1, ICEKp4*	1
NDM-Kpn-255	H-3	**NDM + OXA-48-like**	147	1	*wzi420*	KL10	*ybt1, ICEKp4*	1
NDM-Kpn-256	H-11	**NDM + OXA-48-like**	147	1	*wzi420*	KL10	*ybt1, ICEKp4*	1

NDM-Kpn-257	H-11	**NDM + OXA-48-like**	147	1	*wzi420*	KL10	*ybt1, ICEKp4*	1
NDM-Kpn-258	H-14	**NDM + OXA-48-like**	147	1	*wzi420*	KL10	*ybt1, ICEKp4*	1
NDM-Kpn-259	H-6	**NDM + OXA-48-like**	147	1	*wzi420*	KL10	*ybt1, ICEKp4*	1
NDM-Kpn-260	H-1	**NDM**	147	2	*wzi64*	KL64	*ybt9, ICEKp3*	1
NDM-Kpn-261	H-1	**NDM**	11	10	*wzi24*	KL24	*ybt15, ICEKp11*	1
NDM-Kpn-262	H-1	**NDM**	11	10	*wzi24*	KL24	*ybt15, ICEKp11*	1
NDM-Kpn-263	H-1	**NDM**	11	10	*wzi24*	KL24	*ybt15, ICEKp11*	1
NDM-Kpn-264	H-1	**NDM**	512	13	*wzi154*	KL107	*ybt8, ICEKp9*	1
NDM-Kpn-265	H-1	**NDM + OXA-48-like**	147	1	*wzi420*	KL10	*ybt1, ICEKp4*	1
NDM-Kpn-266	H-1	**NDM**	11	10	*wzi24*	KL24	-	0
NDM-Kpn-267	H-1	**NDM**	11	10	*wzi24*	KL24	*ybt15, ICEKp11*	1
NDM-Kpn-268	H-1	**NDM**	512	13	*wzi154*	KL107	*ybt8, ICEKp9*	1
NDM-Kpn-269	H-1	**NDM + OXA-48-like**	147	4	*wzi104*	KL51	*ybt9, ICEKp?*	1
NDM-Kpn-270	H-1	**NDM + OXA-48-like**	147	1	*wzi420*	KL10	*ybt1, ICEKp4*	1
NDM-Kpn-271	H-5	**NDM**	147	2	*wzi64*	KL64	*ybt9, ICEKp3, iuc1*	4
NDM-Kpn-272	H-5	**NDM**	147	2	*wzi64*	KL64	*ybt9, ICEKp3, iuc1*	4
NDM-Kpn-273	H-3	**NDM**	11	10	*wzi24*	KL24	*ybt15, ICEKp11*	1
NDM-Kpn-274	H-6	**NDM + OXA-48-like**	147	1	*wzi420*	KL10	*ybt1, ICEKp4*	1
NDM-Kpn-275	H-10	**NDM**	11	10	*wzi24*	KL24	*ybt15, ICEKp11*	1
NDM-Kpn-276	H-10	**NDM**	147	2	*wzi64*	KL64	*ybt9, ICEKp3, iuc1*	4
NDM-Kpn-277	H-10	**NDM**	147	2	*wzi64*	KL64	*ybt9, ICEKp3*	1
NDM-Kpn-278	H-10	**NDM**	147	2	*wzi64*	KL64	*ybt9, ICEKp3*	1
NDM-Kpn-279	H-3	**NDM**	512	13	*wzi154*	KL107	*ybt8; ICEKp9*	1
NDM-Kpn-280	H-14	**NDM + OXA-48-like**	147	1	*wzi420*	KL10	*ybt1, ICEKp4*	1
NDM-Kpn-281	H-14	**NDM**	11	10	*wzi24*	KL24	*ybt15, ICEKp11*	1
NDM-Kpn-282	H-5	**NDM**	1805	11	-	KL48	*iuc1*	3

NDM-Kpn-283	H-12	**NDM**	147	2	*wzi64*	KL64	*ybt9, ICEKp3, iuc1*	4
NDM-Kpn-284	H-12	**NDM**	147	2	*wzi64*	KL64	*ybt9, ICEKp3, iuc1*	4
NDM-Kpn-285	H-12	**NDM**	147	2	*wzi64*	KL64	*ybt9, ICEKp3, iuc1*	4
NDM-Kpn-286	H-12	**NDM**	200	-	*wzi39*	KL13	-	0
NDM-Kpn-287	H-12	**NDM**	147	2	*wzi64*	KL64	*ybt9, ICEKp3, iuc1*	4
NDM-Kpn-288	H-16	**NDM + OXA-48-like**	147	1	*wzi420*	KL10	*ybt1, ICEKp4*	1
NDM-Kpn-289	H-1	**NDM**	11	10	*wzi24*	KL24	*ybt15, ICEKp11*	1
NDM-Kpn-290	H-1	**NDM**	147	7	*wzi64*	KL64	*ybt9, ICEKp?, iuc1*	4
NDM-Kpn-291	H-1	**NDM**	512	13	*wzi154*	KL107	*ybt8, ICEKp9*	1
NDM-Kpn-292	H-1	**NDM**	11	10	*wzi24*	KL24	*ybt15, ICEKp11*	1
NDM-Kpn-293	H-7	**NDM + OXA-48-like**	147	1	*wzi420*	KL10	*ybt1, ICEKp4*	1
NDM-Kpn-294	H-14	**NDM + OXA-48-like**	147	1	*wzi420*	KL10	*ybt1, ICEKp4*	1
NDM-Kpn-295	H-4	**NDM + OXA-48-like**	147	1	*wzi420*	KL10	*ybt1, ICEKp4*	1
NDM-Kpn-296	H-4	**NDM + OXA-48-like**	147	1	*wzi420*	KL10	*ybt1, ICEKp4*	1
NDM-Kpn-297	H-4	**NDM**	16	-	*wzi230*	KL81	*ybt16, ICEKp12*	1
NDM-Kpn-298	H-4	**NDM + OXA-48-like**	147	1	*wzi420*	KL10	*ybt1, ICEKp4*	1
NDM-Kpn-299	H-6	**NDM + OXA-48-like**	147	1	*wzi420*	KL10	*ybt1, ICEKp4*	1
NDM-Kpn-300	H-8	**NDM + OXA-48-like**	147	1	*wzi420*	KL10	*ybt1, ICEKp4*	1
NDM-Kpn-301	H-8	**NDM**	147	3	*wzi64*	KL64	*ybt9, ICEKp3*	1
NDM-Kpn-302	H-8	**NDM**	147	2	*wzi64*	KL64	*ybt9, ICEKp3, iuc1*	4
NDM-Kpn-303	H-8	**NDM + OXA-48-like**	unknown	25	-	KL30	*iuc1*	3
NDM-Kpn-304	H-8	**NDM**	147	2	*wzi64*	KL64	*ybt9, ICEKp3, iuc1*	4
NDM-Kpn-305	H-8	**NDM + OXA-48-like**	147	1	*wzi420*	KL10	*ybt1, ICEKp4*	1
NDM-Kpn-306	H-8	**NDM + OXA-48-like**	unknown	25	-	KL30	*iuc1*	3
NDM-Kpn-307	H-8	**NDM**	147	2	*wzi64*	KL64	*ybt9, ICEKp3, iuc1*	4
NDM-Kpn-308	H-5	**NDM + KPC**	11	-	*wzi209*	KL47	*ybt9, ICEKp3, iuc1*	4

NDM-Kpn-309	H-3	**NDM**	147	2	*wzi64*	KL64	*ybt9, ICEKp3, iuc1*	4
NDM-Kpn-310	H-2	**NDM + OXA-48-like**	147	1	*wzi420*	KL10	*ybt1, ICEKp4*	1
NDM-Kpn-311	H-3	**NDM**	11	10	*wzi24*	KL24	*ybt15, ICEKp11*	1
NDM-Kpn-312	H-3	**NDM**	11	10	*wzi24*	KL24	*ybt15, ICEKp11*	1
NDM-Kpn-313	H-6	**NDM + OXA-48-like**	147	1	*wzi420*	KL10	*ybt1, ICEKp4*	1
NDM-Kpn-314	H-5	**NDM+ VIM**	147	3	*wzi64*	KL64	*ybt9, ICEKp3*	1
NDM-Kpn-315	H-4	**NDM + OXA-48-like**	147	1	*wzi420*	KL10	*ybt1, ICEKp4*	1
NDM-Kpn-316	H-4	**NDM + OXA-48-like**	147	1	*wzi420*	KL10	*ybt1, ICEKp4*	1
NDM-Kpn-317	H-4	**NDM**	147	1	*wzi420*	KL10	*ybt1, ICEKp4*	1
NDM-Kpn-318	H-4	**NDM + OXA-48-like**	147	1	*wzi420*	KL10	*ybt1, ICEKp4*	1
NDM-Kpn-319	H-4	**NDM + OXA-48-like**	147	1	*wzi420*	KL10	*ybt1, ICEKp4*	1
NDM-Kpn-320	H-2	**NDM + OXA-48-like**	147	1	*wzi420*	KL10	*ybt1, ICEKp4*	1
NDM-Kpn-321	H-2	**NDM**	11	10	*wzi24*	KL24	*ybt15, ICEKp11*	1
NDM-Kpn-322	H-6	**NDM + OXA-48-like**	147	1	*wzi420*	KL10	*ybt1, ICEKp4*	1
NDM-Kpn-323	H-1	**NDM**	11	10	*wzi24*	KL24	*ybt15, ICEKp11*	1
NDM-Kpn-324	H-1	**NDM + OXA-48-like**	147	1	*wzi420*	KL10	*ybt1, ICEKp4*	1
NDM-Kpn-325	H-1	**NDM**	11	10	*wzi24*	KL24	*ybt15, ICEKp11*	1
NDM-Kpn-326	H-1	**NDM + OXA-48-like**	147	1	*wzi420*	KL10	*ybt1, ICEKp4*	1
NDM-Kpn-327	H-1	**NDM**	6668	14	*wzi64*	KL64	*ybt9, ICEKp?*	1
NDM-Kpn-328	H-1	**NDM**	11	10	*wzi24*	KL24	*ybt15, ICEKp11*	1
NDM-Kpn-329	H-1	**NDM + OXA-48-like**	147	1	*wzi420*	KL10	*ybt1, ICEKp4*	1
NDM-Kpn-330	H-1	**NDM**	147	1	*wzi420*	KL10	*ybt1, ICEKp4*	1
NDM-Kpn-331	H-1	**NDM + OXA-48-like**	147	1	*wzi420*	KL10	*ybt1, ICEKp4*	1
NDM-Kpn-332	H-1	**NDM + OXA-48-like**	147	4	*wzi104*	KL51	*ybt9, ICEKp?*	1
NDM-Kpn-333	H-10	**NDM**	147	2	*wzi64*	KL64	*ybt9, ICEKp3*	1
NDM-Kpn-334	H-10	**NDM**	6668	14	*wzi64*	KL64	*ybt9, ICEKp?*	1

NDM-Kpn-335	H-5	**NDM + VIM**	147	3	*wzi64*	KL64	*ybt9, ICEKp3*	1
NDM-Kpn-336	H-3	**NDM + OXA-48-like**	147	1	*wzi420*	KL10	*ybt1, ICEKp4*	1
NDM-Kpn-337	H-3	**NDM**	20	24	*wzi84*	KL28	-	0
NDM-Kpn-338	H-3	**NDM**	20	24	*wzi84*	KL28	-	0
NDM-Kpn-339	H-24	**NDM + OXA-48-like**	147	1	*wzi420*	KL10	*ybt1, ICEKp4*	1
NDM-Kpn-340	H-6	**NDM + OXA-48-like**	147	1	*wzi420*	KL10	-	0
NDM-Kpn-341	H-6	**NDM + OXA-48-like**	147	1	*wzi420*	KL10	*ybt1, ICEKp4*	1
NDM-Kpn-342	H-6	**NDM + OXA-48-like**	147	1	*wzi420*	KL10	*ybt1, ICEKp4*	1
NDM-Kpn-343	H-2	**NDM + OXA-48-like**	147	1	*wzi420*	KL10	*ybt1, ICEKp4*	1
NDM-Kpn-344	H-2	**NDM + OXA-48-like**	147	1	*wzi420*	KL10	*ybt1, ICEKp4*	1
NDM-Kpn-345	H-2	**NDM**	11	10	*wzi24*	KL24	*ybt15, ICEKp11*	1
NDM-Kpn-346	H-2	**NDM**	11	10	*wzi24*	KL24	*ybt15, ICEKp11*	1
NDM-Kpn-347	H-2	**NDM**	11	10	*wzi24*	KL24	*ybt15, ICEKp11*	1
NDM-Kpn-348	H-2	**NDM + OXA-48-like**	147	9	-	KL64	*rmp1, rmpA2, ybt9, ICEKp?, iuc1*	4
NDM-Kpn-349	H-16	**NDM + OXA-48-like**	147	1	*wzi420*	KL10	*ybt1, ICEKp4*	1
NDM-Kpn-350	H-12	**NDM**	147	2	*wzi64*	KL64		
