# Supplementary material for: Emergence and Dissemination of NDM+OXA-48-like Co-Producing Klebsiella pneumoniae in a Regional Healthcare Network: Seven-Year Surveillance from Latium, Italy
Source: Antibiotics (Basel). 2026 Aug 10;15(8):766. doi: 10.3390/antibiotics15080766 (PMC13509432; doi:10.3390/antibiotics15080766)
Supplement: Supplementary file 1 [file antibiotics-15-00766-s001.zip › Table S5-edited.pdf]

Table S5. Major clonal clusters (CT-1 to CT-9) among NDM-Kpn isolates.

CT-1: 166 isolates ST147, and one ST8609

NDM-Kpn-56: *bla*$_{NDM-1}$, *bla*$_{OXA-48}$; virulence score: 1; 3-H; 2023

NDM-Kpn-65: *bla*$_{NDM-1}$, *bla*$_{OXA-48}$, *bla*$_{CTX-M-15}$; virulence score: 1; H-3; 2023

NDM-Kpn-74: *bla*$_{NDM-1}$, *bla*$_{OXA-48}$, *bla*$_{CTX-M-15}$; virulence score: 1; H-9; 2023

NDM-Kpn-77: *bla*$_{NDM-1}$, *bla*$_{OXA-48}$, *bla*$_{CTX-M-15}$; virulence score: 1; H-3; 2023

NDM-Kpn-82: *bla*$_{NDM-1}$, *bla*$_{OXA-48}$, *bla*$_{CTX-M-15}$; virulence score: 1; H-5; 2023

NDM-Kpn-83: *bla*$_{NDM-1}$, *bla*$_{OXA-48}$, *bla*$_{CTX-M-15}$; virulence score: 1; H-9; 2023

NDM-Kpn-85: *bla*$_{NDM-1}$, *bla*$_{OXA-48}$, *bla*$_{CTX-M-15}$; virulence score: 1; H-9; 2023

NDM-Kpn-91: *bla*$_{NDM-1}$, *bla*$_{OXA-48}$, *bla*$_{CTX-M-15}$; virulence score: 1; H-21; 2023

NDM-Kpn-96: *bla*$_{NDM-1}$, *bla*$_{OXA-48}$, *bla*$_{CTX-M-15}$; virulence score: 1; H-21; 2023

NDM-Kpn-103: *bla*$_{NDM-1}$, *bla*$_{OXA-48}$, *bla*$_{CTX-M-15}$; virulence score: 1; H-3; 2023

NDM-Kpn-113: *bla*$_{NDM-1}$, *bla*$_{OXA-48}$, *bla*$_{CTX-M-15}$; virulence score: 1; H-1; 2023

NDM-Kpn-121: *bla*$_{NDM-1}$, *bla*$_{OXA-48}$, *bla*$_{CTX-M-15}$; virulence score: 1; H-7; 2023

NDM-Kpn-126: *bla*$_{NDM-1}$, *bla*$_{OXA-48}$, *bla*$_{CTX-M-15}$; virulence score: 1; H-1; 2023

NDM-Kpn-129: *bla*$_{NDM-1}$, *bla*$_{OXA-48}$, *bla*$_{CTX-M-15}$; virulence score: 1; H-1; 2024

NDM-Kpn-133: *bla*$_{NDM-1}$, *bla*$_{OXA-48}$, *bla*$_{CTX-M-15}$; virulence score: 1; H-3; 2024

NDM-Kpn-134 *bla*$_{NDM-1}$, *bla*$_{OXA-48}$, *bla*$_{CTX-M-15}$; virulence score: 1; H-9; 2024

NDM-Kpn-141: *bla*$_{NDM-1}$, *bla*$_{OXA-48}$, *bla*$_{CTX-M-15}$; virulence score: 1; H-1; 2024

NDM-Kpn-143: *bla*$_{NDM-1}$, *bla*$_{OXA-48}$, *bla*$_{CTX-M-15}$; virulence score: 1; H-15; 2024

NDM-Kpn-146: *bla*$_{NDM-1}$, *bla*$_{OXA-48}$, *bla*$_{CTX-M-15}$; virulence score: 1; H-3; 2024

NDM-Kpn-148: *bla*$_{NDM-1}$, *bla*$_{OXA-48}$, *bla*$_{CTX-M-15}$; virulence score: 1; H-3; 2024

NDM-Kpn-149: *bla*$_{NDM-1}$, *bla*$_{OXA-48}$, *bla*$_{CTX-M-15}$; virulence score: 1; H-6; 2024

NDM-Kpn-150: *bla*$_{NDM-1}$, *bla*$_{OXA-48}$, *bla*$_{CTX-M-15}$; virulence score: 1; H-3; 2024

NDM-Kpn-153: *bla*$_{NDM-1}$, *bla*$_{OXA-48}$, *bla*$_{CTX-M-15}$; virulence score: 1; H-3; 2024

NDM-Kpn-155: *bla*$_{NDM-1}$, *bla*$_{OXA-48}$, *bla*$_{CTX-M-15}$; virulence score: 1; H-3; 2024

NDM-Kpn-156: *bla*$_{NDM-1}$, *bla*$_{OXA-48}$, *bla*$_{CTX-M-15}$; virulence score: 1; H-15; 2024

NDM-Kpn-157: *bla*$_{NDM-1}$, *bla*$_{OXA-48}$, *bla*$_{CTX-M-15}$; virulence score: 1; H-15; 2024

NDM-Kpn-158: *bla*$_{NDM-1}$, *bla*$_{OXA-48}$, *bla*$_{CTX-M-15}$; virulence score: 1; H-10; 2024

NDM-Kpn-159: *bla*$_{NDM-1}$, *bla*$_{OXA-48}$, *bla*$_{CTX-M-15}$; virulence score: 1; H-10; 2024

NDM-Kpn-160: *bla*$_{NDM-1}$, *bla*$_{OXA-48}$, *bla*$_{CTX-M-15}$; virulence score: 1; H-10; 2024

NDM-Kpn-163: *bla*$_{NDM-1}$, *bla*$_{OXA-48}$, *bla*$_{CTX-M-15}$; virulence score: 1; H-3; 2024

NDM-Kpn-165: *bla*$_{NDM-1}$, *bla*$_{OXA-48}$, *bla*$_{CTX-M-15}$; virulence score: 1; H-5; 2024

NDM-Kpn-171: bla_{NDM-1}, bla_{OXA-48}, $bla_{CTX-M-15}$; virulence score: 1; H-2; 2024

NDM-Kpn-174: bla_{NDM-1}, bla_{OXA-48}, $bla_{CTX-M-15}$; virulence score: 1; H-2; 2024

NDM-Kpn-176: bla_{NDM-1}, bla_{OXA-48}, $bla_{CTX-M-15}$; virulence score: 1; H-3; 2024

NDM-Kpn-177: bla_{NDM-1}, bla_{OXA-48}, $bla_{CTX-M-15}$; virulence score: 1; H-3; 2024

NDM-Kpn-178: bla_{NDM-1}, bla_{OXA-48}, $bla_{CTX-M-15}$; virulence score: 1; H-3; 2024

NDM-Kpn-179: bla_{NDM-1}, bla_{OXA-48}, $bla_{CTX-M-15}$; virulence score: 1; H-11; 2024

NDM-Kpn-180: bla_{NDM-1}, bla_{OXA-48}, $bla_{CTX-M-15}$; virulence score: 1; H-14; 2024

NDM-Kpn-191: bla_{NDM-1}, bla_{OXA-48}, $bla_{CTX-M-15}$; virulence score: 1; H-3; 2024

NDM-Kpn-192: bla_{NDM-1}, bla_{OXA-48}, $bla_{CTX-M-15}$; virulence score: 1; H-6; 2024

NDM-Kpn-196: bla_{NDM-1}, bla_{OXA-48}, $bla_{CTX-M-15}$; virulence score: 1; H-5; 2024

NDM-Kpn-201: bla_{NDM-1}, bla_{OXA-48}, $bla_{CTX-M-15}$; virulence score: 1; H-16; 2024

NDM-Kpn-203: bla_{NDM-1}, bla_{OXA-48}, $bla_{CTX-M-15}$; virulence score: 1; H-4; 2024

NDM-Kpn-204: bla_{NDM-1}, bla_{OXA-48}, $bla_{CTX-M-15}$; virulence score: 1; H-4; 2024

NDM-Kpn-205: bla_{NDM-1}, bla_{OXA-48}, $bla_{CTX-M-15}$; virulence score: 1; H-4; 2024

NDM-Kpn-206: bla_{NDM-1}, bla_{OXA-48}, $bla_{CTX-M-15}$; virulence score: 1; H-4; 2024

NDM-Kpn-209: bla_{NDM-1}, bla_{OXA-48}, $bla_{CTX-M-15}$; virulence score: 1; H-2; 2024

NDM-Kpn-210: bla_{NDM-1}, bla_{OXA-48}, $bla_{CTX-M-15}$; virulence score: 1; H-4; 2024

NDM-Kpn-212: bla_{NDM-1}, bla_{OXA-48}, $bla_{CTX-M-15}$; virulence score: 1; H-11; 2024

NDM-Kpn-213: bla_{NDM-1}, bla_{OXA-48}, $bla_{CTX-M-15}$; virulence score: 1; H-11; 2024

NDM-Kpn-214: bla_{NDM-1}, bla_{OXA-48}, $bla_{CTX-M-15}$; virulence score: 1; H-11; 2024

NDM-Kpn-215: bla_{NDM-1}, bla_{OXA-48}, $bla_{CTX-M-15}$; virulence score: 1; H-11; 2024

NDM-Kpn-217: bla_{NDM-1}, bla_{OXA-48}, $bla_{CTX-M-15}$; virulence score: 1; H-6; 2024

NDM-Kpn-219: bla_{NDM-1}, bla_{OXA-48}, $bla_{CTX-M-15}$; virulence score: 1; H-7; 2024

NDM-Kpn-224: bla_{NDM-1}, bla_{OXA-48}, $bla_{CTX-M-15}$; virulence score: 1; H-7; 2024

NDM-Kpn-225: bla_{NDM-1}, $bla_{CTX-M-15}$; virulence score: 1; H-10; 2024

NDM-Kpn-231: bla_{NDM-1}, bla_{OXA-48}, $bla_{CTX-M-15}$; virulence score: 1; H-6; 2024

NDM-Kpn-232: bla_{NDM-1}, bla_{OXA-48}, $bla_{CTX-M-15}$; virulence score: 1; H-6; 2024

NDM-Kpn-233: bla_{NDM-1}, bla_{OXA-48}, $bla_{CTX-M-15}$; virulence score: 1; H-2; 2024

NDM-Kpn-234: bla_{NDM-1}, bla_{OXA-48}, $bla_{CTX-M-15}$; virulence score: 1; H-4; 2024

NDM-Kpn-235: bla_{NDM-1}, bla_{OXA-48}, $bla_{CTX-M-15}$; virulence score: 1; H-4; 2024

NDM-Kpn-236: bla_{NDM-1}, bla_{OXA-48}, $bla_{CTX-M-15}$; virulence score: 1; H-4; 2024

NDM-Kpn-237: bla_{NDM-1}, bla_{OXA-48}, $bla_{CTX-M-15}$; virulence score: 1; H-4; 2024

NDM-Kpn-238: bla_{NDM-1}, bla_{OXA-48}, $bla_{CTX-M-15}$; virulence score: 1; H-4; 2024

NDM-Kpn-239: bla_{NDM-1}, bla_{OXA-48}, $bla_{CTX-M-15}$; virulence score: 1; H-4; 2024

NDM-Kpn-240: *bla*$_{NDM-1}$, *bla*$_{OXA-48}$, *bla*$_{CTX-M-15}$; virulence score: 1; H-7; 2024

NDM-Kpn-242: *bla*$_{NDM-1}$, *bla*$_{OXA-48}$, *bla*$_{CTX-M-15}$; virulence score: 1; H-1; 2024

NDM-Kpn-244: *bla*$_{NDM-1}$, *bla*$_{OXA-48}$, *bla*$_{CTX-M-15}$; virulence score: 1; H-1; 2024

NDM-Kpn-254: *bla*$_{NDM-1}$, *bla*$_{OXA-48}$, *bla*$_{CTX-M-15}$; virulence score: 1; H-5; 2024

NDM-Kpn-255: *bla*$_{NDM-1}$, *bla*$_{OXA-48}$, *bla*$_{CTX-M-15}$; virulence score: 1; H-3; 2024

NDM-Kpn-256: *bla*$_{NDM-1}$, *bla*$_{OXA-48}$, *bla*$_{CTX-M-15}$; virulence score: 1; H-11; 2024

NDM-Kpn-257: *bla*$_{NDM-1}$, *bla*$_{OXA-48}$, *bla*$_{CTX-M-15}$; virulence score: 1; H-11; 2024

NDM-Kpn-258: *bla*$_{NDM-1}$, *bla*$_{OXA-48}$, *bla*$_{CTX-M-15}$; virulence score: 1; H-14; 2024

NDM-Kpn-259: *bla*$_{NDM-1}$, *bla*$_{OXA-48}$, *bla*$_{CTX-M-15}$; virulence score: 1; H-6; 2024

NDM-Kpn-265: *bla*$_{NDM-1}$, *bla*$_{OXA-48}$, *bla*$_{CTX-M-15}$; virulence score: 1; H-1; 2024

NDM-Kpn-270: *bla*$_{NDM-1}$, *bla*$_{OXA-48}$, *bla*$_{CTX-M-15}$; virulence score: 1; H-1; 2024

NDM-Kpn-274: *bla*$_{NDM-1}$, *bla*$_{OXA-48}$, *bla*$_{CTX-M-15}$; virulence score: 1; H-6; 2024

NDM-Kpn-280: *bla*$_{NDM-1}$, *bla*$_{OXA-48}$, *bla*$_{CTX-M-15}$; virulence score: 1; H-14; 2024

NDM-Kpn-288: *bla*$_{NDM-1}$, *bla*$_{OXA-48}$, *bla*$_{CTX-M-15}$; virulence score: 1; H-16; 2024

NDM-Kpn-293: *bla*$_{NDM-1}$, *bla*$_{OXA-48}$, *bla*$_{CTX-M-15}$; virulence score: 1; H-7; 2024

NDM-Kpn-294: *bla*$_{NDM-1}$, *bla*$_{OXA-48}$, *bla*$_{CTX-M-15}$; virulence score: 1; H-14; 2024

NDM-Kpn-295: *bla*$_{NDM-1}$, *bla*$_{OXA-48}$, *bla*$_{CTX-M-15}$; virulence score: 1; H-4; 2024

NDM-Kpn-296: *bla*$_{NDM-1}$, *bla*$_{OXA-48}$, *bla*$_{CTX-M-15}$; virulence score: 1; H-4; 2024

NDM-Kpn-297: *bla*$_{NDM-1}$, *bla*$_{OXA-48}$, *bla*$_{CTX-M-15}$; virulence score: 1; H-4; 2024

NDM-Kpn-299: *bla*$_{NDM-1}$, *bla*$_{OXA-48}$, *bla*$_{CTX-M-15}$; virulence score: 1; H-6; 2024

NDM-Kpn-300: *bla*$_{NDM-1}$, *bla*$_{OXA-48}$, *bla*$_{CTX-M-15}$virulence score: 1; H-8; 2024

NDM-Kpn-305: *bla*$_{NDM-1}$, *bla*$_{OXA-48}$, *bla*$_{CTX-M-15}$; virulence score: 1; H-8; 2024

NDM-Kpn-310: *bla*$_{NDM-1}$, *bla*$_{OXA-48}$, *bla*$_{CTX-M-15}$; virulence score: 1; H-2; 2024

NDM-Kpn-313: *bla*$_{NDM-1}$, *bla*$_{OXA-48}$, *bla*$_{CTX-M-15}$; virulence score: 1; H-6; 2024

NDM-Kpn-315: *bla*$_{NDM-1}$, *bla*$_{OXA-48}$, *bla*$_{CTX-M-15}$; virulence score: 1; H-4; 2024

NDM-Kpn-316: *bla*$_{NDM-1}$, *bla*$_{OXA-48}$, *bla*$_{CTX-M-15}$; virulence score: 1; H-4; 2024

NDM-Kpn-317: *bla*$_{NDM-1}$, *bla*$_{CTX-M-15}$; virulence score: 1; H-4; 2024

NDM-Kpn-318: *bla*$_{NDM-1}$, *bla*$_{OXA-48}$, *bla*$_{CTX-M-15}$; virulence score: 1; H-4; 2024

NDM-Kpn-319: *bla*$_{NDM-1}$, *bla*$_{OXA-48}$, *bla*$_{CTX-M-15}$; virulence score: 1; H-4; 2024

NDM-Kpn-320: *bla*$_{NDM-1}$, *bla*$_{OXA-48}$, *bla*$_{CTX-M-15}$; virulence score: 1; H-2; 2024

NDM-Kpn-322: *bla*$_{NDM-1}$, *bla*$_{OXA-48}$, *bla*$_{CTX-M-15}$; virulence score: 1; H-6; 2024

NDM-Kpn-324: *bla*$_{NDM-1}$, *bla*$_{OXA-48}$, *bla*$_{CTX-M-15}$; virulence score: 1; H-1; 2024

NDM-Kpn-326: *bla*$_{NDM-1}$, *bla*$_{OXA-48}$, *bla*$_{CTX-M-15}$; virulence score: 1; H-1; 2024

NDM-Kpn-329: *bla*$_{NDM-1}$, *bla*$_{OXA-48}$, *bla*$_{CTX-M-15}$; virulence score: 1; H-1; 2024

NDM-Kpn-330: *bla*$_{NDM-1}$, *bla*$_{CTX-M-15}$; virulence score: 1; H-1; 2024

NDM-Kpn-331: *bla*$_{NDM-1}$, *bla*$_{OXA-48}$, *bla*$_{CTX-M-15}$; virulence score: 1; H-1; 2024

NDM-Kpn-336: *bla*$_{NDM-1}$, *bla*$_{OXA-48}$, *bla*$_{CTX-M-15}$; virulence score: 1; H-3; 2024

NDM-Kpn-339: *bla*$_{NDM-1}$, *bla*$_{OXA-48}$, *bla*$_{CTX-M-15}$; virulence score: 1; H-24; 2024

NDM-Kpn-340: *bla*$_{NDM-1}$, *bla*$_{OXA-48}$, *bla*$_{CTX-M-15}$; virulence score: 1; H-6; 2024

NDM-Kpn-341: *bla*$_{NDM-1}$, *bla*$_{OXA-48}$, *bla*$_{CTX-M-15}$; virulence score: 1; H-6; 2024

NDM-Kpn-342: *bla*$_{NDM-1}$, *bla*$_{OXA-48}$, *bla*$_{CTX-M-15}$; virulence score: 1; H-6; 2024

NDM-Kpn-343: *bla*$_{NDM-1}$, *bla*$_{OXA-48}$, *bla*$_{CTX-M-15}$; virulence score: 1; H-2; 2024

NDM-Kpn-344: *bla*$_{NDM-1}$, *bla*$_{OXA-48}$, *bla*$_{CTX-M-15}$; virulence score: 1; H-2; 2024

NDM-Kpn-349: *bla*$_{NDM-1}$, *bla*$_{OXA-48}$, *bla*$_{CTX-M-15}$; virulence score: 1; H-16; 2024

NDM-Kpn-358: *bla*$_{NDM-5}$, *bla*$_{OXA-48}$, *bla*$_{CTX-M-15}$; virulence score: 1; H-6; 2024

NDM-Kpn-361: *bla*$_{NDM-1}$, *bla*$_{OXA-48}$, *bla*$_{CTX-M-15}$; virulence score: 1; H-6; 2024

NDM-Kpn-364: *bla*$_{NDM-1}$, *bla*$_{OXA-48}$, *bla*$_{CTX-M-15}$; virulence score: 1; H-1; 2024

NDM-Kpn-366: *bla*$_{NDM-1}$, *bla*$_{OXA-48}$, *bla*$_{CTX-M-15}$; virulence score: 1; H-1; 2024

NDM-Kpn-367: *bla*$_{NDM-1}$, *bla*$_{OXA-48}$, *bla*$_{CTX-M-15}$; virulence score: 1; H-1; 2024

NDM-Kpn-368: *bla*$_{NDM-1}$, *bla*$_{OXA-48}$, *bla*$_{CTX-M-15}$; virulence score: 1; H-1; 2024

NDM-Kpn-371: *bla*$_{NDM-1}$, *bla*$_{OXA-48}$, *bla*$_{CTX-M-15}$; virulence score: 1; H-1; 2024

NDM-Kpn-374: *bla*$_{NDM-1}$, *bla*$_{OXA-48}$, *bla*$_{CTX-M-15}$; virulence score: 1; H-3; 2024

NDM-Kpn-375: *bla*$_{NDM-1}$, *bla*$_{OXA-48}$, *bla*$_{CTX-M-15}$; virulence score: 1; H-16; 2024

NDM-Kpn-382: *bla*$_{NDM-1}$, *bla*$_{OXA-48}$, *bla*$_{CTX-M-15}$; virulence score: 1; H-4; 2024

NDM-Kpn-383: *bla*$_{NDM-1}$, *bla*$_{OXA-48}$, *bla*$_{CTX-M-15}$; virulence score: 1; H-4; 2024

NDM-Kpn-384: *bla*$_{NDM-1}$, *bla*$_{OXA-48}$, *bla*$_{CTX-M-15}$; virulence score: 1; H-4; 2024

NDM-Kpn-385: *bla*$_{NDM-1}$, *bla*$_{OXA-48}$, *bla*$_{CTX-M-15}$; virulence score: 1; H-4; 2024

NDM-Kpn-386: *bla*$_{NDM-1}$, *bla*$_{OXA-48}$, *bla*$_{CTX-M-15}$; virulence score: 1; H-4; 2024

NDM-Kpn-387: *bla*$_{NDM-1}$, *bla*$_{OXA-48}$, *bla*$_{CTX-M-15}$; virulence score: 1; H-5; 2024

NDM-Kpn-388: *bla*$_{NDM-1}$, *bla*$_{OXA-48}$, *bla*$_{CTX-M-15}$; virulence score: 1; H-2; 2024

NDM-Kpn-389: *bla*$_{NDM-1}$, *bla*$_{OXA-48}$, *bla*$_{CTX-M-15}$; virulence score: 1; H-2; 2024

NDM-Kpn-390: *bla*$_{NDM-1}$, *bla*$_{OXA-48}$, *bla*$_{CTX-M-15}$; virulence score: 1; H-7; 2024

NDM-Kpn-391: *bla*$_{NDM-1}$, *bla*$_{OXA-48}$, *bla*$_{CTX-M-15}$; virulence score: 1; H-7; 2024

NDM-Kpn-392: *bla*$_{NDM-1}$, *bla*$_{OXA-48}$, *bla*$_{CTX-M-15}$; virulence score: 1; H-10; 2025

NDM-Kpn-393: *bla*$_{NDM-1}$, *bla*$_{OXA-48}$, *bla*$_{CTX-M-15}$; virulence score: 1; H-6; 2025

NDM-Kpn-394: *bla*$_{NDM-1}$, *bla*$_{OXA-48}$, *bla*$_{CTX-M-15}$; virulence score: 1; H-2; 2025

NDM-Kpn-395: *bla*$_{NDM-1}$, *bla*$_{OXA-48}$, *bla*$_{CTX-M-15}$; virulence score: 1; H-6; 2025

NDM-Kpn-396: *bla*$_{NDM-1}$, *bla*$_{OXA-48}$, *bla*$_{CTX-M-15}$; virulence score: 1; H-6; 2025

NDM-Kpn-398: *bla*$_{NDM-1}$, *bla*$_{OXA-48}$, *bla*$_{CTX-M-15}$; virulence score: 1; H-8; 2025

NDM-Kpn-399: *bla*$_{NDM-1}$, *bla*$_{OXA-48}$, *bla*$_{CTX-M-15}$virulence score: 1; H-6; 2025

NDM-Kpn-400: *bla*$_{NDM-1}$, *bla*$_{OXA-48}$, *bla*$_{CTX-M-15}$; virulence score: 1; H-15; 2025

NDM-Kpn-401: *bla*$_{NDM-1}$, *bla*$_{OXA-48}$, *bla*$_{CTX-M-15}$; virulence score: 1; H-4; 2025

NDM-Kpn-402: *bla*$_{NDM-1}$, *bla*$_{OXA-48}$, *bla*$_{CTX-M-15}$; virulence score: 1; H-2; 2025

NDM-Kpn-403: *bla*$_{NDM-1}$, *bla*$_{OXA-48}$, *bla*$_{CTX-M-15}$; virulence score: 1; H-7; 2025

NDM-Kpn-404: *bla*$_{NDM-1}$, *bla*$_{OXA-48}$, *bla*$_{CTX-M-15}$; virulence score: 1; H-2; 2025

NDM-Kpn-406: *bla*$_{NDM-1}$, *bla*$_{OXA-48}$, *bla*$_{CTX-M-15}$; virulence score: 1; H-2; 2025

NDM-Kpn-407: *bla*$_{NDM-1}$, *bla*$_{OXA-48}$, *bla*$_{CTX-M-15}$; virulence score: 1; H-2; 2025

NDM-Kpn-408: *bla*$_{NDM-1}$, *bla*$_{OXA-48}$, *bla*$_{CTX-M-15}$; virulence score: 1; H-9; 2025

NDM-Kpn-409: *bla*$_{NDM-1}$, *bla*$_{OXA-48}$, *bla*$_{CTX-M-15}$; virulence score: 1; H-11; 2025

NDM-Kpn-410: *bla*$_{NDM-1}$, *bla*$_{OXA-48}$, *bla*$_{CTX-M-15}$; virulence score: 1; H-2; 2025

NDM-Kpn-411: *bla*$_{NDM-1}$, *bla*$_{OXA-48}$, *bla*$_{CTX-M-15}$; virulence score: 1; H-2; 2025

NDM-Kpn-412: *bla*$_{NDM-1}$, *bla*$_{OXA-48}$, *bla*$_{CTX-M-15}$; virulence score: 1; H-1; 2025

NDM-Kpn-414: *bla*$_{NDM-1}$, *bla*$_{OXA-48}$, *bla*$_{CTX-M-15}$; virulence score: 1; H-1; 2025

NDM-Kpn-415: *bla*$_{NDM-1}$, *bla*$_{OXA-48}$, *bla*$_{CTX-M-15}$; virulence score: 1; H-11; 2025

NDM-Kpn-417: *bla*$_{NDM-1}$, *bla*$_{OXA-48}$, *bla*$_{CTX-M-15}$; virulence score: 1; H-1; 2025

NDM-Kpn-418: *bla*$_{NDM-1}$, *bla*$_{OXA-48}$, *bla*$_{CTX-M-15}$; virulence score: 1; H-1; 2025

NDM-Kpn-419: *bla*$_{NDM-1}$, *bla*$_{OXA-48}$, *bla*$_{CTX-M-15}$; virulence score: 1; H-9; 2025

NDM-Kpn-420: *bla*$_{NDM-1}$, *bla*$_{OXA-48}$, *bla*$_{CTX-M-15}$; virulence score: 1; H-11; 2025

NDM-Kpn-421: *bla*$_{NDM-1}$, *bla*$_{OXA-48}$, *bla*$_{CTX-M-15}$; virulence score: 1; H-2; 2025

NDM-Kpn-423: *bla*$_{NDM-1}$, *bla*$_{OXA-48}$, *bla*$_{CTX-M-15}$virulence score: 1; H-11; 2025

NDM-Kpn-424: *bla*$_{NDM-1}$, *bla*$_{OXA-48}$, *bla*$_{CTX-M-15}$; virulence score: 1; H-1; 2025

NDM-Kpn-425: *bla*$_{NDM-1}$, *bla*$_{OXA-48}$, *bla*$_{CTX-M-15}$; virulence score: 1; H-1; 2025

NDM-Kpn-427: *bla*$_{NDM-1}$, *bla*$_{OXA-48}$, *bla*$_{CTX-M-15}$; virulence score: 1; H-8; 2025

NDM-Kpn-428: *bla*$_{NDM-1}$, *bla*$_{OXA-48}$, *bla*$_{CTX-M-15}$; virulence score: 1; H-1; 2025

NDM-Kpn-429: *bla*$_{NDM-1}$, *bla*$_{OXA-48}$, *bla*$_{CTX-M-15}$; virulence score: 1; H-1; 2025

NDM-Kpn-430: *bla*$_{NDM-1}$, *bla*$_{OXA-48}$, *bla*$_{CTX-M-15}$; virulence score: 1; H-11; 2025

NDM-Kpn-431: *bla*$_{NDM-1}$, *bla*$_{OXA-48}$, *bla*$_{CTX-M-15}$; virulence score: 1; H-6; 2025

NDM-Kpn-432: *bla*$_{NDM-1}$, *bla*$_{OXA-48}$, *bla*$_{CTX-M-15}$; virulence score: 1; H-9; 2025

NDM-Kpn-433: *bla*$_{NDM-1}$, *bla*$_{OXA-48}$, *bla*$_{CTX-M-15}$; virulence score: 1; H-2; 2025

NDM-Kpn-434: *bla*$_{NDM-1}$, *bla*$_{OXA-48}$, *bla*$_{CTX-M-15}$; virulence score: 1; H-6; 2025

NDM-Kpn-435: *bla*$_{NDM-1}$, *bla*$_{OXA-48}$, *bla*$_{CTX-M-15}$; virulence score: 1; H-12; 2025

CT-2: 44 isolates ST147

NDM-Kpn-12: *bla*$_{NDM-1}$, *bla*$_{CTX-M-15}$; virulence score: 4; H-18; 2020

NDM-Kpn-21: *bla*$_{NDM-1}$, *bla*$_{CTX-M-15}$; virulence score: 4; H-5; 2021

NDM-Kpn-31: *bla*$_{NDM-1}$, *bla*$_{CTX-M-15}$; virulence score: 4; H-3; 2021

NDM-Kpn-41: *bla*$_{NDM-1}$, *bla*$_{CTX-M-15}$; virulence score: 4; H-2; 2022

NDM-Kpn-59: *bla*$_{NDM-1}$, *bla*$_{CTX-M-15}$; virulence score: 4; H-28; 2023

NDM-Kpn-60: *bla*$_{NDM-1}$, *bla*$_{CTX-M-15}$; virulence score: 4; H-14; 2023

NDM-Kpn-61: *bla*$_{NDM-1}$, *bla*$_{CTX-M-15}$; virulence score: 4; H-18; 2023

NDM-Kpn-66: *bla*$_{NDM-1}$, *bla*$_{CTX-M-15}$; virulence score: 4; H-17; 2023

NDM-Kpn-67: *bla*$_{NDM-1}$, *bla*$_{CTX-M-15}$; virulence score: 4; H-17; 2023

NDM-Kpn-68: *bla*$_{NDM-1}$, *bla*$_{CTX-M-15}$; virulence score: 4; H-5; 2023

NDM-Kpn-69: *bla*$_{NDM-1}$, *bla*$_{CTX-M-15}$; virulence score: 4; H-17; 2023

NDM-Kpn-75: *bla*$_{NDM-1}$, *bla*$_{CTX-M-15}$; virulence score: 1; H-3; 2023

NDM-Kpn-81: *bla*$_{NDM-1}$, *bla*$_{CTX-M-15}$; virulence score: 4; H-8; 2023

NDM-Kpn-86: *bla*$_{NDM-1}$, *bla*$_{CTX-M-15}$; virulence score: 4; H-7; 2023

NDM-Kpn-120: *bla*$_{NDM-1}$, *bla*$_{CTX-M-15}$; virulence score: 4; H-3; 2023

NDM-Kpn-128: *bla*$_{NDM-1}$, *bla*$_{CTX-M-15}$; virulence score: 4; H-2; 2024

NDM-Kpn-132: *bla*$_{NDM-1}$, *bla*$_{CTX-M-15}$; virulence score: 4; H-2; 2024

NDM-Kpn-145: *bla*$_{NDM-1}$, *bla*$_{CTX-M-15}$; virulence score: 4; H-12; 2024

NDM-Kpn-166: *bla*$_{NDM-1}$, *bla*$_{CTX-M-15}$; virulence score: 4; H-2; 2024

NDM-Kpn-182: *bla*$_{NDM-1}$, *bla*$_{CTX-M-15}$; virulence score: 1; H-3; 2024

NDM-Kpn-193: *bla*$_{NDM-1}$, *bla*$_{CTX-M-15}$; virulence score: 4; H-8; 2024

NDM-Kpn-220: *bla*$_{NDM-1}$, *bla*$_{CTX-M-15}$; virulence score: 4; H-20; 2024

NDM-Kpn-223: *bla*$_{NDM-1}$, *bla*$_{CTX-M-15}$; virulence score: 1; H-3; 2024

NDM-Kpn-230: *bla*$_{NDM-1}$, *bla*$_{CTX-M-15}$; virulence score: 1; H-14; 2024

NDM-Kpn-260: *bla*$_{NDM-1}$, *bla*$_{CTX-M-15}$; virulence score: 1; H-1; 2024

NDM-Kpn-271: *bla*$_{NDM-1}$, *bla*$_{CTX-M-15}$; virulence score: 4; H-5; 2024

NDM-Kpn-272: *bla*$_{NDM-1}$, *bla*$_{CTX-M-15}$; virulence score: 4; H-5; 2024

NDM-Kpn-276: *bla*$_{NDM-1}$, *bla*$_{CTX-M-15}$; virulence score: 4; H-10; 2024

NDM-Kpn-277: *bla*$_{NDM-1}$, *bla*$_{CTX-M-15}$; virulence score: 1; H-10; 2024

NDM-Kpn-278: *bla*$_{NDM-1}$, *bla*$_{CTX-M-15}$; virulence score: 1; H-10; 2024

NDM-Kpn-283: *bla*$_{\text{NDM-1}}$, *bla*$_{\text{CTX-M-15}}$; virulence score: 4; H-12; 2024

NDM-Kpn-284: *bla*$_{\text{NDM-1}}$, *bla*$_{\text{CTX-M-15}}$; virulence score: 4; H-12; 2024

NDM-Kpn-285: *bla*$_{\text{NDM-1}}$, *bla*$_{\text{CTX-M-15}}$; virulence score: 4; H-12; 2024

NDM-Kpn-287: *bla*$_{\text{NDM-1}}$, *bla*$_{\text{CTX-M-15}}$; virulence score: 4; H-12; 2024

NDM-Kpn-302: *bla*$_{\text{NDM-1}}$, *bla*$_{\text{CTX-M-15}}$; virulence score: 4; H-8; 2024

NDM-Kpn-304: *bla*$_{\text{NDM-1}}$, *bla*$_{\text{CTX-M-15}}$; virulence score: 4; H-8; 2024

NDM-Kpn-307: *bla*$_{\text{NDM-1}}$, *bla*$_{\text{CTX-M-15}}$; virulence score: 4; H-8; 2024

NDM-Kpn-309: *bla*$_{\text{NDM-1}}$, *bla*$_{\text{CTX-M-15}}$; virulence score: 4; H-3; 2024

NDM-Kpn-333: *bla*$_{\text{NDM-1}}$, *bla*$_{\text{CTX-M-15}}$; virulence score: 1; H-10; 2024

NDM-Kpn-350: *bla*$_{\text{NDM-1}}$, *bla*$_{\text{CTX-M-15}}$; virulence score: 1; H-12; 2024

NDM-Kpn-351: *bla*$_{\text{NDM-1}}$, *bla*$_{\text{CTX-M-15}}$; virulence score: 4; H-12; 2024

NDM-Kpn-360: *bla*$_{\text{NDM-1}}$, *bla*$_{\text{CTX-M-15}}$; virulence score: 4; H-14; 2024

NDM-Kpn-376: *bla*$_{\text{NDM-1}}$, *bla*$_{\text{CTX-M-15}}$; virulence score: 4; H-12; 2024

NDM-Kpn-378: *bla*$_{\text{NDM-1}}$, *bla*$_{\text{CTX-M-15}}$; virulence score: 4; H-3; 2024

CT-3: 30 isolates ST147

NDM-Kpn-4: *bla*$_{\text{NDM-1}}$, *bla*$_{\text{CTX-M-15}}$; virulence score: 4; H-13; 2019

NDM-Kpn-49: *bla*$_{\text{NDM-1}}$, *bla*$_{\text{CTX-M-15}}$; virulence score: 1; H-7; 2022

NDM-Kpn-87: *bla*$_{\text{NDM-1}}$, *bla*$_{\text{VIM-1}}$, *bla*$_{\text{CTX-M-15}}$; virulence score: 1; H-2; 2023

NDM-Kpn-88: *bla*$_{\text{NDM-1}}$, *bla*$_{\text{VIM-1}}$, *bla*$_{\text{CTX-M-15}}$; virulence score: 1; H-2; 2023

NDM-Kpn-90: *bla*$_{\text{NDM-1}}$, *bla*$_{\text{VIM-1}}$, *bla*$_{\text{CTX-M-15}}$; virulence score: 1; H-3; 2023

NDM-Kpn-93: *bla*$_{\text{NDM-1}}$, *bla*$_{\text{VIM-1}}$, *bla*$_{\text{CTX-M-15}}$; virulence score: 1; H-3; 2023

NDM-Kpn-102: *bla*$_{\text{NDM-1}}$, *bla*$_{\text{VIM-1}}$, *bla*$_{\text{CTX-M-15}}$; virulence score: 1; H-2; 2023

NDM-Kpn-116: *bla*$_{\text{NDM-1}}$, *bla*$_{\text{VIM-1}}$, *bla*$_{\text{CTX-M-15}}$; virulence score: 1; H-2; 2023

NDM-Kpn-119: *bla*$_{\text{NDM-1}}$, *bla*$_{\text{CTX-M-15}}$; virulence score: 4; H-22; 2023

NDM-Kpn-123: *bla*$_{\text{NDM-1}}$, *bla*$_{\text{VIM-1}}$, *bla*$_{\text{CTX-M-15}}$; virulence score: 1; H-2; 2023

NDM-Kpn-135: *bla*$_{\text{NDM-1}}$, *bla*$_{\text{VIM-1}}$, *bla*$_{\text{CTX-M-15}}$; virulence score: 1; H-2; 2024

NDM-Kpn-137: *bla*$_{\text{NDM-1}}$, *bla*$_{\text{VIM-1}}$, *bla*$_{\text{CTX-M-15}}$; virulence score: 1; H-2; 2024

NDM-Kpn-144: *bla*$_{\text{NDM-1}}$, *bla*$_{\text{VIM-1}}$, *bla*$_{\text{CTX-M-15}}$; virulence score: 1; H-5; 2024

NDM-Kpn-147: *bla*$_{\text{NDM-1}}$, *bla*$_{\text{VIM-1}}$, *bla*$_{\text{CTX-M-15}}$; virulence score: 1; H-2; 2024

NDM-Kpn-152: *bla*$_{\text{NDM-1}}$, *bla*$_{\text{VIM-1}}$, *bla*$_{\text{CTX-M-15}}$; virulence score: 1; H-2; 2024

NDM-Kpn-162: *bla*$_{\text{NDM-1}}$, *bla*$_{\text{VIM-1}}$, *bla*$_{\text{CTX-M-15}}$; virulence score: 1; H-5; 2024

NDM-Kpn-164: *bla*$_{\text{NDM-1}}$, *bla*$_{\text{VIM-1}}$, *bla*$_{\text{CTX-M-15}}$; virulence score: 1; H-2; 2024

NDM-Kpn-167: *bla*$_{\text{NDM-1}}$, *bla*$_{\text{VIM-1}}$, *bla*$_{\text{CTX-M-15}}$; virulence score: 1; H-2; 2024

NDM-Kpn-173: *bla*$_{NDM-1}$, *bla*$_{VIM-1}$, *bla*$_{CTX-M-15}$; virulence score: 1; H-2; 2024

NDM-Kpn-181: *bla*$_{NDM-1}$, *bla*$_{VIM-1}$, *bla*$_{CTX-M-15}$; virulence score: 1; H-2; 2024

NDM-Kpn-187: *bla*$_{NDM-1}$, *bla*$_{VIM-1}$, *bla*$_{CTX-M-15}$; virulence score: 1; H-2; 2024

NDM-Kpn-188: *bla*$_{NDM-1}$, *bla*$_{VIM-1}$, *bla*$_{CTX-M-15}$; virulence score: 1; H-2; 2024

NDM-Kpn-189: *bla*$_{NDM-1}$, *bla*$_{VIM-1}$, *bla*$_{CTX-M-15}$; virulence score: 1; H-2; 2024

NDM-Kpn-200: *bla*$_{NDM-1}$, *bla*$_{VIM-1}$, *bla*$_{CTX-M-15}$; virulence score: 1; H-2; 2024

NDM-Kpn-208: *bla*$_{NDM-1}$, *bla*$_{VIM-1}$, *bla*$_{CTX-M-15}$; virulence score: 1; H-2; 2024

NDM-Kpn-211: *bla*$_{NDM-1}$, *bla*$_{VIM-1}$, *bla*$_{CTX-M-15}$; virulence score: 1; H-5; 2024

NDM-Kpn-229: *bla*$_{NDM-1}$, *bla*$_{VIM-1}$, *bla*$_{CTX-M-15}$; virulence score: 1; H-5; 2024

NDM-Kpn-301: *bla*$_{NDM-1}$, *bla*$_{CTX-M-15}$; virulence score: 1; H-8; 2024

NDM-Kpn-314: *bla*$_{NDM-1}$, *bla*$_{VIM-1}$, *bla*$_{CTX-M-15}$; virulence score: 1; H-5; 2024

NDM-Kpn-335: *bla*$_{NDM-1}$, *bla*$_{VIM-1}$, *bla*$_{CTX-M-15}$; virulence score: 1; H-5; 2024

CT-4: 12 isolates ST147

NDM-Kpn-248: *bla*$_{NDM-5}$, *bla*$_{OXA-48}$, *bla*$_{CTX-M-15}$; virulence score: 1; H-1; 2024

NDM-Kpn-269: *bla*$_{NDM-5}$, *bla*$_{OXA-48}$, *bla*$_{CTX-M-15}$; virulence score: 1; H-1; 2024

NDM-Kpn-332: *bla*$_{NDM-5}$, *bla*$_{OXA-48}$, *bla*$_{CTX-M-15}$; virulence score: 1; H-1; 2024

NDM-Kpn-353: *bla*$_{NDM-5}$, *bla*$_{OXA-48}$, *bla*$_{CTX-M-15}$; virulence score: 1; H-1; 2024

NDM-Kpn-362: *bla*$_{NDM-5}$, *bla*$_{OXA-48}$, *bla*$_{CTX-M-15}$; virulence score: 1; H-1; 2024

NDM-Kpn-369: *bla*$_{NDM-5}$, *bla*$_{OXA-48}$, *bla*$_{CTX-M-15}$; virulence score: 1; H-1; 2024

NDM-Kpn-370: *bla*$_{NDM-5}$, *bla*$_{OXA-48}$, *bla*$_{CTX-M-15}$; virulence score: 1; H-1; 2024

NDM-Kpn-397: *bla*$_{NDM-5}$, *bla*$_{OXA-48}$, *bla*$_{CTX-M-15}$; virulence score: 1; H-1; 2025

NDM-Kpn-413: *bla*$_{NDM-5}$, *bla*$_{OXA-48}$, *bla*$_{CTX-M-15}$; virulence score: 1; H-1; 2025

NDM-Kpn-422: *bla*$_{NDM-5}$, *bla*$_{OXA-48}$, *bla*$_{CTX-M-15}$; virulence score: 1; H-1; 2025

NDM-Kpn-436: *bla*$_{NDM-5}$, *bla*$_{OXA-48}$, *bla*$_{CTX-M-15}$; virulence score: 1; H-12; 2025

NDM-Kpn-437: *bla*$_{NDM-5}$, *bla*$_{OXA-48}$, *bla*$_{CTX-M-15}$; virulence score: 1; H-1; 2025

CT-5: 5 isolates ST147

NDM-Kpn-23: *bla*$_{NDM-5}$, *bla*$_{CTX-M-15}$; virulence score: 0; H-7; 2021

NDM-Kpn-25: *bla*$_{NDM-5}$, *bla*$_{CTX-M-15}$; virulence score: 0; H-7; 2021

NDM-Kpn-26: *bla*$_{NDM-5}$, *bla*$_{CTX-M-15}$; virulence score: 0; H-7; 2021

NDM-Kpn-27: *bla*$_{NDM-5}$, *bla*$_{CTX-M-15}$; virulence score: 0; H-7; 2021

NDM-Kpn-29: *bla*$_{NDM-5}$, *bla*$_{CTX-M-15}$; virulence score: 0; H-7; 2021

CT-6: 4 isolates ST147

NDM-Kpn-45: *bla*$_{NDM-1}$, *bla*$_{CTX-M-15}$; virulence score: 1; H-2; 2022

NDM-Kpn-46: *bla*$_{NDM-1}$, *bla*$_{CTX-M-15}$; virulence score: 1; H-2; 2022

NDM-Kpn-48: *bla*$_{NDM-1}$, *bla*$_{CTX-M-15}$; virulence score: 1; H-2; 2022

NDM-Kpn-51: *bla*$_{NDM-1}$, *bla*$_{CTX-M-15}$; virulence score: 1; H-2; 2022

CT-7: 3 isolates ST147

NDM-Kpn-112: *bla*$_{NDM-5}$, *bla*$_{CTX-M-15}$, *bla*$_{CMY-2}$; virulence score: 4; H-1; 2023

NDM-Kpn-136: *bla*$_{NDM-5}$, *bla*$_{CTX-M-15}$, *bla*$_{CMY-2}$; virulence score: 4; H-2; 2024

NDM-Kpn-290: *bla*$_{NDM-5}$, *bla*$_{CTX-M-15}$, *bla*$_{CMY-2}$; virulence score: 4; H-1; 2024

CT-8: 2 isolates ST147

NDM-Kpn-14: *bla*$_{NDM-5}$, *bla*$_{CTX-M-15}$; virulence score: 0; H-5; 2020

NDM-Kpn-16: *bla*$_{NDM-5}$, *bla*$_{CTX-M-15}$; virulence score: 0; H-5; 2020

CT-9: 2 isolates ST147

NDM-Kpn-221: *bla*$_{NDM-5}$, *bla*$_{OXA-48}$, *bla*$_{CTX-M-15}$; virulence score: 4; H-7; 2024

NDM-Kpn-348: *bla*$_{NDM-5}$, *bla*$_{OXA-48}$, *bla*$_{CTX-M-15}$; virulence score: 4; H-2; 2024
